# Regioselective Monoborylation of Spirocyclobutenes

**DOI:** 10.1021/acs.orglett.1c02645

**Published:** 2021-09-15

**Authors:** Luis Nóvoa, Laura Trulli, Israel Fernández, Alejandro Parra, Mariola Tortosa

**Affiliations:** † Departamento de Química Orgánica and Institute for Advanced Research in Chemical Sciences (IAdChem), Universidad Autónoma de Madrid, Madrid 28049, Spain; ‡ Departamento de Química Orgánica I y Centro de Innovación en Química Avanzada (ORFEO−CINQA), Facultad de Ciencias Químicas, Universidad Complutense de Madrid, 28040 Madrid, Spain

## Abstract

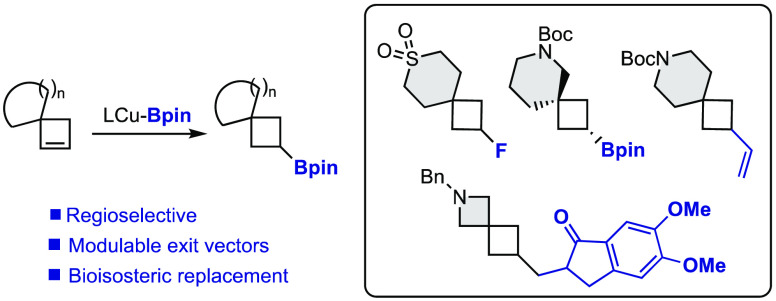

We present a strategy for the synthesis of spirocyclic cyclobutanes with modulable exit vectors based on the regioselective monoborylation of spirocyclobutenes. Using an inexpensive copper salt and a commercially available bidentate phosphine, a broad variety of borylated spirocycles have been prepared with complete regiocontrol. The boryl moiety provides a synthetic handled for further functionalization, allowing access to a wide array of spirocyclic building blocks from a common intermediate.

The development of tools that allow for the modulation of the physical and biological properties of lead compounds is an essential task in drug discovery programs. In this scenario, spirocyclic compounds are receiving great attention, especially those containing small rings.^1^ Among them, cyclobutane-containing spirocycles represent an interesting subclass, as they provide rigidity and tridimensionality with well-defined exit vectors.^2^ One of the most common strategies to build these spirocycles involves an intramolecular S_N_2 reaction or intramolecular addition to a carbonyl (Scheme 1).^3^ One of the drawbacks of this approach is that it is linear in design. For each spirocycle prepared, a different precursor is needed. Additionally, there is very little room to introduce substituents in the cyclobutane ring. A second strategy widely used to prepare spirocyclobutanes is the [2 + 2] or higher-order cycloaddition reaction, starting from an exocyclic alkene.^4^ Cyclopropanes have also been used to build the four-membered ring through ring expansion, starting from especially design precursors.^5^ These two approaches have the advantage of introducing structural complexity in a single step but only allow access to very specific scaffolds (Scheme 1).

**Scheme 1:**
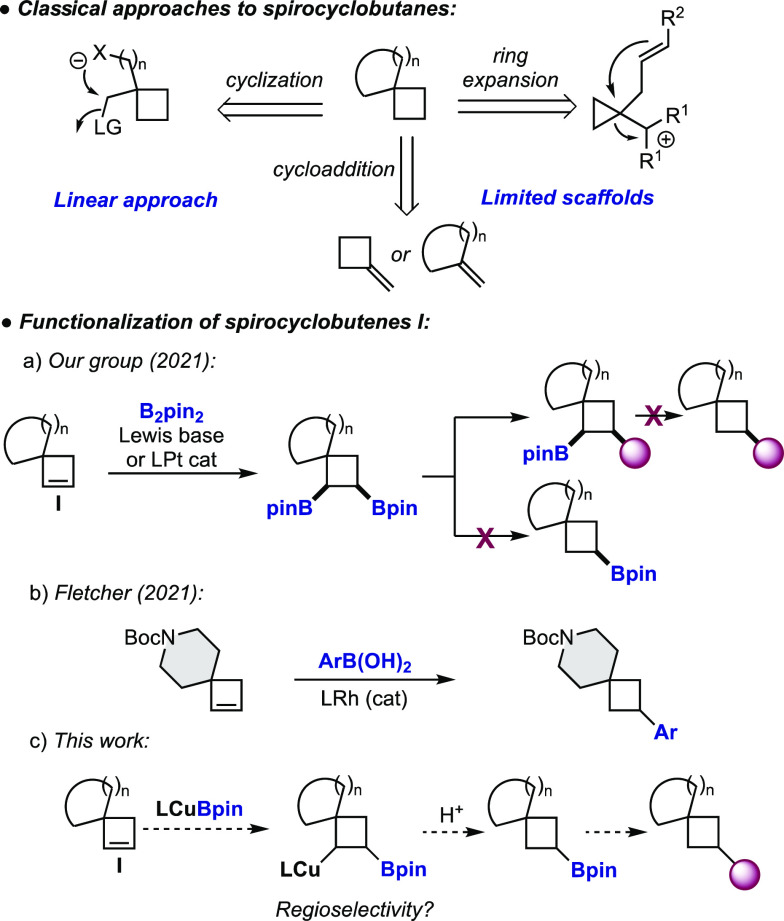
Synthesis of Spirocyclobutanes

Recently, we wondered if spirocyclobutenes I could serve as a template for the preparation of functionalized spirocycles through selective functionalization of the double bond. Although a few spirocyclobutenes had been prepared in the literature, we were surprised to find that their use in catalytic transformations remained at the time virtually unexplored. With this idea, we recently developed the diboration of spirocyclobutenes I promoted by a Lewis base or a platinum catalyst (Scheme 1, a).^6^

Selective functionalization of the two boryl moieties in the products allowed us to prepare a wide array of spirocyclic building blocks, 2,3-disubstituted, with control in the nature and the directionality of the substituents in the cyclobutane ring. One of the limitations that we found was the inability to monofunctionalize the double bond using this approach. Despite significant experimentation, we could not prepare 3-monosubstituted derivatives through protodeboration of diborylated or monoborylated products (Scheme 1, a). During the preparation of this manuscript, in the context of a wider study, Fletcher and co-workers reported an elegant regioselective rhodium-catalyzed hydroarylation of spirocyclic piperidine and pyrrolidine derivatives (Scheme 1, b).^7^ We thought that the monoborylation of spirocyclobutenes could provide access to a variety of novel drug-like building blocks that nicely complement those prepared through diboration and hydroarylation.

Inspired by our previous work on the enantioselective desymmetrization of *meso* cyclobutenes,^8^ we envisioned that *in situ* generated copper–boryl complexes could react with the strained alkene in I through a migratory insertion/protonation sequence to provide monoborylated spirocycles (Scheme 1, c).^9^ The main challenge here, that was not present in our previous study, was the control of the regioselectivity in the insertion step.^10^

At the outset of our study, it was not obvious that the presence of the spirocyclic quaternary carbon would discriminate between the ligand–copper unit and the boryl moiety of the copper–boryl complex. Indeed, we found that the ligand had a profound effect on the regioselectivity outcome (Table 1). Mixing spirocyclobutene **1a** with the NHC–CuCl complex **L**_**1**_–CuCl (10 mmol %), B_2_pin_2_ (1.1 equiv), and MeOH (2 equiv) in THF provided monoborylated compounds **2a** and **3a** in good yield but with low regioselectivity (65:35 mixture, Table 1, entry 1). Using dppbz (**L**_**2**_), a bidentate phosphine with a small bite angle (β_n_ = 83°), a 50:50 mixture of regioisomers was obtained (Table 1, entry 2). Remarkably, **L**_**3**_ xantphos (β_n_ = 108°) afforded borylated spirocycle **2a** in 86% yield as a single regioisomer (Table 1, entry 3). Dppp (**L**_**4**_, β_n_ = 91°), dppf (**L**_**5**_, β_n_ = 99°), and BINAP (**L**_**6**_, β_n_ = 93°), bidentate phosphines with bite angles between **L**_**2**_ and **L**_**3**_, provided moderate regioselectivities (Table 1, entries 4–6). The catalyst loading could be reduced to 5 mol %, providing **2a** as a single regioisomer although in lower yield. We also tested the possibility of preparing boronic ester **2a** through borylation of the corresponding cyclobutyl bromide.^11^ Under the conditions optimized for the cyclobutene, compound **2a** was obtained in 39% yield.^12^ The structural assignment of regioisomer **2a** was confirmed by oxidation of the C–B bond and comparison of the ^1^H NMR data of the product with those of the same alcohol prepared through reduction of the corresponding cyclobutanone.^12^

**Table 1:**
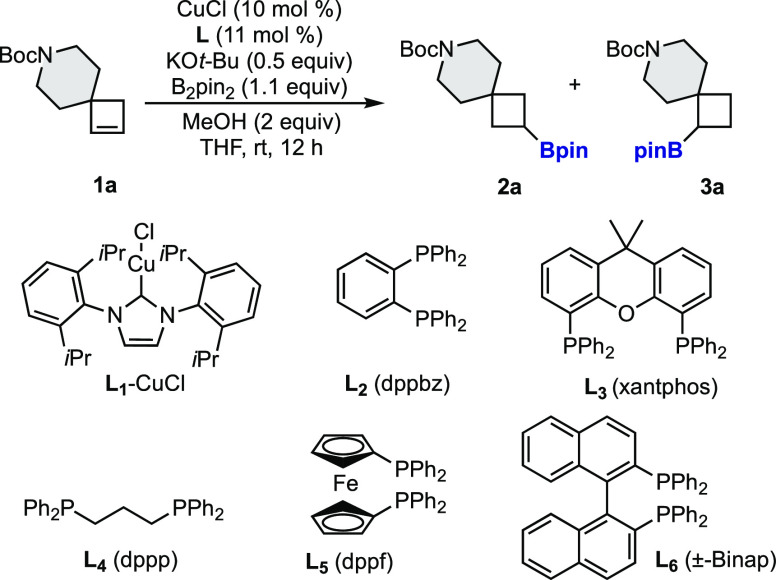
Effect of the Ligand in the Copper-Catalyzed Borylation

entry^a^	**L**	**2a**:**3a**^b^	yield (%)^c^
1	**L**_**1**_	65:35	71
2	**L**_**2**_	50:50	84
**3**	**L**_**3**_	**≥98:2**	**86**
4	**L**_**4**_	83:17	58
5	**L**_**5**_	64:36	79
6	**L**_**6**_	73:27	69
7^d^	**L**_**3**_	≥98:2	69
8	—	60:40	13

a Reaction conditions: **1a** (0.1 mmol), B_2_pin_2_ (0.11 mmol), KO*t*-Bu (0.5 equiv), CuCl (10 mol %), **L** (11 mol %), MeOH (0.2 mmol), THF (0.2 M).

b Determined by ^1^H NMR.

c Isolated yields.

d With 5 mol % of CuCl and 6 mol % of **L**_**3**_.

With these conditions in hand, we prepared in a straightforward manner a wide variety of novel borylated building blocks (Scheme 2). Monoborylated spiro[3.5]nonanes (**2a**–**2h**) with different functional groups were synthesized. Ether, thioether, sulfone, sulfonamide, difluoromethane, and acetal are different connectors that can be embedded in the spirocyclic framework. Additionally, the size of the ring attached to the cyclobutene could be modified. Spiro[3.3]heptane (**2j**, **2k**) and spiro[3.6]decane (**2i**) ring systems were prepared as single regioisomers in good yields. Moreover, a nonsymmetric spirocyclobutene afforded diborylated spirocycle **2l** as a single diastereomer.^13^

**Scheme 2:**
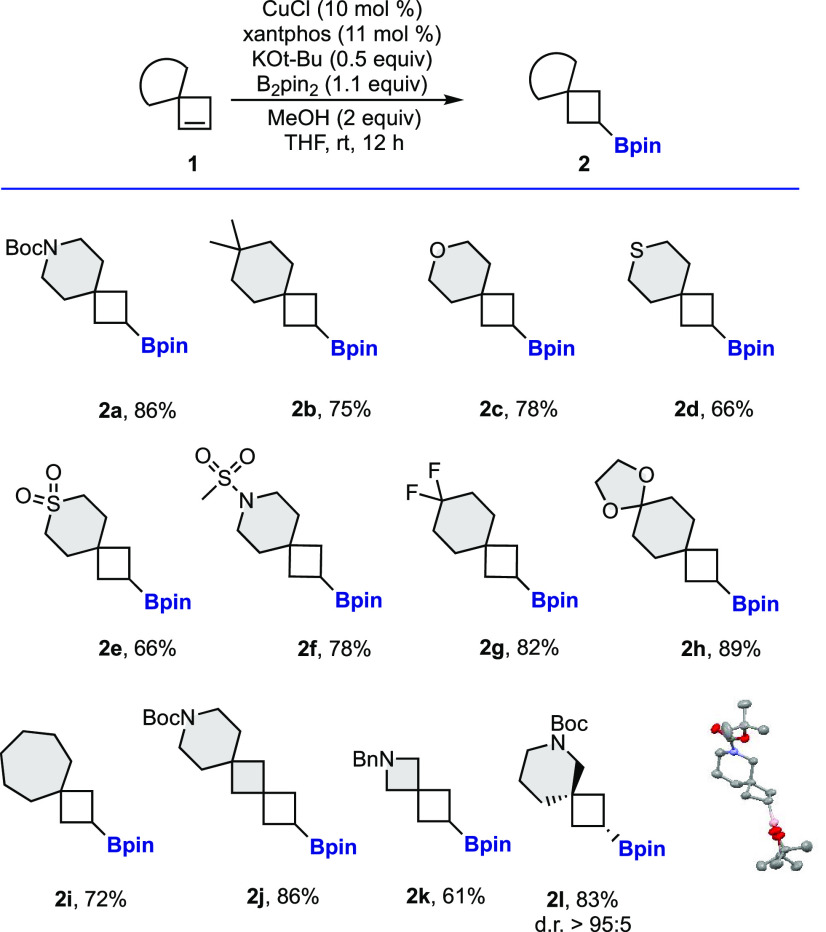
Regioselective Borylation of Spirocyclobutenes^,^ Reaction conditions: **1** (0.2 mmol), B_2_pin_2_ (0.22 mmol), KO*t*-Bu (0.5 equiv), CuCl (10 mol %), xantphos (11 mol %), MeOH (0.4 mmol), and THF (0.2 M). Isolated yields.

Density functional theory (DFT) calculations were carried out at the dispersion-corrected PCM (tetrahydrofuran)/B3LYP-D3/def2-SVP level (see computational details in the SI) to understand the complete regioselectivity observed in the transformation when xantphos was used as a ligand. According to the computed reaction profile involving **1c** and **L**_**3**_Cu-Bpin (Figure 1), the regioselectivity takes place in the initial migratory insertion step, where the associated transition state leading to the observed regioisomer (**TS1-L**_**3**_) lies 4.6 kcal/mol below that leading to the opposite regioisomer (**TS1′-L3**). This is very likely due to unfavorable steric interactions between the BPin and tetrahydropyran fragments in the latter saddle point which are not present in the favored **TS1-L3**.^14^

**Figure 1:**
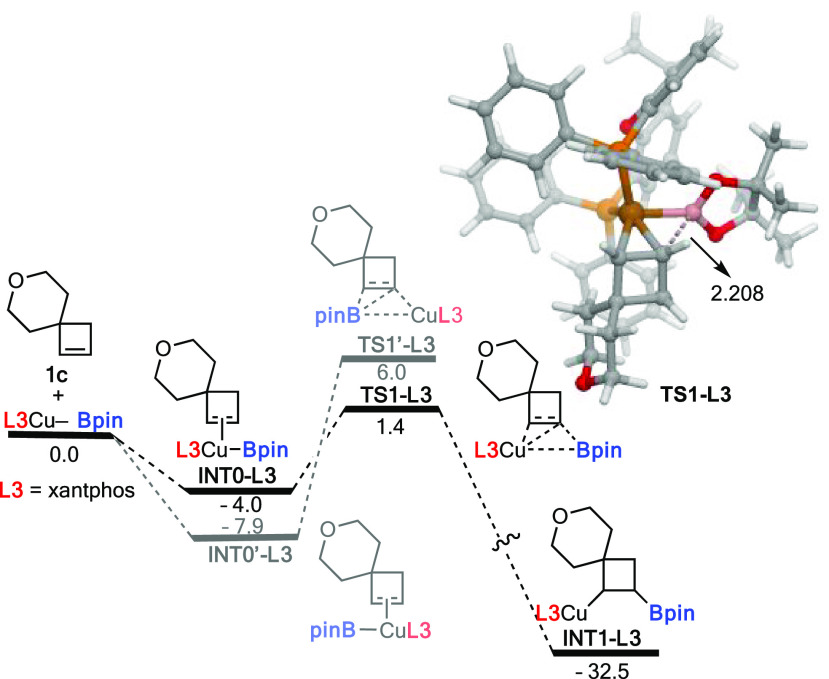
Computed reaction profile for the copper-catalyzed borylation of **1c**. Relative free energies (Δ*G*, at 298 K) are given in kcal/mol. All data have been computed at the PCM (tetrahydrofuran)/B3LYP-D3/def2-SVP level.

The preparation of the spirocyclobutenes and the copper-catalyzed borylation was effectively scaled up to prepare compounds **1m** and **2m** (Scheme 3). The borylation of **1g** of cyclobutene **1m** afforded borylated spirocycle **2m** in 87% yield (Scheme 3).

**Scheme 3:**
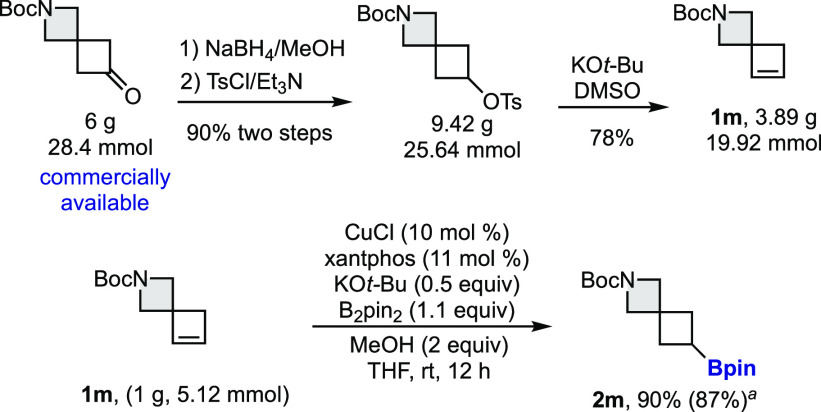
Gram-Scale Reactions Yield starting from 0.2 mmol of **1m**.

One of the interesting features of small ring spirocycles is their potential use for bioisosteric replacement of commonly used heterocycles.^2^ In particular, the 2-azaspiro[3.3]heptane ring present in compound **2m** has been proposed as a bioisostere of the piperidine ring with improved water solubility.^1b^ 4-Substituted piperidines are widely present in commercialized drugs and lead compounds.^15^ Spirocycle **2m**, with two handles for diversification, represents an ideal novel building block to substitute the piperidine ring for the homospiro moiety in libraries of compounds (Scheme 4). To highlight the synthetic potential of this approach, we have prepared a conformationally restricted analogue of the FDA-approved drug donepezil in which the 4-substituted piperidine ring has been replaced by the homospiro piperidine framework. Starting from monoborylated spirocycle **2m**, Matteson homologation followed by double oxidation afforded aldehyde **4**. Then, aldol reaction, dehydration, and hydrogenation of the double bond provided intermediate **5**. Finally, deprotection of the azaspirocycle and benzylation afforded donepezil derivative **6**.

**Scheme 4:**
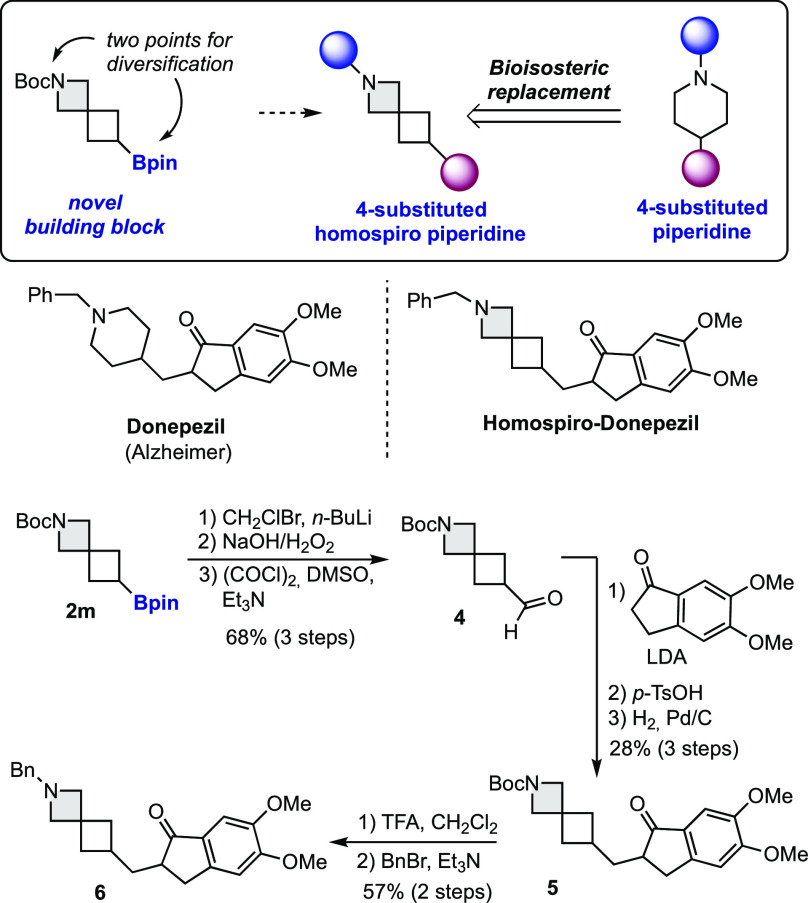
Synthesis of Homospiro-donepezil

Finally, we have explored the possibility to introduce different functional groups in the spirocycle through C–B bond functionalization. Zweifel olefination (compound **7**),^16^ Aggarwal’s cross coupling (compound **8**),^17^ trifluoroborate formation (compound **9**), Matteson homologation (compound **10**), and fluorination (compound **11**)^18^ have been efficiently carried out with spirocyles **2** (Scheme 5). These transformations highlight the potential of the method to prepare a broad set of novel spirocyclic compounds from a common intermediate.

**Scheme 5:**
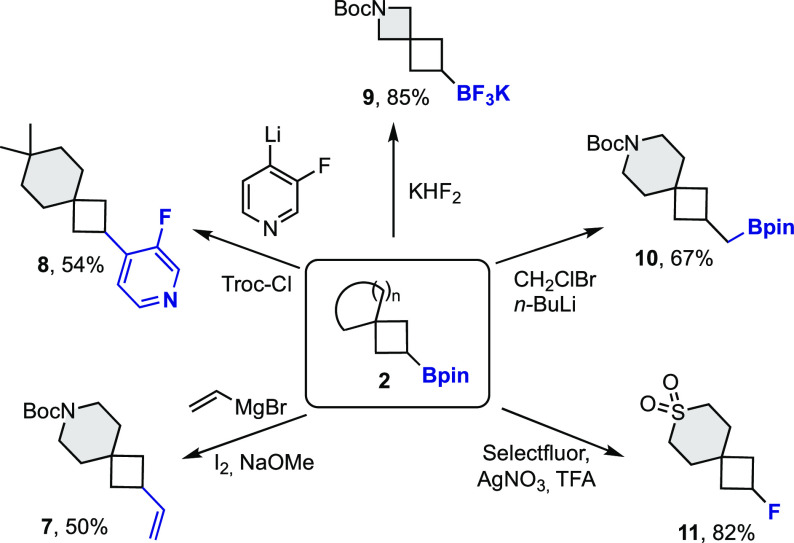
Carbon–Boron Bond Functionalization

In summary, we have shown that spirocyclobutenes are suitable substrates for the regioselective preparation of monoborylated spirocycles under copper-catalyzed conditions. The regioselectivity of the transformation is highly dependent on the ligand used, and it can be controlled completely with xantphos, a commercially available bidentate phosphine. This strategy allows easy access to a broad variety of spirocyclic building blocks, most of them not accessible by known methods.

## Supporting Information Available

Synthetic procedures and full characterization for all new compounds and NMR spectra (PDF)

## Accession Codes

CCDC 2041652 contains the supplementary crystallographic data for this paper. These data can be obtained free of charge via www.ccdc.cam.ac.uk/data_request/cif, or by emailing data_request@ccdc.cam.ac.uk, or by contacting The Cambridge Crystallographic Data Centre, 12 Union Road, Cambridge CB2 1EZ, UK; fax: +44 1223 336033.

## References

[ref1] aLovering F.; Bikker J.; Humblet C. Escape from Flatland: Increasing Saturation as an Approach to Improving Clinical Success. J. Med. Chem. 2009, 52, 6752–6756. 10.1021/jm901241e. 19827778

[ref2] Carreira E. M.; Fessard T. C. Four-Membered Ring-Containing Spirocycles: Synthetic Strategies and Opportunities. Chem. Rev. 2014, 114, 8257–8322. 10.1021/cr500127b. 25003801

[ref3] aBurkhard J. A.; Guérot C.; Knust H.; Rogers-Evans M.; Carreira E. M. Synthesis and Structural Analysis of a New Class of Azaspiro[3.3]heptanes as Building Blocks for Medicinal Chemistry. Org. Lett. 2010, 12, 1944–1947. 10.1021/ol1003302. 20356106

[ref4] aQi L.-W.; Yang Y.; Gui Y.-Y.; Zhang Y.; Chen F.; Tian F.; Peng L.; Wang L.-X. Asymmetric Synthesis of 3,3′-Spirooxindoles Fused with Cyclobutanes through Organocatalytic Formal [2 + 2] Cycloadditions under H-Bond-Directing Dienamine Activation. Org. Lett. 2014, 16, 6436–6439. 10.1021/ol503266q. 25494171

[ref5] Zhao C.-G.; Feng Z.-T.; Xu G.-Q.; Gao A.; Chen J.-W.; Wang Z.-Y.; Xu P.-F. Highly Enantioselective Construction of Strained Spiro[2,3]hexanes through a Michael Addition/Ring Expansion/Cyclization Cascade *Angew*. Angew. Chem., Int. Ed. 2020, 59, 3058–3062. 10.1002/anie.201912834. 31821697

[ref6] Nóvoa L.; Trulli L.; Parra A.; Tortosa M. Stereoselective Diboration of Spirocyclobutenes: A Platform for the Synthesis of Spirocycles with Orthogonal Exit Vectors. Angew. Chem., Int. Ed. 2021, 60, 11763–11768. 10.1002/anie.202101445. 33689223

[ref7] Goetzke F. W.; Hell A. M. L.; van Dijk L.; Fletcher S. P. A Catalytic Asymmetric Cross-coupling Approach to the Synthesis of Cyclobutanes. Nat. Chem. 2021, 10.1038/s41557-021-00725-y. 34211118

[ref8] Guisán-Ceinos M.; Parra A.; Martín-Heras V.; Tortosa M. Enantioselective Synthesis of Cyclobutylboronates via a Copper-Catalyzed Desymmetrization Approach. Angew. Chem., Int. Ed. 2016, 55, 6969–6972. 10.1002/anie.201601976. 27159674

[ref9] For a copper-catalyzed hydroboration of benzylidenecyclobutenes, see: Kang T.; Erbay T. G.; Xu K. L.; Gallego G. M.; Burtea A.; Nair S. K.; Patman R. L.; Zhou R.; Sutton S. C.; McAlpine I. J.; Liu P.; Engle K. M. Multifaceted Substrate–Ligand Interactions Promote the Copper-Catalyzed Hydroboration of Benzylidenecyclobutanes and Related Compounds. ACS Catal. 2020, 10, 13075–13083. 10.1021/acscatal.0c03622. 33791144 PMC8006806

[ref10] aIto H.; Yamanaka H.; Tateiwa J.; Hosomi A. Boration of an α,β-Enone Using a Diboron Promoted by a Copper(I)–Phosphine Mixture Catalyst. Tetrahedron Lett. 2000, 41, 6821–6825. 10.1016/S0040-4039(00)01161-8.

[ref11] Ito H.; Kubota K. Copper(I)-Catalyzed Boryl Substitution of Unactivated Alkyl Halides. Org. Lett. 2012, 14, 890–893. 10.1021/ol203413w. 22260229

[ref12] See Supporting Information for details.

[ref13] The relative stereochemistry of compound **2l** was assigned by single crystal X-ray crystallography. See Supporting Information for details.

[ref14] With dppbz as ligand (**L2**, Table 1), the ΔΔ*G*^≠^ between the corresponding transition states (**TS1-L2** and **TS1′-L2**) decreased to 2 kcal/mol, which is in qualitative agreement with the lower regioselectivity observed for this ligand. See Supporting Information for details.

[ref15] aGoel P.; Alam O.; Naim M. J.; Nawaz F.; Iqbal M.; Alam M. I. Recent advancement of piperidine moiety in treatment of cancer- A review. Eur. J. Med. Chem. 2018, 157, 480–502. 10.1016/j.ejmech.2018.08.017. 30114660

[ref16] Armstrong R. J.; Aggarwal V. K. 50 Years of Zweifel Olefination: A Transition-Metal-Free Coupling. Synthesis 2017, 49, 3323–3336. 10.1055/s-0036-1589046.

[ref17] Llaveria J.; Leonori D.; Aggarwal V. K. Stereospecific Coupling of Boronic Esters with N-Heteroaromatic Compounds. J. Am. Chem. Soc. 2015, 137, 10958–10961. 10.1021/jacs.5b07842. 26292943

[ref18] Li Z.; Wang Z.; Zhu L.; Tan X.; Li C. Silver-Catalyzed Radical Fluorination of Alkylboronates in Aqueous Solution. J. Am. Chem. Soc. 2014, 136, 16439–16443. 10.1021/ja509548z. 25350556

