# Supplementary material for: Regioselective Monoborylation of Spirocyclobutenes
Source: Org Lett. 2021 Sep 15;23(19):7434–8. doi: 10.1021/acs.orglett.1c02645 (PMC8650097; doi:10.1021/acs.orglett.1c02645)
Supplement: Supplementary file 1 — ol1c02645_si_001.pdf [file ol1c02645_si_001.pdf]

Supporting Information

Regioselective Monoborylation of Spirocyclobutenes

Luis Nóvoa,[a‡] Laura Trulli,[a‡] Israel Fernández,[b] Alejandro Parra[a] and Mariola Tortosa[a*]

[a]Departamento de Química Orgánica and Institute for Advanced Research in Chemical Sciences (IAdChem), Universidad Autónoma de Madrid, Madrid 28049, Spain.

[b]Departamento de Química Orgánica I y Centro de Innovación en Química Avanzada (ORFEO-CINQA), Facultad de Ciencias Químicas, Universidad Complutense de Madrid, 28040 Madrid, Spain

e-mail (M. Tortosa): mariola.tortosa@uam.es

Table of contents

1. General Experimental Details 3

2. Syntheis of Starting Materials 4

3. Ligand Optimization 4

4. General Procedure for the Regioselective Monoboration of Spirocyclobutenes 5

5. Gram Scale Experiments 10

6. Bioisosteric Replacement: Synthesis of Donepezil Derivative 11

7. Functionalization of Monoborylated Spirocyles 14

7.1 Vinylation 14

7.2 Arylation 14

7.3 Trifluoroborate salt 15

7.4 Homologation 16

7.5 Fluorination 16

8. Comparison of the Copper-Catalyzed Borylation of Cyclobutene 1a and Bromide SI-6 18

9. Additional Computational Results and Computational Details 19

10. NMR Spectra 46

11. X-RAY Data 80

1. General Experimental Details

Tetrahydrofuran, dichloromethane and toluene were purified by passing through a Pure Solv™ column drying system from Innovative Technology, Inc. Diethyl ether was dried using activated 4 Å molecular sieves and stored under argon. MeOH was purchased dry from Acros Organics. Anhydrous DMSO was purchased from VWR. For convenience, borylation reactions were set up in a nitrogen filled glove box Inert PURELAB PL-HE-2GB. However, performing the borylation reactions under argon atmosphere using flame-dried glassware with standard vacuum-line techniques lead to similar results in terms of yields and regioselectivity.

NMR spectra were acquired on a *Bruker Avance 300 MHz* spectrometer, running at 300, 75, 96 and 282 MHz for ^{1}H, ^{13}C, ^{11}B and ^{19}F, respectively. Chemical shifts (δ) are reported in ppm relative to residual solvent signals ($CDCl_3$, δ_H = 7.26 ppm, δ_C = 77.16 ppm; Toluene-d_8, δ_H = 2.09 ppm, δ_C = 20.40 ppm or D_2O, δ_H = 4.79 ppm, δ_C (MeOH as reference) = 49.5 ppm). For ^{19}F spectra, C_6F_6 is used as internal standard (δ_F = -164.9 ppm). ^{13}C NMR and ^{19}F spectra were acquired on a broad band decoupled mode. The following abbreviations are used to describe peak patterns when appropriate: s (singlet), d (doublet), t (triplet), quint (quintet), m (multiplet), br (broad). Analytical thin layer chromatography (TLC) was performed using pre-coated aluminum-backed plates (Merck Kieselgel 60 F_{254}) and visualized by ultraviolet irradiation and phosphomolybdic acid dip, potassium permanganate dip or cerium ammonium molybdate dip. Flash column chromatography (FC) was performed using silica gel Merck-60 or Florisil® 100-200 mesh from Aldrich. High Resolution Mass Spectrometry (HRMS) were registered in a spectrometer Bruker maXis II™ (Q-TOF) or a *GCT Agilent Technologies 6890 N* using Electronic Impact (EI^+) techniques at 70 eV. Melting points were determined in a Stuart™ melting point SMP3 apparatus in open capillary tubes.

2. Syntheis of Starting Materials

All the starting materials used in this work have been synthetized following the previously reported procedure.[1]

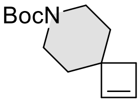

1a

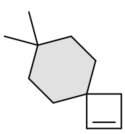

1b

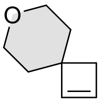

1c

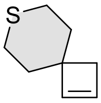

1d

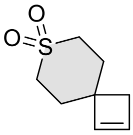

1e

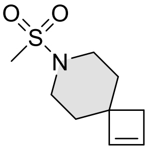

1f

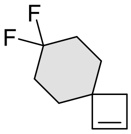

1g

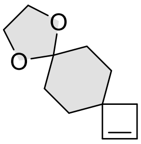

1h

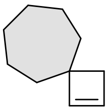

1i

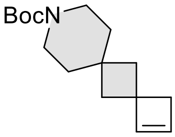

1j

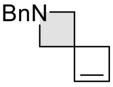

1k

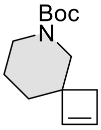

1l

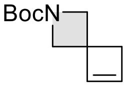

1m

3. Ligand Optimization

An oven-dried vial was charged with CuCl (10 mol%), B_2pin_2 (1.1 equiv), KO*t*-Bu (0.5 equiv) and ligand (11 mol%) in the glove box. Anhydrous THF (0.5 mL/0.2 mmol of **1a**) was added and the mixture was stirred for 15 min. Then, cyclobutene **1a** (1.0 equiv) in THF (1 mL/0.2 mmol of **1a**) was added dropwise followed by methanol (2 equiv). Finally, the reaction mixture was stirred overnight at room temperature. Once the reaction was finished, the resulting solution was filtered through a pad of Celite® (eluted with EtOAc) and concentrated under reduced pressure. The crude mixture was dissolved in THF (0.4 mL/0.2 mmol) and an aqueous solution of NaOH (1M, 0.2 mL/0.2 mmol). Then, the reaction mixture was cooled to 0°C, followed by the addition of a solution of H_2O_2 (30% (w/w), 40 µL/0.2 mmol). The resulting mixture was stirred for 1 hour at room temperature. Once the reaction was finished, the aqueous layer was extracted with ethyl acetate (3 x 10 mL) and the combined organics were dried over $MgSO_4$, filtered, and concentrated under reduced pressured. The combined organic layers were washed with saturated NaCl solution, dried over $MgSO_4$ and finally concentrated under reduced pressure.

The regioselectivity was determited comparing the ^{1}H NMR crude signals of the oxidation products of **2a** and **3a** with those obtained for the cyclobutanol **SI-1** which is an intermediate in the synthesis of the spirocyclobutene **1a**.

NMR data for compound **SI-1**: **^{1}H NMR** (300 MHz, $CDCl_3$): δ 4.40-4.26 (m, 1H), 3.37-3.23 (m, 4H), 2.34-2.22 (m, 3H), 1.57-1.47 (m, 4H), 1.44 /(s, 9H). ***Spectrum***

^{13}C NMR (75 MHz, $CDCl_3$): δ 155.1, 79.5, 63.3, 42.8, 41.0, 39.6, 36.6, 30.4, 28.6. ***Spectrum***

HRMS (ESI$^+$): calculated for $C_{13}H_{23}NNaO_3$ $[M+Na]^+$: 264.1576; found: 264.1571.

---

[1] Nóvoa, L; Trulli, L.; Parra, A.; Tortosa, M. *Angew. Chem. Int. Ed.* **2021**, *60*, 11763.

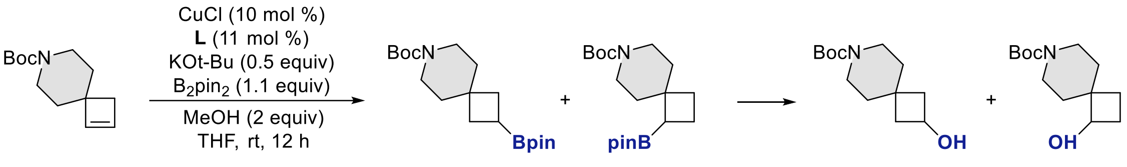

1a **2a** **3a** **SI-1**

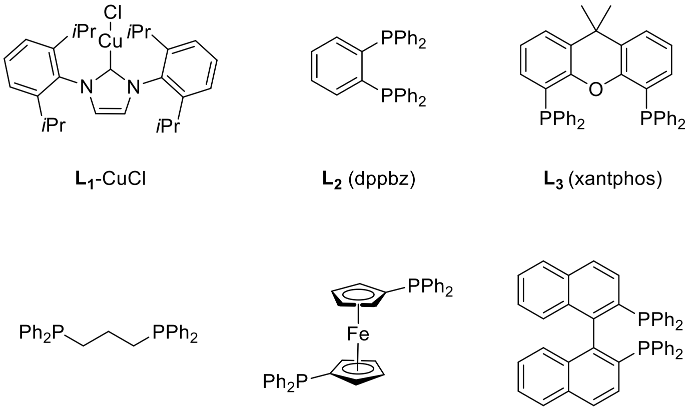

L₁-CuCl **L₂** (dppbz) **L₃** (xantphos) **L₄** (dppp) **L₅** (dppf) **L₆** (binap)

lEntry[a]	L	**2a**:**3a**[b]	Yield (%)[c]
1	L_1	65:35	71
2	L_2	50:50	84
**3**	$\mathbf{L_3}$	**≥ 98:2**	**86**
4	L_4	83:17	58
5	L_5	64:36	79
6	L_6	73:27	69
7	-	60:40	13

[a]Reaction conditions: **1a** (0.1 mmol), B_2pin_2 (0.11 mmol), KO*t*-Bu (0.05 mmol), L (11 mol%), MeOH (0.2 mmol), THF (0.2 M). [b]Determined by ^{1}H-NMR. [c]Isolated yields.

4. General Procedure for the Regioselective Monoboration of Spirocyclobutenes

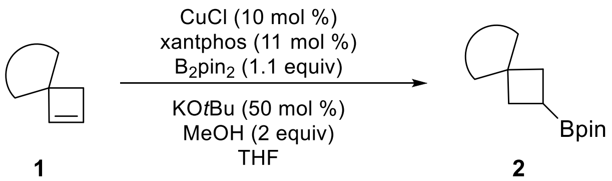

1 **2**

An oven-dried vial was charged with CuCl (10 mol%), B_2pin_2 (1.1 equiv), KO*t*-Bu (0.5 equiv) and xantphos (11 mol%) in the glove box. Anhydrous THF (0.5 mL/0.2 mmol of **1**) was added and the mixture was stirred for 15 min. Then, the corresponding cyclobutene **1** (1.0 equiv) in THF (1 mL/0.2 mmol of **1**) was added dropwise followed by methanol (2 equiv). Finally, the reaction

mixture was stirred overnight at room temperature. Once the reaction was finished, the resulting solution was filtered through a pad of Celite® (eluted with EtOAc) and concentrated under reduced pressure. The crude product was purified by flash column chromatography on silica gel or Florisil® to afford cyclobutylboronate **2**.

***tert*-Butyl 2-(4,4,5,5-tetramethyl-1,3,2-dioxaborolan-2-yl)-7-azaspiro[3.5]nonane-7 carboxylate, 2a**

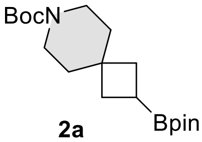

2a

From **1a** (44.7 mg, 0.2 mmol) following the general procedure described above, compound **2a** (60.5 mg, 0.172 mmol) was obtained in 86% yield as a white solid, after purification by flash column chromatography (SiO_2; cyclohexane/EtOAc 90:10).

mp = 51-53 °C.

^{1}H NMR (300 MHz, $CDCl_3$): δ 3.33-3.20 (m, 4H), 1.91-1.73 (m, 5H), 1.56-1.50 (m, 2H), 1.49-1.40 (m, 11H), 1.25-1.20 (m, 12H). Spectrum

^{13}C NMR (75 MHz, $CDCl_3$): δ 155.2, 83.2, 79.2, 40.7 (br), 38.1, 37.3, 36.7, 33.1, 28.6, 24.9, 10.1 (br). Spectrum

^{11}B NMR (96 MHz, $CDCl_3$): δ 33.5. Spectrum

HRMS (ESI$^+$): calculated for $C_{19}H_{34}BNNaO_4$ $[M+Na]^+$: 374.2479; found: 374.2469.

2-(7,7-Dimethylspiro[3.5]nonan-2-yl)-4,4,5,5-tetramethyl-1,3,2-dioxaborolane, 2b

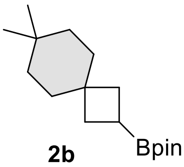

2b

From **1b** (30.1 mg, 0.2 mmol) following the general procedure described above, compound **2b** (41.9 mg, 0.151 mmol) was obtained in 75% yield as colorless oil, after purification by flash column chromatography (SiO_2; cyclohexane/EtOAc 90:10).

^{1}H NMR (300 MHz, $CDCl_3$): δ 1.86-1.66 (m, 5H), 1.54-1.46 (m, 2H), 1.45-1.37 (m, 2H), 1.23 (s, 12H), 1.20-1.10 (m, 4H), 0.83 (s, 6H). Spectrum

^{13}C NMR (75 MHz, $CDCl_3$): δ 83.0, 38.8, 35.7, 35.6, 35.2, 33.8, 33.6, 29.7, 28.5 (br), 24.9, 10.4 (br). Spectrum

^{11}B NMR (96 MHz, $CDCl_3$): δ 34.0. Spectrum

HRMS (EI$^+$): calculated for $C_{17}H_{31}BO_2$ $[M]^+$: 278.2417; found: 278.2422.

4,4,5,5-Tetramethyl-2-(7-oxaspiro[3.5]nonan-2-yl)-1,3,2-dioxaborolane, 2c

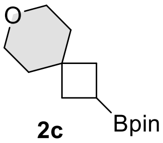

2c

From **1c** (24.8 mg, 0.2 mmol) following the general procedure described above, compound **2c** (39.2 mg, 0.155 mmol) was obtained in 78% yield as a pale yellow oil, after purification by flash column chromatography (Florisil®; cyclohexane/EtOAc 90:10).

^{1}H NMR (300 MHz, $CDCl_3$): δ 3.55 (dt, J = 13.0, 5.3 Hz, 4H), 1.95-1.76 (m, 5H), 1.65-1.58 (m, 2H), 1.57-1.50 (m, 2H), 1.23 (s, 12H). Spectrum

^{13}C NMR (75 MHz, $CDCl_3$): δ 83.1, 64.8, 64.6, 39.1, 37.9, 36.4, 33.7, 24.8. [note: the carbon attached to boron was not observed due to quadrupole broadening caused by the ^{11}B nucleus]. Spectrum

^{11}B NMR (96 MHz, $CDCl_3$): δ 34.4. Spectrum

HRMS (EI$^+$): calculated for $C_{14}H_{25}BO_3$ $[M]^+$: 252.1897; found: 252.1909.

4,4,5,5-Tetramethyl-2-(7-thiaspiro[3.5]nonan-2-yl)-1,3,2-dioxaborolane, 2d

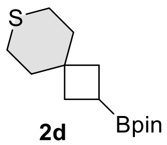

2d

From **1d** (28.0 mg, 0.2 mmol) following the general procedure described above, compound **2d** (35.2 mg, 0.131 mmol) was obtained in 66% yield as yellow oil, after purification by flash column chromatography (SiO_2; cyclohexane/EtOAc 90:10).

^{1}H NMR (300 MHz, $CDCl_3$): δ 2.59-2.43 (m, 4H), 1.89-1.66 (m, 9H) 1.24 (s, 12H). *Spectrum*

^{13}C NMR (75 MHz, $CDCl_3$): δ 83.2, 39.8, 38.1, 37.9, 33.7, 25.2, 25.0, 24.9. [note: the carbon attached to boron was not observed due to quadrupole broadening caused by the ^{11}B nucleus]. *Spectrum*

^{11}B NMR (96 MHz, $CDCl_3$): δ 33.8. *Spectrum*

HRMS (EI$^+$): calculated for $C_{14}H_{25}BO_2S$ $[M]^+$: 268.1668; found: 268.1678.

2-(4,4,5,5-Tetramethyl-1,3,2-dioxaborolan-2-yl)-7-thiaspiro[3.5]nonane 7,7-dioxide, 2e

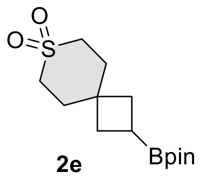

2e

From **1e** (34.4 mg, 0.2 mmol) following the general procedure described above, compound **2e** (39.5 mg, 0.132 mmol) was obtained in 66% yield as a white solid, after purification by flash column chromatography (SiO_2; cyclohexane/EtOAc 70:30).

mp = 113-115 °C.

^{1}H NMR (300 MHz, $CDCl_3$): δ 2.99-2.83 (m, 4H), 2.21-2.06 (m, 4H), 1.99-1.82 (m, 5H), 1.24 (s, 12H). *Spectrum*

^{13}C NMR (75 MHz, $CDCl_3$): δ 83.5, 48.1, 48.0, 36.6, 35.7, 34.3, 32.0, 24.9. [note: the carbon attached to boron was not observed due to quadrupole broadening caused by the ^{11}B nucleus]. *Spectrum*

HRMS (EI$^+$): calculated for $C_{14}H_{25}BO_4S$ $[M]^+$: 300.1567; found: 300.1573.

7-(Methylsulfonyl)-2-(4,4,5,5-tetramethyl-1,3,2-dioxaborolan-2-yl)-7-azaspiro[3.5]nonane, 2f

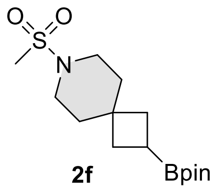

2f

From **1f** (40.3 mg, 0.2 mmol) following the general procedure described above, compound **2f** (51.4 mg, 0.156 mmol) was obtained in 78% yield as a white solid, after purification by flash column chromatography (SiO_2; cyclohexane/EtOAc 70:30).

mp = 87-89 °C.

^{1}H NMR (300 MHz, $CDCl_3$): δ 3.11 (dt, J = 11.6, 5.5 Hz, 1H), 2.74 (s, 3H), 1.95-1.76 (m, 5H), 1.75-1.61 (m, 4H), 1.25 (s, 12H). *Spectrum*

^{13}C NMR (75 MHz, $CDCl_3$): δ 83.3, 42.9, 42.8, 37.4, 36.5, 36.3, 34.6, 32.8, 24.8. [note: the carbon attached to boron was not observed due to quadrupole broadening caused by the ^{11}B nucleus]. *Spectrum*

^{11}B NMR (96 MHz, $CDCl_3$): δ 33.8. *Spectrum*

HRMS (EI$^+$): calculated for $C_{15}H_{28}BNO_4S$ $[M]^+$: 329.1832; found: 329.1841.

2-(7,7-Difluorospiro[3.5]nonan-2-yl)-4,4,5,5-tetramethyl-1,3,2-dioxaborolane, 2g

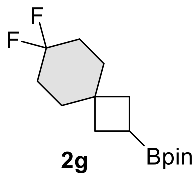

2g

From **1g** (31.6 mg, 0.2 mmol) following the general procedure described above, compound **2g** (47.0 mg, 0.164 mmol) was obtained in 82% yield as a white solid, after purification by flash column chromatography (SiO_2; cyclohexane/EtOAc 90:10).

mp = 52-54 °C.

^{1}H NMR (300 MHz, $CDCl_3$): δ 1.92-1.66 (m, 11H), 1.66-1.58 (m, 2H), 1.24 (s, 12H). *Spectrum*

^{13}**C NMR** (75 MHz, $CDCl_3$): δ 123.7 (t, *J* = 240.5 Hz), 83.2, 37.5, 34.8 (t, *J* = 4.8 Hz), 33.4 (t, *J* = 4.9 Hz), 32.6, 30.5 (t, *J* = 23.9 Hz), 30.4 (t, *J* = 23.9 Hz), 24.9, 10.2 (br). *Spectrum*
^{19}**F NMR** (282 MHz, $CDCl_3$): δ −100.5. *Spectrum*
^{11}**B NMR** (96 MHz, $CDCl_3$): δ 34.1. *Spectrum*
HRMS (EI$^+$): calculated for $C_{15}H_{25}BF_2O_2$ $[M]^+$: 286.1916; found: 286.1917.

2-(8,11-Dioxadispiro[3.2.4^7.2^4]tridecan-2-yl)-4,4,5,5-tetramethyl-1,3,2-dioxaborolane, 2h

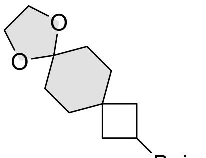

2h Bpin

From **1h** (36.1 mg, 0.2 mmol) following the general procedure described above, compound **2h** (55.1 mg, 0.179 mmol) was obtained in 89% yield as a colorless oil, after purification by flash column chromatography (Florisil®; hexane).
^{1}**H NMR** (300 MHz, $CDCl_3$): δ 3.90 (s, 4H), 1.89-1.73 (m, 5H), 1.71-1.63 (m, 2H), 1.61-1.48 (m, 6H), 1.23 (s, 12H). *Spectrum*
^{13}**C NMR** (75 MHz, $CDCl_3$): δ 109.0, 83.1, 64.3, 38.0, 36.2, 34.5, 33.1, 31.3, 31.2, 24.9. [note: the carbon attached to boron was not observed due to quadrupole broadening caused by the ^{11}B nucleus]. *Spectrum*
^{11}**B NMR** (96 MHz, $CDCl_3$): δ 33.9. *Spectrum*
HRMS (EI$^+$): calculated for $C_{17}H_{29}BO_4$ $[M]^+$: 308.2159; found: 308.2144.

4,4,5,5-Tetramethyl-2-(spiro[3.6]decan-2-yl)-1,3,2-dioxaborolane, 2i

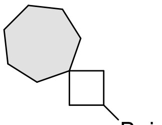

2i Bpin

From **1i** (27.2 mg, 0.2 mmol) following the general procedure described above, compound **2i** (38.0 mg, 0.144 mmol) was obtained in 72% yield as a colorless oil, after purification by flash column chromatography (SiO_2; cyclohexane/EtOAc 90:10).
^{1}**H NMR** (300 MHz, $CDCl_3$): δ 1.89-1.71 (m, 5H), 1.68-1.62 (m, 2H), 1.60-1.54 (m, 2H), 1.52-1.35 (m, 8H), 1.24 (s, 12H). *Spectrum*
^{13}**C NMR** (75 MHz, $CDCl_3$): δ 83.0, 42.1 (x2), 40.9, 35.5, 28.0, 27.9 (x2), 24.9, 23.1, 22.6. [note: the carbon attached to boron was not observed due to quadrupole broadening caused by the ^{11}B nucleus]. *Spectrum*
HRMS (EI$^+$): calculated for $C_{16}H_{29}BO_2$ $[M]^+$: 264.2261; found: 264.2250.

***tert*-Butyl 2-(4,4,5,5-tetramethyl-1,3,2-dioxaborolan-2-yl)-9-azadispiro[3.1.5^6.1^4]dodecane-9-carboxylate, 2j**

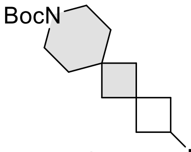

2j Bpin

From **1j** (52.7 mg, 0.2 mmol) following the general procedure described above, compound **2j** (67.1 mg, 0.171 mmol) was obtained in 86% yield as a white solid, after purification by flash column chromatography (SiO_2; cyclohexane/EtOAc 90:10).
mp = 62-65 °C.
^{1}**H NMR** (300 MHz, $CDCl_3$): δ 3.30-3.21 (m, 4H), 2.13-1.95 (m, 4H), 1.82 (s, 2H), 1.79-1.68 (m, 3H), 1.45-1.37 (m, 13H), 1.22 (s, 12H). *Spectrum*
^{13}**C NMR** (75 MHz, $CDCl_3$): δ 155.1, 83.1, 79.2, 45.5, 43.3, 40.9 (br), 38.3, 38.1, 37.6, 32.7, 28.6, 24.9. [note: the carbon attached to boron was not observed due to quadrupole broadening caused by the ^{11}B nucleus]. *Spectrum*
^{11}**B NMR** (96 MHz, $CDCl_3$): δ 33.7. *Spectrum*
HRMS (EI$^+$): calculated for $C_{22}H_{38}BNO_4$ $[M]^+$: 391.2894; found: 391.2901.

tert-Butyl 6-(4,4,5,5-tetramethyl-1,3,2-dioxaborolan-2-yl)-2-azaspiro[3.3]heptane-2-carboxylate, 2k

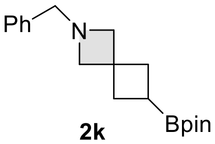

2k

From **1k** (37.1 mg, 0.2 mmol) following the general procedure described above, compound **2k** (38.5 mg, 0.123 mmol) was obtained in 61% yield as a yellow oil, after purification by flash column chromatography (SiO_2 deactivated with Et_3N, DCM/MeOH 95:5).

^{1}H NMR (300 MHz, $CDCl_3$): δ 7.33-7.18 (m, 5H), 3.55 (s, 2H), 3.21 (s, 2H), 3.18 (s, 2H), 2.27-2.08 (m, 4H), 1.77-1.64 (m, 1H), 1.22 (s, 12H). *Spectrum*

^{13}C NMR (75 MHz, $CDCl_3$): δ 138.7, 128.6, 128.3, 126.9, 83.1, 67.3, 66.9, 64.1, 39.0, 34.6, 24.9. [note: the carbon attached to boron was not observed due to quadrupole broadening caused by the ^{11}B nucleus]. *Spectrum*

^{11}B NMR (96 MHz, $CDCl_3$): δ 33.4. *Spectrum*

HRMS (EI$^+$): calculated for $C_{19}H_{28}BNO_2$ $[M]^+$: 313.2213; found: 313.2224.

tert-Butyl 2-(4,4,5,5-tetramethyl-1,3,2-dioxaborolan-2-yl)-6-azaspiro[3.5]nonane-6-carboxylate, 2l

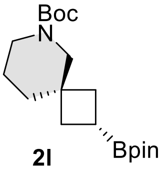

2l

From **1l** (44.7 mg, 0.2 mmol) following the general procedure described above, compound **2l** (58.1 mg, 0.165 mmol) was obtained in 83% yield as a white solid, after purification by flash column chromatography (SiO_2; cyclohexane/EtOAc 90:10).

mp = 75-77 °C.

^{1}H NMR (300 MHz, Toluene-d_8, 373K): δ 3.33-3.29 (s, 2H), 3.24-3.18 (m, 2H), 1.95-1.87 (m, 2H), 1.82-1.74 (m, 2H), 1.43-1.37 (m, 11H), 1.28-123 (m, 2H), 1.06 (s, 12H). *Spectrum*

^{13}C NMR (75 MHz, Toluene-d_8, 373K): δ 155.2, 83.2, 78.7, 53.8, 44.6, 39.0, 38.1, 32.3, 28.8, 25.0, 22.5. [note: the carbon attached to boron was not observed due to quadrupole broadening caused by the ^{11}B nucleus]. *Spectrum*

HRMS (EI$^+$): calculated for $C_{19}H_{34}BNO_4$ $[M]^+$: 351.2581; found: 351.2587.

tert-Butyl 6-(4,4,5,5-tetramethyl-1,3,2-dioxaborolan-2-yl)-2-azaspiro[3.3]heptane-2-carboxylate, 2m

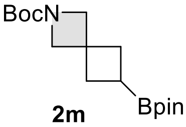

2m

From **1m** (39.1 mg, 0.2 mmol) following the general procedure described above, compound **2m** (56.4 mg, 0.174 mmol) was obtained in 87% yield as a white solid, after purification by flash column chromatography (Florisil®; cyclohexane/EtOAc 90:10).

mp = 69-71°C.

^{1}H NMR (300 MHz, $CDCl_3$): δ 3.84 (s, 2H), 3.82 (s, 2H), 2.28-2.07 (m, 4H), 1.67 (tt, J = 9.6, 7.2 Hz, 1H), 1.40 (s, 9H), 1.21 (s, 12H). *Spectrum*

^{13}C NMR (75 MHz, $CDCl_3$): δ 156.4, 83.3, 79.2, 61.9, 61.7, 37.8, 34.5, 28.5, 24.8. [note: the carbon attached to boron was not observed due to quadrupole broadening caused by the ^{11}B nucleus]. *Spectrum*

HRMS (ESI$^+$): calculated for $C_{17}H_{30}BNNaO_4$ $[M+Na]^+$: 346.2166; found: 346.2154.

5. Gram Scale Experiments

5.1 Synthesis of *tert*-Butyl 6-(tosyloxy)-2-azaspiro[3.3]heptane-2-carboxylate, SI-3

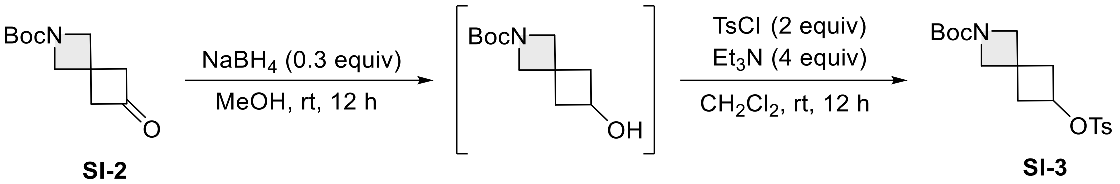

To a solution of *tert*-butyl 2-oxo-7-azaspiro[3.5]nonane-7-carboxylate **SI-2** (6.0 g, 28.4 mmol) in MeOH (1.7 mL/mmol) at 0 °C, $NaBH_4$ (0.3 equiv) was added portionwise. The resulting mixture was stirred at room temperature for 12 h. Then, the solvent was evaporated and the residue was re-dissolved in EtOAc (3 mL/mmol) and water (3 mL/mmol). The layers were separated and the aqueous phase was extracted with EtOAc (3x). The organic phases were washed with brine, dried over $MgSO_4$ and the solvent evaporated under reduced pressure. The residue was used in the next step without further purification.

To a solution of the cyclobutanol and triethylamine (4 equiv) in CH_2Cl_2 (1.5 mL/mmol), a solution of TsCl (2.0 equiv) in CH_2Cl_2 (0.6 mL/mmol) was added dropwise at 0 °C.

After being stirred for 12 h at room temperature, water (2.5 mL/mmol of alcohol) and CH_2Cl_2 (2.5 mL/mmol of alcohol) were added to the reaction mixture. Then, the layers were separated and the aqueous phase was extracted with CH_2Cl_2 (3x). The combined organic phases were dried ($MgSO_4$), filtered and the solvent evaporated under reduced pressure. The residue was purified by flash column chromatography (SiO_2; cyclohexane/EtOAc 80:20) to afford the desired product **SI-3** (9.42 g, 25.6 mmol) in 91% as a white solid. The spectral data matched with those previously reported.[1]

5.2 Synthesis of tert-Butyl 2-azaspiro[3.3]hept-5-ene-2-carboxylate, 1m

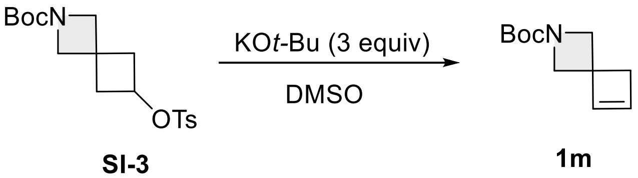

In an oven-dried flask, KO*t*-Bu (3 equiv) was placed and anhydrous DMSO (2 mL/mmol) was added, under an argon atmosphere, to give a colorless solution. Then, a solution of tosylate **SI-3** (9.42 g, 25.6 mmol) in DMSO (2 mL/mmol) was added very slowly. After being stirred 4 h at room temperature, the crude was filtered through a pad of silica gel (pentane/Et_2O 90:10). The solvent was removed under reduced pressure to afford the desired spirocyclobutene **1m** in 78% yield as a colorless oil. The spectral data matched with those previously reported.[1]

5.3 Synthesis of *tert*-Butyl 6-(4,4,5,5-tetramethyl-1,3,2-dioxaborolan-2-yl)-2-azaspiro[3.3]heptane-2-carboxylate, 2m

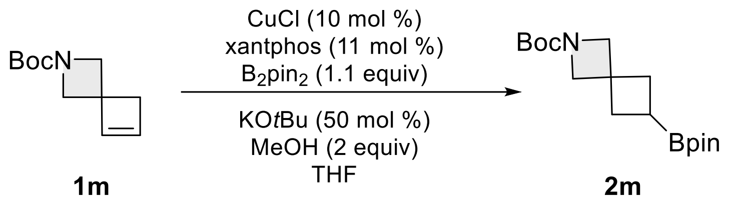

An oven-dried vial was charged with CuCl (10 mol%), B_2pin_2 (1.1 equiv), KO*t*-Bu (0.5 equiv) and xantphos (11 mol%) in the glove box. Anhydrous THF (0.5 mL/0.2 mmol of **1m**) was added and the mixture was stirred for 15 min. Then, cyclobutene **1m** (1 g, 5.1 mmol) in THF (1 mL/0.2 mmol of **1m**) was added dropwise followed by methanol (2 equiv). Finally, the reaction mixture was stirred overnight at room temperature. Once the reaction was finished, the resulting solution was filtered through a pad of Celite® (eluted with EtOAc) and concentrated under reduced pressure. The crude product was purified by flash column chromatography (Florisil®; cyclohexane/EtOAc 90:10) to afford cyclobutylboronate **2m** (1.49 g, 4.6 mmol) in a 90% yield as a white solid.

6. Bioisosteric Replacement: Synthesis of Donepezil Derivative

6.1 Synthesis of *tert*-butyl 6-(hydroxymethyl)-2-azaspiro[3.3]heptane-2-carboxylate, SI-4

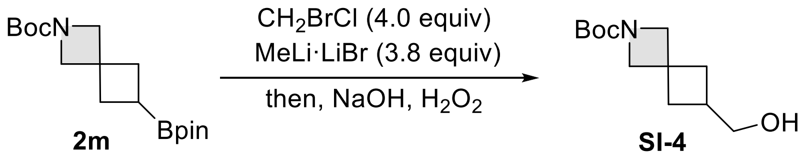

CH_2BrCl (367 µL, 5.64 mmol, 4.0 equiv) was added to a solution of boronate **2m** (458 mg, 1.41 mmol) in anhydrous THF (3 mL/mmol), under an argon atmosphere. The solution was cooled to −78 °C and MeLi·LiBr (3.6 mL of a 1.5 M solution in Et_2O, 5.36 mmol, 3.8 equiv) was added dropwise. The mixture was warmed to room temperature and stirred overnight. Then, the mixture was transferred to a separatory funnel, water was added and the phases were separated. The aqueous phase was extracted with EtOAc (3x), the combined organic layers dried ($MgSO_4$) and the solvent removed under reduced pressure.

The reaction mixture was redissolved in THF (2 mL/mmol) and cooled to 0 °C. Then, 1M aqueous solution of NaOH (1 mL/mmol) and H_2O_2 (0.2 mL/mmol) were successively added dropwise. The mixture was stirred at room temperature for 1h. After this time, water was added and the phases were separated. The aqueous phase was extracted with EtOAc (3x), the combined organic layers dried ($MgSO_4$) and the solvent removed *in vacuo*. The residue was purified by flash column chromatography (SiO_2, $CHCl_3$/*i*-PrOH 95:5, flash column chromatography repeated twice to separate from traces of oxidation product of **2j**) to afford alcohol **SI-4** (265 mg, 1.17 mmol) in 83% yield as a colourless oil.

^{1}H NMR (300 MHz, $CDCl_3$): δ 3.87 (s, 2H), 3.77 (s, 2H), 3.48 (d, *J* = 6.2 Hz, 2H), 2.49 (br, 1H), 2.37-2.24 (m, 1H), 2.24-2.13 (m, 2H), 1.95-1.84 (m, 2H), 1.37 (s, 9H). Spectrum
^{13}C NMR (75 MHz, $CDCl_3$): δ 156.4, 79.4, 66.4, 62.2 (br), 61.5 (br), 35.4, 34.4, 31.4, 28.4. Spectrum
HRMS (ESI$^+$): calculated for $C_{12}H_{21}NNaO_3$ $[M+Na]^+$: 250.1419; found: 250.1411.

6.2 Synthesis of *tert*-Butyl 6-formyl-2-azaspiro[3.3]heptane-2-carboxylate, 4

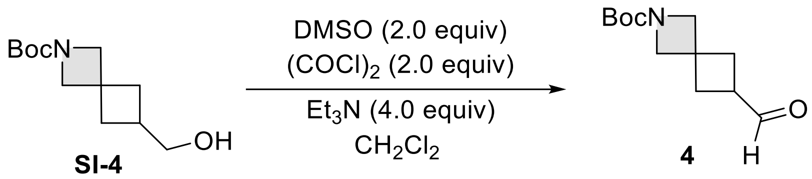

A solution of DMSO (165 µL, 2.33 mmol, 2 equiv) in CH_2Cl_2 (1 mL) was added to a solution of oxalylchloride (200 µL, 2.33 mmol, 2 equiv) in CH_2Cl_2 (4 mL) at –78 °C, under an argon atmosphere. The mixture was stirred for 15 min at –78 °C and a solution of alcohol **SI-4** (265 mg, 1.17 mmol) in CH_2Cl_2 (1 mL) was added dropwise. After stirring for 30 min, Et_3N (652 µL, 4.68 mmol, 4 equiv) was added to the mixture and then the reaction was allowed to warm to 0 °C. The reaction was stirred for further 45 min. Then, aqueous $NaHCO_3$ was added and the mixture was extracted with CH_2Cl_2 (3x), dried ($MgSO_4$) and concentrated under reduced pressure. The residue was purified by flash column chromatography (SiO_2; hexane/EtOAc 80:20) to afford the corresponding aldehyde **4** (215 mg, 0.95 mmol) in 82% yield as a colourless oil.

^{1}H NMR (300 MHz, $CDCl_3$): δ 9.71 (s, 1H), 3.93 (s, 2H), 3.81 (s, 2H), 3.15-3.01 (m, 1H), 2.48-2.30 (m, 4H), 1.41 (s, 9H). *Spectrum*

^{13}C NMR (75 MHz, $CDCl_3$): δ 201.3, 156.0, 79.3, 61.4 (br), 39.9, 34.6, 32.9, 28.3. *Spectrum*

HRMS (EI$^+$): calculated for $C_{12}H_{19}NO_3$ $[M]^+$: 225.1365; found: 225.1359.

6.3 Synthesis of *tert*-butyl 6-((5,6-dimethoxy-1-oxo-2,3-dihydro-1*H*-inden-2-yl)methyl)-2-azaspiro[3.3]heptane-2-carboxylate, 5

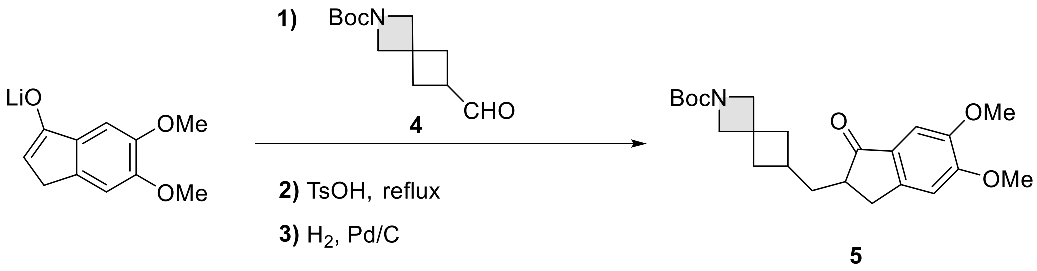

To a solution of *N*,*N*-diisopropylamine (112 µL, 0.80 mmol, 1.1 equiv) of in 3.1 mL of THF at –78 °C, under an argon atmosphere, was added dropwise *n*-butyllithium (0.5 mL, 1.6 M in hexane, 1.1 equiv). The reaction mixture was allowed to stir at 25 °C for 30 minutes. Then, a solution of 5,6-dimethoxy-2,3-dihydro-1*H*-inden-1-one (169 mg, 0.88 mmol, 1.2 equiv) in 1.5 mL of THF at –78 °C was added dropwise to the lithium *N*,*N*-diisopropylamide solution and the mixture was stirred for 1.5 h. After this time, a solution of aldehyde **4** (162.5 mg, 0.72 mmol, 1 equiv) was added to the prepared enolate and the mixture was stirred an additional 1 h. Upon completion by TLC, the reaction mixture was quenched with a saturated solution of NH_4Cl (4 mL), extracted with EtOAc (3x), washed with brine, dried over $MgSO_4$ and the solvent removed under reduced pressure.

In order to carry out the dehydration, the aldol product was dissolved in toluene (0.7 M) and a catalytic amount of p-TsOH·H_2O (69.4 mg, 0.365 mmol, 0.5 equiv) was added. The reaction was refluxed (oil bath) for 3 h. Once the reaction was finished, it was diluted with EtOAc (5 mL), washed with a saturated solution of $NaHCO_3$, dried over $MgSO_4$, and the solvent removed under reduced pressure. The crude was used in the next step without further purification.

Pd/C 10% (47 mg, 0.044 mmol, 6 mol%) was added to the crude containing the α,β-unsaturated ketone in THF (15 mL) and H_2 (balloon) was bubbled through the solution. The mixture was stirred under hydrogen atmosphere for 0.5 h at room temperature. Once it was finished by TLC, the reaction mixture was filtered through Celite® and washed with EtOAc (20 mL). The solvent was removed under reduced pressure and the residue purified by flash column chromatography (SiO_2, hexane/EtOAc/Et_3N 50:49:1) to afford product **5** (81.0 mg, 0.202 mmol) in a 28% overall yield (three steps) as pale brown oil.

^{1}H NMR (300 MHz, $CDCl_3$): δ 7.12 (s, 1H), 6.82 (s, 1H), 3.92 (s, 3H), 3.89 (s, 2H), 3.87 (s, 3H), 3.76 (s, 2H), 3.14 (dd, J = 16.9, 7.5 Hz, 1H), 2.64 (dd, J = 16.9, 3.4 Hz, 1H), 2.56-2.47 (m, 1H), 2.29-2.20 (m, 3H), 1.99-1.91 (m, 1H), 1.86-1.77 (m, 2H), 1.55-1.46 (m, 1H), 1.39 (s, 9H). Spectrum
^{13}C NMR (75 MHz, $CDCl_3$): δ 207.3, 156.4, 155.6, 149.6, 148.8, 129.4, 107.5, 104.5, 79.2, 62.5, 60.9, 56.3, 56.2, 46.2, 39.6, 39.1, 38.5, 34.4, 32.7, 28.5, 28.4. Spectrum
HRMS (ESI$^+$): calculated for $C_{23}H_{31}NNaO_5$ $[M+Na]^+$: 424.2100; found: 424.2087.

6.4 Synthesis of 2-((2-benzyl-2-azaspiro[3.3]heptan-6-yl)methyl)-5,6-dimethoxy-2,3-dihydro-1*H*-inden-1-one, 6

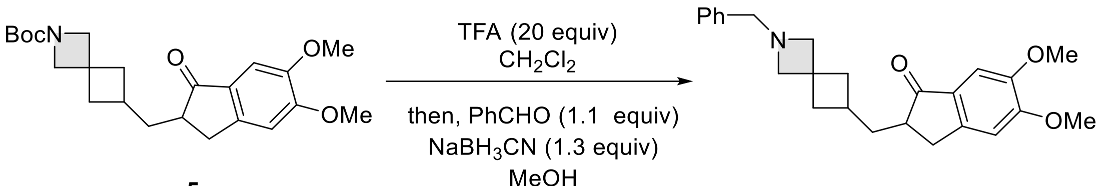

5 **6**

In an oven-dried flask, **5** (81.0 mg, 0.202 mmol, 1 equiv) was dissolved in CH_2Cl_2 (5 mL/mmol), under an argon atmosphere, and TFA (309 µL, 4.04 mmol, 20 equiv) was added dropwise to the solution at 0 °C. After being stirred at room temperature for 1 h, the reaction mixture was washed with a solution of saturated Na_2CO_3. The organic phase was washed with brine, dried over $MgSO_4$ and the solvent evaporated under reduced pressure. The crude was used in the next step without further purification.

The crude mixture was dissolved in methanol (5 mL/mmol) and benzaldehyde (23 µL, 0.222 mmol, 1.1 equiv) was added. The solution was cooled to 0 °C and $NaCNBH_3$ (16.5 mg, 0.263 mmol, 1.3 equiv) was added portionwise. The resulting mixture was stirred at room temperature for 16 h. After this time, the solvent was removed under reduced pressure. Saturated aqueous $NaHCO_3$ (10 mL/mmol) and EtOAc (10 mL/mmol) were added, the phases separated and the aqueous layer was extracted with EtOAc (20 mL × 3). Finally, the combined organic phases were dried over $MgSO_4$, filtrated, and the solvent was removed under reduced pressure. The reaction crude was purified by flash column chromatography (SiO_2; CH_2Cl_2/Et_3N 99:1 to CH_2Cl_2/MeOH/Et_3N 95:4:1) to afford desired product **6** (44.9 mg, 0.115 mmol) in 57% yield as pale brown oil.
^{1}H NMR (300 MHz, $CDCl_3$): δ 7.30-7.15 (m, 5H), 7.08 (s, 1H), 6.77 (s, 1H), 3.88 (s, 3H), 3.82 (s, 3H), 3.55 (s, 2H), 3.26 (s, 2H), 3.16 (s, 2H), 3.08 (dd, J = 16.9, 7.5 Hz, 1H), 2.58 (dd, J = 16.9, 3.5 Hz, 1H), 2.52-2.42 (m, 1H), 2.28-2.14 (m, 3H), 1.94-1.85 (m, 1H), 1.83-1.71 (m, 2H), 1.50-1.37 (m, 1H). Spectrum
^{13}C NMR (75 MHz, $CDCl_3$): δ 207.5, 155.6, 149.6, 149.0, 137.3, 129.5, 128.8, 128.5, 127.4, 107.5, 104.5, 67.2, 66.0, 63.4, 56.3, 56.2, 46.4, 39.6, 38.9, 38.7, 35.6, 32.7, 29.0. Spectrum
HRMS (EI$^+$): calculated for $C_{25}H_{29}NO_3$ $[M]^+$: 391.2147; found: 391.2142.

7. Functionalization of Monoborylated Spirocyles

7.1 Vinylation

Synthesis of *tert*-butyl 2-vinyl-7-azaspiro[3.5]nonane-7-carboxylate, 7

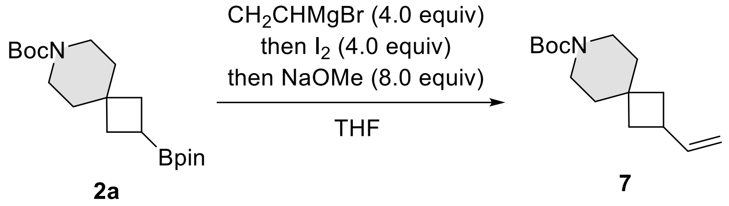

An oven-dried vial was charged with boronic ester **2a** (70.3 mg, 0.2 mmol, 1 equiv) and was dissolved in anhydrous THF (1 mL).[2] Then, vinyl magnesium bromide (0.8 mmol, 1.0 M in THF, 4.0 equiv) was added dropwise at a 0 °C, and the resulting solution was stirred for 30 min at room temperature. After cooling the reaction mixture at –78 °C (dry ice/acetone), a solution of iodine (203.0 mg, 0.8 mmol, 4.0 equiv) in anhydrous THF (1.7 mL) was added dropwise followed by stirring for 20 min. After this time, the solution was warmed at 0 °C, and a suspension of NaOMe (86.4 mg, 1.6 mmol, 8.0 equiv) in methanol (1 mL) was added in a single portion and was stirred for a further 30 min at that temperature. Then, a saturated aqueous solution of $Na_2S_2O_3$ (7 mL) was added, followed by DCM (10 mL). The phases were separated and the aqueous phase was extracted with DCM (2 × 10 mL). The combined organic phases were dried ($MgSO_4$) and concentrated under reduced pressure. The crude residue was purified by flash column chromatography (SiO_2; cyclohexane/EtOAc 90:10 to 80:20) to afford the desired alkene **7** (25.2 mg, 0.100 mmol) in 50% yield as colorless oil.

^{1}H NMR (300 MHz, $CDCl_3$): δ 5.93 (ddd, *J* = 16.9, 10.2, 6.5 Hz, 1H), 4.99-4.86 (m, 2H), 3.38-3.31 (m, 2H), 3.30-3.22 (m, 2H), 2.98-2.91 (m, 1H), 2.06-1.96 (m, 2H), 1.69-1.55 (m, 4H), 1.44 (s, 11H).
Spectrum

^{13}C NMR (75 MHz, $CDCl_3$): δ 155.1, 143.5, 112.4, 79.3, 40.9 (br), 39.4, 37.6, 36.3, 34.2, 32.2, 28.6.
Spectrum

HRMS (ESI$^+$): calculated for $C_{15}H_{25}NNaO_2$ $[M+Na]^+$: 274.1783; found: 274.1774.

7.2 Arylation

Synthesis of 4-(7,7-dimethylspiro[3.5]nonan-2-yl)-3-fluoropyridine, 8

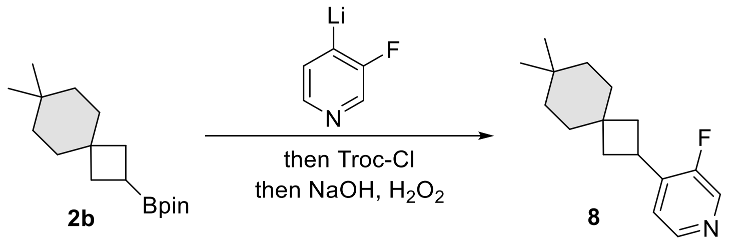

n-Butyllithium (0.6 mmol, 2.0 equiv) was added dropwise, under an argon atmosphere, to a solution of *N*,*N*-diisopropylamine (84 µL, 0.6 mmol, 2.0 equiv) in anhydrous THF (1 mL) at –78 °C and the mixture was allowed to react for 1 h.[9] The solution was warmed to –60 °C and a solution of 3-fluoropyridine (58.3 mg, 0.6 mmol, 2.0 equiv) in THF (0.5 mL) was added and the solution

[2] Fawcett, A.; Biberger, T.; Aggarwal, V. K. *Nature Chem.* **2019**, *11*, 117–122.

was stirred for 30 min. The mixture was cooled back to –78 °C and a solution of **2b** (83.5 mg, 0.3 mmol) in THF (1 mL) was added dropwise and stirred for 30 min. Then, the solution was warmed to 0 °C and stirred for a further 30 min. The solution was cooled back to –78 °C and 2,2,2-trichloroethyl chloroformate (165 µL, 1.2 mmol, 4.0 equiv) was added dropwise and the resulting mixture was stirred at room temperature overnight. The mixture was transferred to a separating funnel and diluted with Et_2O (10 mL) and saturated aqueous solution of $NaHCO_3$ (10 mL). The layers were separated, and the aqueous phase was extracted with Et_2O (3 × 10 mL). The combined organic phases were dried ($MgSO_4$) and concentrated *in vacuo*. The residue was dissolved in THF (3 mL) and cooled to 0 °C. NaOH (3 M aqueous solution, 1.5 mL) and H_2O_2 (30 % aqueous solution, 1.5 mL) were slowly added. The mixture was warmed to room temperature and stirred for 14 h. Then, the mixture diluted with Et_2O (5 mL) and then HCl (1 M aqueous solution) was added to acidify the aqueous phase, which was extracted with Et_2O (3 × 5 mL). The aqueous phase was then neutralized with $NaHCO_3$ and extracted with Et_2O (3 × 10 mL). The combined organic phases were dried ($MgSO_4$) and concentrated under reduced pressure. The crude residue was purified by flash column chromatography (SiO_2; hexane/EtOAc 95:5) to afford the desired compound **8** (39.8 mg, 0.161 mmol) in 54% yield as colourless oil.

^{1}H NMR (300 MHz, $CDCl_3$): δ 8.36-8.28 (m, 2H), 7.22-7.14 (m, 1H), 3.63 (quint, *J* = 9.2 Hz, 1H), 2.26 (td, *J* = 9.2, 2.4 Hz, 2H), 1.84 (td, *J* = 9.5, 2.5 Hz, 2H), 1.70-1.62 (m, 2H), 1.48-1.41 (m, 2H), 1.31-1.25 (m, 2H), 1.20-1.14 (m, 2H), 0.89 (s, 6H). Spectrum
^{13}C NMR (75 MHz, $CDCl_3$): δ 158.4 (d, *J* = 254.1 Hz), 145.8 (d, *J* = 5.0 Hz), 142.0 (d, *J* = 13.3 Hz), 137.6 (d, *J* = 24.7 Hz), 122.6, 38.5, 36.5, 36.2, 36.0, 35.6, 32.6, 29.8, 28.4 (br), 27.6. Spectrum
^{19}F NMR (282 MHz, $CDCl_3$): δ –135.3. Spectrum
HRMS (EI$^+$): calculated for $C_{16}H_{22}FN$ $[M]^+$: 247.1736; found: 247.1748.

7.3 Trifluoroborate salt

Synthesis of Potassium Trifluoroborate Salt of 2m, 9

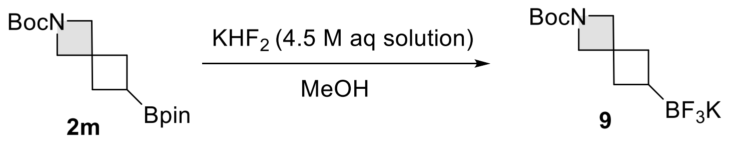

An oven-dried vial was charged with boronic ester **2m** (240 mg, 0.742 mmol, 1.0 equiv) and was dissolved methanol (13 mL). Then, KHF_2 (4.5 M aqueous solution, 0.97 mL) was added dropwise, and the resulting solution was stirred for 30 min at room temperature.[9] After this time, the residue was re-dissolved in methanol (6 mL) and water (4 mL). Again, all volatiles were removed under reduced pressure. This cycle was repeated (x10) to remove all pinacol. The solid that was obtained was triturated with acetone (3 x 5 mL) and filtered through a plug of Celite®. The solvent was evaporated to yield the trifluoroborate salt **9** (192 mg, 0.633 mmol) in 85% yield as a white solid.
mp = 226-228 °C.
^{1}H NMR (300 MHz, D_2O): δ 4.00-3.76 (m, 4H), 2.23-1.84 (m, 4H), 1.40 (s, 9H), 1.29-1.09 (m, 1H). Spectrum
^{13}C NMR (75 MHz, D_2O): δ 158.9, 82.0, 62.9 (br), 62.8 (br), 36.9, 34.5, 33.9, 28.3 [note: the carbon attached to boron was not observed due to quadrupole broadening caused by the ^{11}B nucleus]. Spectrum

HRMS (ESI$^-$): calculated for $C_{11}H_{18}BF_3NO_2$ [M-K]$^-$: 264.1383; found: 264.1387.

7.4 Homologation

Synthesis of *tert*-butyl 2-((4,4,5,5-tetramethyl-1,3,2-dioxaborolan-2-yl)methyl)-7-azaspiro[3.5]nonane-7-carboxylate, 10

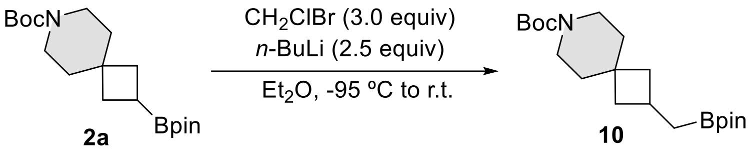

2a **10**

An oven-dried vial was charged with boronic ester **2a** (70.3 mg, 0.2 mmol, 1 equiv) and was dissolved in anhydrous diethyl ether (2 mL) under an argon atmosphere.[3] Then, bromochloromethane (39.0 µL, 0.6 mmol, 3 equiv) was added and the reaction mixture was cooled to −95 °C (methanol/liquid nitrogen bath). To this mixture, *n*-Butyllithium (0.59 mmol, 3 equiv) was added dropwise and the solution was stirred for other 10 minutes at −95 °C, followed by additional 1 h at room temperature. After this time, the whole mixture was filtered through a thin layer of silica and eluted with Et_2O. Once the solvent was evaporated under reduced pressure, the crude mixture was purified by flash column chromatography (SiO_2; hexane/EtOAc 90:10) to afford the homologated product **10** (48.6 mg, 0.133 mmol) in 67% yield as a white solid.

mp = 56-58 °C.

^{1}H NMR (300 MHz, $CDCl_3$): δ 3.31-3.12 (m, 4H), 2.32 (h, *J* = 8.3 Hz, 1H), 2.01-1.87 (m, 2H), 1.51-1.42 (m, 3H), 1.42-1.28 (m, 12H), 1.15 (s, 12H), 0.88 (d, *J* = 7.7 Hz, 2H). *Spectrum*

^{13}C NMR (75 MHz, $CDCl_3$): δ 155.2, 83.0, 79.2, 40.9 (br), 40.4, 39.9 (br), 36.3, 33.9, 28.6, 25.0. [note: the carbon attached to boron was not observed due to quadrupole broadening caused by the ^{11}B nucleus]. *Spectrum*

HRMS (ESI$^+$): calculated for $C_{20}H_{36}BNNaO_4$ [M+Na]$^+$: 388.2635; found: 388.2630.

7.5 Fluorination

Synthesis of 2-fluoro-7-thiaspiro[3.5]nonane 7,7-dioxide, 11

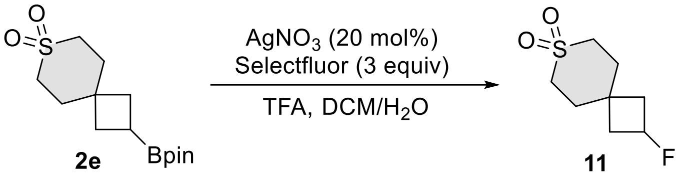

2e **11**

According to a literature procedure,[4] in an oven-dried vial was charged with boronic ester **2e** (36.0 mg, 0.12 mmol), $AgNO_3$ (4.1 mg, 0.024 mmol), and Selectfluor (127.5 mg, 0.36 mmol) in the glove box and sealed with a septum. Then, dichloromethane (0.6 mL), H_2O (0.6 mL), and TFA (37 µL, 0.48 mmol) were sequentially added. The reaction mixture was stirred at 50 °C for 6 hours under vigorous stirring. Upon completion, the reaction mixture was cooled to room temperature and extracted with EtOAc (4 × 15 mL). The combined organic layers were washed with brine, dried over $MgSO_4$, filtered, and concentrated under vacuum. The residue was

[3] Silvi, M.; Aggarwal, V. K. *J. Am. Chem. Soc.* **2019**, *141*, 9511−9515.
[4] Li, Z.; Wang, Z.; Zhu, L.; Tan, X.; Li, C. *J. Am. Chem. Soc.* **2014**, *136*, 16439−16443.

purified by column chromatography (SiO_2; CH_2Cl_2/MeOH 99:1) to afford the desired product **11** (19.0 mg, 0.099 mmol) as a white solid in 82% yield.

mp = 120-122 °C.

^{1}H NMR (300 MHz, $CDCl_3$): δ 5.06 (dtt, *J* = 54.8, 7.0, 5.5 Hz, 1H), 2.99-2.90 (m, 4H), 2.46-2.32 (m, 2H), 2.24-2.17 (m, 2H), 2.17-2.00 (m, 4H). *Spectrum*

^{13}C NMR (75 MHz, $CDCl_3$): δ 84.0 (d, *J* = 208.1 Hz), 48.5, 48.3, 39.6 (d, *J* = 21.1 Hz), 37.1, 34.7. *Spectrum*

^{19}F NMR (282 MHz, $CDCl_3$): δ –82.0. *Spectrum*

HRMS (ESI$^+$): calculated for $C_8H_{13}FNaO_2S$ $[M+Na]^+$: 215.0518; found: 215.0509.

8. Comparison of the Copper-Catalyzed Borylation of Cyclobutene 1a and Bromide SI-6

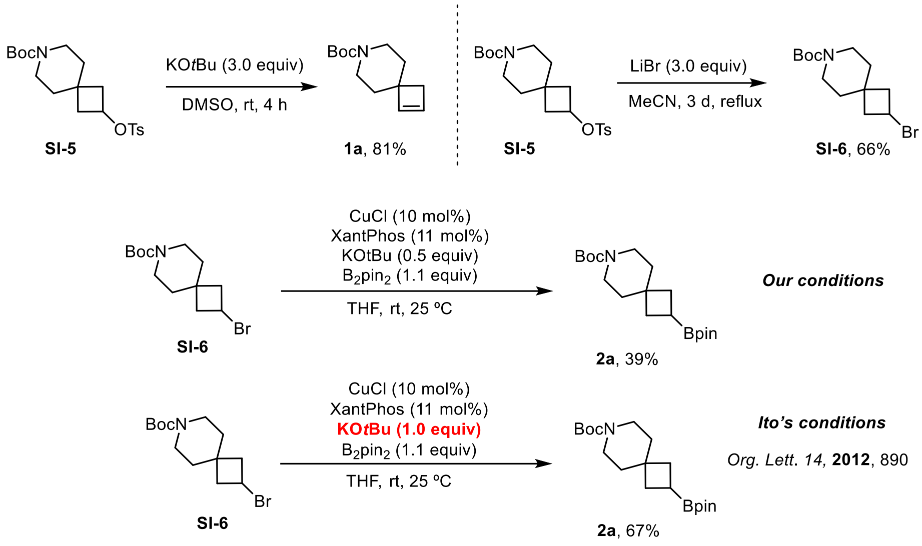

An alternative approach to prepare monoborylated spirocycles, as those describe in this work, could be through copper-catalyzed borylation of the corresponding bromide. To compare both approaches we prepared the spirocyclobutyl bromide **SI-6**.[5] In our hands, the preparation of the starting bromide compared to the cyclobutene is less convenient as it requires refluxing in CH_3CN for 3 days to obtain the product in only 66% yield. Regarding the borylation, when we used the conditions optimized for our cyclobutenes (0.5 equiv of KO*t*-Bu) the borylated product **2a** was obtained in only 39% yield. Increasing the amount of base to 1 equiv (as Ito published for alkyl bromides)[6] the yield of the borylated product increased to 67% yield. Although they are both valid approaches, perhaps the cyclobutene is more convenient as it doesn't require a stochiometric amount of base and opens the door to explore the difunctionalization of the double bond.

[5] Schmidt, V. A.; Quinn, R. K.; Brusoe, A. T.; Alexanian, E. J. *J. Am. Chem. Soc.* **2014**, *136*, 41, 14389-14392.
[6] Ito, H.; Kubota, K. *Org. Lett.* **2012**, *13*, 890-893.

9. Additional Computational Results and Computational Details

All the calculations reported in this paper were performed with the Gaussian 09 suite of programs.[7] Electron correlation was partially taken into account using the hybrid functional usually denoted as B3LYP[8] in conjunction with the D3 dispersion correction suggested by Grimme et al.[9] using the standard double-ζ quality def2-SVP[10] basis set for all atoms. Solvent effects (solvent = tetrahydrofuran) were taken into account by means of the Polarization Continuum Model (PCM)[11] method. This level is denoted PCM(tetrahydrofuran)-B3LYP-D3/def2-SVP. Geometries were fully optimized in solution without any geometry or symmetry constraints. Reactants, intermediates, and products were characterized by frequency calculations,[12] and have positive definite Hessian matrices. Transition structures (TS's) show only one negative eigenvalue in their diagonalized force constant matrices, and their associated eigenvectors were confirmed to correspond to the motion along the reaction coordinate under consideration using the Intrinsic Reaction Coordinate (IRC) method.

[7] Gaussian 09, Revision D.01, Frisch, M. J.; Trucks, G. W.; Schlegel, H. B.; Scuseria, G. E.; Robb, M. A.; Cheeseman, J. R.; Scalmani, G.; Barone, V.; Mennucci, B.; Petersson, G. A.; Nakatsuji, H.; Caricato, M.; Li, X.; Hratchian, H. P.; Izmaylov, A. F.; Bloino, J.; Zheng, G.; Sonnenberg, J. L.; Hada, M.; Ehara, M.; Toyota, K.; Fukuda, R.; Hasegawa, J.; Ishida, M.; Nakajima, T.; Honda, Y.; Kitao, O.; Nakai, H.; Vreven, T.; Montgomery, J. A., Jr.; Peralta, J. E.; Ogliaro, F.; Bearpark, M.; Heyd, J. J.; Brothers, E.; Kudin, K. N.; Staroverov, V. N.; Kobayashi, R.; Normand, J.; Raghavachari, K.; Rendell, A.; Burant, J. C.; Iyengar, S. S.; Tomasi, J.; Cossi, M.; Rega, N.; Millam, J. M.; Klene, M.; Knox, J. E.; Cross, J. B.; Bakken, V.; Adamo, C.; Jaramillo, J.; Gomperts, R.; Stratmann, R. E.; Yazyev, O.; Austin, A. J.; Cammi, R.; Pomelli, C.; Ochterski, J. W.; Martin, R. L.; Morokuma, K.; Zakrzewski, V. G.; Voth, G. A.; Salvador, P.; Dannenberg, J. J.; Dapprich, S.; Daniels, A. D.; Farkas, Ö.; Foresman, J. B.; Ortiz, J. V.; Cioslowski, J.; Fox, D. J. Gaussian, Inc., Wallingford CT, 2009.
[8] a) Becke, A. D. *J. Chem. Phys*. **1993**, *98*, 5648; b) Lee, C.; Yang, W.; Parr, R. G. *Phys. Rev. B* **1998**, *37*, 785; c) Vosko, S. H.; Wilk, L.; Nusair, M. *Can. J. Phys*. **1980**, *58*, 1200.
[9] Grimme, S.; Antony, J.; Ehrlich, S.; Krieg, H. *J. Chem. Phys*. **2010**, *132*, 154104.
[10] Weigend, F.; Ahlrichs, R. *Phys. Chem. Chem. Phys*. **2005**, *7*, 3297.

[11] (a) Miertuš, S.; Scrocco, E.; Tomasi, *Chem. Phys.* 1981, 55, 117; b) Pascual-Ahuir, J. L.; Silla, E.; Tuñón, I. *J. Comp. Chem*. **1994**, *15*, 1127; c) Barone, V.; Cossi, M. *J. Phys. Chem. A*, **1998**, *102*, 1995.
[12] McIver, J. W.; Komornicki, A. K. *J. Am. Chem. Soc.* **1972**, *94*, 2625.

(a) Profile involving **L2** = dppbz

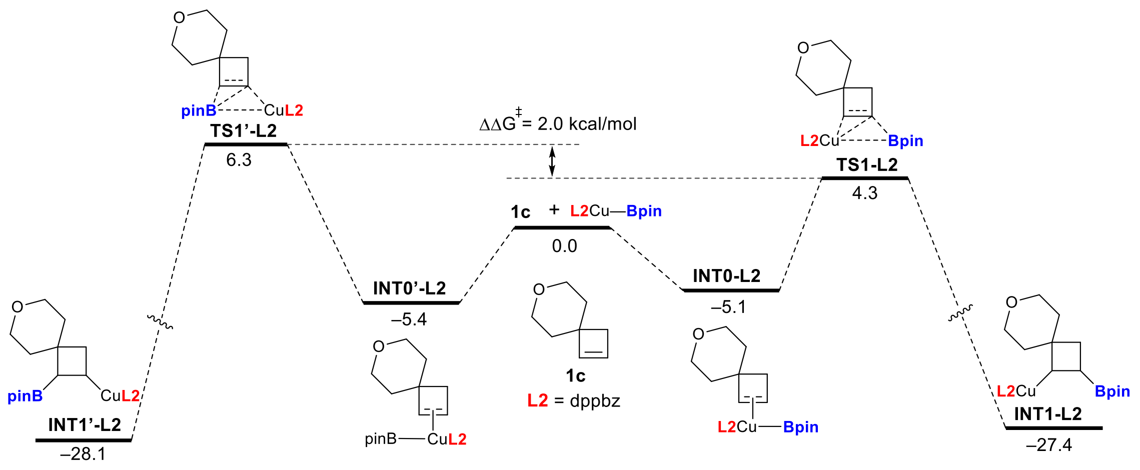

(b) Profile involving **L3** = xantphos

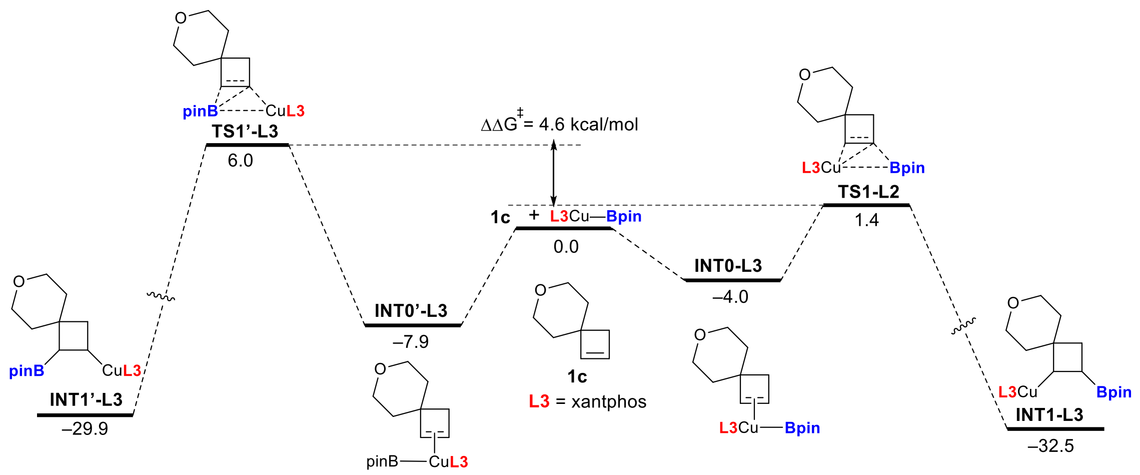

Figure S1. Computed reaction profiles for the key migratory insertion step involving 1a and L2Cu-Bpin **(a)** (L2 = dppbz) or L3Cu-Bpin **(b)** (L3 = xantphos). Relative free energy values are given in kcal/mol. All data have been computed at the PCM (tetrahydrofuran)-B3LYP-D3/def2-SVP level,

Cartesian coordinates (in Å) and free energies (in a.u.) of all the stationary points discussed in the text. All calculations have been performed at the PCM(tetrahydrofuran)-B3LYP-D3/def2-SVP level.

Profile involving L3 = xantphos

1c, G = -386.821020

C	1.986895000	-0.000564000	-0.870860000
C	2.635279000	0.000516000	0.305167000
H	2.308574000	-0.001522000	-1.917191000
C	1.368646000	0.000138000	1.140339000
H	3.693967000	0.000179000	0.581302000
H	1.202371000	-0.894876000	1.764298000
H	1.201751000	0.894926000	1.764451000
C	0.598814000	-0.000230000	-0.242890000
C	-0.255075000	1.250101000	-0.504099000
C	-0.255445000	-1.250400000	-0.503485000
C	-1.576030000	1.178176000	0.262542000
H	-0.472785000	1.316880000	-1.584635000
H	0.299002000	2.161244000	-0.222420000
C	-1.576535000	-1.177844000	0.262888000
H	-0.472991000	-1.317660000	-1.584041000
H	0.298342000	-2.161525000	-0.221260000
O	-2.300758000	0.000257000	-0.050706000
H	-2.228353000	2.028162000	0.008802000
H	-1.376643000	1.222462000	1.354561000
H	-2.229096000	-2.027660000	0.009196000
H	-1.377365000	-1.222024000	1.354964000

L3Cu-Bpin, G = -4312.844129

Cu	0.039582000	-0.051936000	-1.418607000
B	0.526923000	-2.021442000	-1.502444000
O	-0.252679000	-2.903704000	-0.751487000
O	1.733575000	-2.652913000	-1.818969000
C	0.411237000	-4.180793000	-0.618517000
C	1.899528000	-3.809882000	-0.962502000
C	0.183825000	-4.693360000	0.801641000
C	-0.225791000	-5.135200000	-1.634133000
C	2.694568000	-3.335445000	0.261019000
C	2.678833000	-4.882857000	-1.714269000
H	-0.886180000	-4.905423000	0.949815000
H	0.750821000	-5.619750000	0.985722000
H	0.475487000	-3.942086000	1.547259000
H	0.180844000	-6.154750000	-1.551334000
H	-1.310062000	-5.177617000	-1.449937000
H	-0.070015000	-4.775022000	-2.661595000
H	2.932200000	-4.163005000	0.946678000
H	3.637413000	-2.884036000	-0.081314000
H	2.142718000	-2.563707000	0.815812000
H	3.702748000	-4.528887000	-1.910248000
H	2.744944000	-5.808567000	-1.120650000
H	2.212920000	-5.117874000	-2.680285000
C	2.129716000	0.519390000	1.038814000
C	1.103837000	0.046628000	1.873143000
C	1.336146000	-0.726862000	3.019065000
C	2.667590000	-1.033323000	3.327111000
H	2.898974000	-1.638582000	4.204041000
C	3.714916000	-0.561836000	2.529283000
H	4.747193000	-0.805931000	2.790042000
C	3.450812000	0.209603000	1.395662000
H	4.275729000	0.553404000	0.769741000
P	1.665582000	1.359450000	-0.544650000
C	3.265472000	1.374107000	-1.455405000

C	3.572028000	0.222778000	-2.204558000
H	2.865144000	-0.611429000	-2.229077000
C	4.786820000	0.136202000	-2.890774000
H	5.018521000	-0.764742000	-3.464903000
C	5.698900000	1.196906000	-2.848282000
H	6.645063000	1.129875000	-3.391667000
C	5.396567000	2.344381000	-2.106698000
H	6.106912000	3.174322000	-2.066332000
C	4.187996000	2.432237000	-1.407849000
H	3.967351000	3.327674000	-0.823110000
C	1.382935000	3.115164000	-0.071342000
C	1.274419000	4.070619000	-1.100591000
H	1.443496000	3.773994000	-2.139871000
C	0.957173000	5.398101000	-0.809200000
H	0.886550000	6.128484000	-1.619363000
C	0.718370000	5.789545000	0.514047000
H	0.458254000	6.826190000	0.741835000
C	0.807490000	4.844797000	1.539647000
H	0.618406000	5.140233000	2.574879000
C	1.142604000	3.517197000	1.251906000
H	1.214143000	2.794939000	2.067337000
P	-2.116724000	0.372495000	-0.651166000
C	-3.460698000	-0.322535000	-1.694490000
C	-4.638390000	0.367874000	-2.018254000
H	-4.822922000	1.362269000	-1.606165000
C	-5.584671000	-0.214768000	-2.870168000
H	-6.498195000	0.332243000	-3.117874000
C	-5.365669000	-1.490329000	-3.398803000
H	-6.107324000	-1.944039000	-4.061353000
C	-4.191312000	-2.184030000	-3.077967000
H	-4.014198000	-3.181433000	-3.489070000
C	-3.237900000	-1.603379000	-2.239306000
H	-2.317487000	-2.139447000	-1.989673000
C	-2.726169000	2.019034000	-0.104123000
C	-3.725772000	2.190595000	0.868937000
H	-4.191574000	1.317065000	1.332106000
C	-4.120604000	3.475502000	1.251004000
H	-4.897056000	3.602726000	2.009935000
C	-3.520652000	4.598000000	0.665982000
H	-3.827718000	5.602054000	0.970145000
C	-2.520921000	4.433585000	-0.297811000
H	-2.033514000	5.303660000	-0.742091000
C	-2.121933000	3.148477000	-0.676277000
H	-1.316636000	3.013350000	-1.402048000
C	-2.229065000	-0.645794000	0.887929000
C	-1.167892000	-0.554038000	1.801006000
C	-3.237547000	-1.581148000	1.156874000
C	-1.057066000	-1.359631000	2.940697000
C	-3.157135000	-2.401527000	2.285173000
C	-2.073489000	-2.298157000	3.163048000
O	-0.184615000	0.368515000	1.522000000
H	-4.076687000	-1.687316000	0.467240000
H	-3.944508000	-3.133264000	2.480551000
H	-2.030486000	-2.954073000	4.033130000
C	0.125815000	-1.104307000	3.883143000
C	-0.224270000	0.121577000	4.771675000
H	0.617871000	0.360812000	5.440009000
H	-0.438128000	1.007212000	4.154471000
H	-1.113122000	-0.093899000	5.385225000
C	0.416173000	-2.313445000	4.781750000
H	0.670773000	-3.204621000	4.188020000
H	1.248672000	-2.099974000	5.466911000
H	-0.453892000	-2.549960000	5.410363000

INT0-L3, G = -4699.671554

Cu	0.743755000	0.312120000	-0.656777000
B	-0.483715000	-0.624365000	-2.000744000
O	-1.839985000	-0.321579000	-2.093178000
O	-0.188463000	-1.655771000	-2.891261000
C	-2.442295000	-1.048818000	-3.187899000
C	-1.421808000	-2.227569000	-3.399036000
C	-3.845864000	-1.475869000	-2.768966000
C	-2.515642000	-0.088295000	-4.380197000
C	-1.736693000	-3.448688000	-2.530228000
C	-1.210056000	-2.650362000	-4.848499000
H	-4.471594000	-0.583845000	-2.615703000
H	-4.315473000	-2.105472000	-3.541150000
H	-3.825953000	-2.028391000	-1.821234000
H	-3.004593000	-0.549683000	-5.251508000
H	-3.097521000	0.798746000	-4.087059000
H	-1.510391000	0.247526000	-4.674744000
H	-2.628804000	-3.985916000	-2.885854000
H	-0.880263000	-4.139120000	-2.557572000
H	-1.895216000	-3.155325000	-1.484780000
H	-0.480012000	-3.473334000	-4.891297000
H	-2.152165000	-3.006217000	-5.294821000
H	-0.822183000	-1.824003000	-5.458498000
C	-0.610925000	-2.371236000	1.088586000
C	-1.847311000	-1.719164000	1.205188000
C	-3.077864000	-2.391285000	1.219234000
C	-3.046758000	-3.786761000	1.100943000
H	-3.977920000	-4.354428000	1.099939000
C	-1.830633000	-4.470883000	1.004401000
H	-1.826530000	-5.560131000	0.922720000
C	-0.621333000	-3.771657000	1.003948000
H	0.319043000	-4.317531000	0.915825000
P	0.923346000	-1.340440000	0.986914000
C	2.171985000	-2.611802000	0.506228000
C	2.240790000	-2.940616000	-0.861682000
H	1.545464000	-2.475973000	-1.565964000
C	3.194540000	-3.853195000	-1.317510000
H	3.240607000	-4.098921000	-2.381628000
C	4.094508000	-4.441807000	-0.420146000
H	4.848665000	-5.146161000	-0.780440000
C	4.022618000	-4.125931000	0.940118000
H	4.716856000	-4.586828000	1.647590000
C	3.061682000	-3.219820000	1.404204000
H	3.016758000	-2.982068000	2.468584000
C	1.291295000	-0.977982000	2.761978000
C	2.506366000	-0.336746000	3.068600000
H	3.223152000	-0.126389000	2.273042000
C	2.805472000	0.040174000	4.378238000
H	3.758662000	0.528681000	4.596315000
C	1.880630000	-0.190162000	5.404237000
H	2.106267000	0.118670000	6.427951000
C	0.664937000	-0.812092000	5.108379000
H	-0.064896000	-0.994228000	5.901498000
C	0.373537000	-1.210807000	3.798479000
H	-0.576974000	-1.705416000	3.591883000
P	-0.764651000	2.049780000	0.170582000
C	-0.760242000	3.426631000	-1.049501000
C	-0.281905000	4.715555000	-0.769120000
H	0.024841000	4.977860000	0.245844000
C	-0.196964000	5.674908000	-1.786304000
H	0.178100000	6.675345000	-1.554738000

C	-0.591792000	5.358949000	-3.089165000
H	-0.526616000	6.110324000	-3.880319000
C	-1.069579000	4.073002000	-3.376426000
H	-1.377622000	3.816290000	-4.393574000
C	-1.145638000	3.110812000	-2.368732000
H	-1.500861000	2.101958000	-2.593696000
C	-0.471981000	2.864289000	1.797207000
C	-1.396846000	3.725338000	2.412871000
H	-2.345601000	3.949170000	1.918436000
C	-1.109985000	4.290724000	3.657833000
H	-1.833818000	4.958884000	4.131938000
C	0.100050000	3.997292000	4.301226000
H	0.318973000	4.435801000	5.278396000
C	1.021456000	3.136593000	3.697127000
H	1.958322000	2.887802000	4.200457000
C	0.734952000	2.573004000	2.449652000
H	1.437119000	1.883542000	1.976297000
C	2.466298000	1.741150000	-0.926054000
C	1.888095000	1.321810000	-2.123921000
H	2.275928000	2.615895000	-0.297668000
C	3.110334000	0.501311000	-2.528353000
H	1.144677000	1.792040000	-2.769387000
H	2.972519000	-0.589622000	-2.554919000
H	3.582436000	0.821707000	-3.472111000
C	3.809065000	1.068465000	-1.232723000
C	4.913685000	2.090879000	-1.561671000
C	4.386630000	0.050824000	-0.250347000
C	6.197736000	1.389542000	-2.009348000
H	5.128888000	2.688239000	-0.657801000
H	4.574233000	2.791049000	-2.343543000
C	5.688756000	-0.549869000	-0.773266000
H	4.590495000	0.558741000	0.708802000
H	3.659717000	-0.746264000	-0.061412000
O	6.655511000	0.452843000	-1.048443000
H	7.013437000	2.114744000	-2.157396000
H	6.022439000	0.880306000	-2.980903000
H	6.137615000	-1.229734000	-0.032565000
H	5.483068000	-1.145249000	-1.688417000
C	-2.553446000	1.606940000	0.254214000
C	-2.862526000	0.355416000	0.801444000
C	-3.607889000	2.358353000	-0.282802000
C	-4.148858000	-0.196024000	0.798656000
C	-4.906611000	1.841924000	-0.296550000
C	-5.171730000	0.572376000	0.228246000
O	-1.814697000	-0.356304000	1.334945000
H	-3.409552000	3.338461000	-0.720269000
H	-5.720480000	2.431030000	-0.725962000
H	-6.191231000	0.186509000	0.196384000
C	-4.343321000	-1.560264000	1.471284000
C	-4.456153000	-1.327281000	3.003861000
H	-4.561551000	-2.289219000	3.529964000
H	-3.560914000	-0.817661000	3.390969000
H	-5.333562000	-0.701318000	3.230418000
C	-5.609045000	-2.272612000	0.975469000
H	-5.568499000	-2.460825000	-0.108032000
H	-5.745501000	-3.233409000	1.491690000
H	-6.503997000	-1.671849000	1.190897000

INT0′-L3, G = -4699.677689

Cu	-0.901554000	0.157474000	-0.487816000
B	-1.186485000	-0.134872000	1.517094000
O	-0.483301000	0.514348000	2.530485000
O	-1.980604000	-1.129143000	2.099216000

C	-0.942035000	0.071793000	3.827493000
C	-1.603170000	-1.312348000	3.484569000
C	0.255785000	-0.002315000	4.769920000
C	-1.946260000	1.116408000	4.328662000
C	-0.607218000	-2.476433000	3.525591000
C	-2.849925000	-1.652729000	4.293435000
H	0.659044000	1.008063000	4.935335000
H	-0.034887000	-0.425899000	5.744324000
H	1.062902000	-0.611456000	4.342981000
H	-2.310380000	0.885649000	5.341228000
H	-1.452861000	2.100038000	4.354650000
H	-2.809108000	1.188552000	3.650087000
H	-0.310162000	-2.726828000	4.555295000
H	-1.074751000	-3.363514000	3.072847000
H	0.295256000	-2.243831000	2.944369000
H	-3.241021000	-2.631584000	3.975667000
H	-2.617955000	-1.709915000	5.368761000
H	-3.643202000	-0.908480000	4.143574000
C	1.272571000	-2.515115000	0.080271000
C	2.173604000	-1.665975000	0.742349000
C	2.977092000	-2.081071000	1.814613000
C	2.859363000	-3.413949000	2.227807000
H	3.458530000	-3.780830000	3.061750000
C	1.992010000	-4.293596000	1.572362000
H	1.922181000	-5.332692000	1.901927000
C	1.208369000	-3.851215000	0.504604000
H	0.523734000	-4.543696000	0.012970000
P	0.152335000	-1.786274000	-1.196338000
C	-1.054044000	-3.154049000	-1.462692000
C	-2.029741000	-3.330899000	-0.462932000
H	-2.023539000	-2.687799000	0.420941000
C	-2.997954000	-4.328834000	-0.592497000
H	-3.749509000	-4.455088000	0.191207000
C	-3.013809000	-5.154658000	-1.723094000
H	-3.778197000	-5.928937000	-1.827931000
C	-2.044984000	-4.985606000	-2.716918000
H	-2.045898000	-5.630574000	-3.599534000
C	-1.065219000	-3.994253000	-2.586579000
H	-0.310793000	-3.882011000	-3.367171000
C	1.171280000	-1.754282000	-2.728930000
C	0.534049000	-1.381378000	-3.929011000
H	-0.544268000	-1.204503000	-3.938892000
C	1.267312000	-1.228810000	-5.106676000
H	0.756361000	-0.945694000	-6.030433000
C	2.654634000	-1.422283000	-5.100346000
H	3.232011000	-1.288821000	-6.018503000
C	3.296368000	-1.781611000	-3.912049000
H	4.378976000	-1.931375000	-3.897831000
C	2.560485000	-1.953525000	-2.734101000
H	3.077677000	-2.239835000	-1.816319000
P	0.770357000	1.995388000	-0.424757000
C	-0.187235000	3.522403000	-0.052139000
C	-0.328349000	4.574277000	-0.971364000
H	0.259868000	4.581247000	-1.891492000
C	-1.229005000	5.617278000	-0.722390000
H	-1.329226000	6.428700000	-1.448207000
C	-1.997309000	5.622584000	0.445374000
H	-2.701355000	6.436566000	0.636483000
C	-1.860373000	4.576951000	1.368361000
H	-2.459102000	4.570581000	2.283254000
C	-0.968774000	3.531398000	1.122317000
H	-0.881944000	2.703775000	1.829842000
C	1.936305000	2.507699000	-1.764164000

C	2.901893000	3.516795000	-1.596603000
H	2.980427000	4.042734000	-0.641683000
C	3.765280000	3.848479000	-2.642949000
H	4.512168000	4.634569000	-2.503446000
C	3.678510000	3.172067000	-3.867687000
H	4.358697000	3.430197000	-4.683620000
C	2.726913000	2.163386000	-4.039941000
H	2.659630000	1.619078000	-4.984645000
C	1.860342000	1.833653000	-2.991480000
H	1.132270000	1.031948000	-3.120537000
C	-1.966925000	0.738237000	-2.354048000
C	-2.582544000	1.289321000	-1.242481000
H	-1.193883000	1.119246000	-3.024766000
H	-2.458057000	2.248078000	-0.735313000
C	-3.118907000	-0.228159000	-2.628508000
C	1.916244000	1.853088000	1.014322000
C	2.519089000	0.605403000	1.222331000
C	2.174531000	2.863679000	1.950138000
C	3.337489000	0.316335000	2.321287000
C	2.982802000	2.608310000	3.060863000
C	3.551541000	1.344276000	3.248508000
O	2.258144000	-0.374563000	0.291347000
H	1.717750000	3.847120000	1.825895000
H	3.169087000	3.399774000	3.790746000
H	4.174575000	1.167479000	4.125953000
C	3.982665000	-1.073253000	2.385722000
C	5.227356000	-1.067754000	1.455219000
H	5.701900000	-2.061685000	1.446942000
H	4.945235000	-0.810426000	0.423099000
H	5.962434000	-0.326621000	1.806390000
C	4.417902000	-1.439582000	3.810655000
H	3.561477000	-1.452536000	4.501981000
H	4.899019000	-2.427664000	3.832284000
H	5.159825000	-0.721957000	4.188375000
C	-3.850432000	0.435605000	-1.407203000
C	-5.066157000	1.300775000	-1.778917000
C	-4.249938000	-0.478451000	-0.248760000
C	-5.587082000	2.037376000	-0.541226000
H	-5.866705000	0.653424000	-2.178175000
H	-4.802610000	2.030640000	-2.563385000
C	-4.790270000	0.351955000	0.909547000
H	-5.027274000	-1.185489000	-0.590081000
H	-3.383050000	-1.053066000	0.092946000
H	-6.509612000	2.593079000	-0.772582000
H	-4.829740000	2.776335000	-0.202725000
H	-5.135558000	-0.288152000	1.735939000
H	-3.975080000	0.994322000	1.303355000
O	-5.902784000	1.147407000	0.515886000
H	-3.631489000	-0.064845000	-3.592034000
H	-2.909080000	-1.303268000	-2.513744000

TS1-L3, G = -4699.662976

Cu	0.750035000	0.466992000	-0.325586000
B	0.107591000	-0.340599000	-2.090764000
O	-1.097348000	0.055128000	-2.651006000
O	0.575528000	-1.480190000	-2.729834000
C	-1.383389000	-0.757066000	-3.814940000
C	-0.484681000	-2.024765000	-3.557247000
C	-2.884090000	-1.026725000	-3.852366000
C	-0.949459000	0.051135000	-5.041081000
C	-1.188956000	-3.101863000	-2.727111000
C	0.134844000	-2.640853000	-4.806506000
H	-3.423957000	-0.087526000	-4.045541000

H	-3.136773000	-1.741606000	-4.650993000
H	-3.241105000	-1.420243000	-2.891591000
H	-1.177572000	-0.473955000	-5.980544000
H	-1.485862000	1.012204000	-5.042365000
H	0.129494000	0.264161000	-5.009748000
H	-1.977176000	-3.610333000	-3.301846000
H	-0.449228000	-3.851707000	-2.411687000
H	-1.634262000	-2.675757000	-1.818306000
H	0.739322000	-3.517191000	-4.526657000
H	-0.648011000	-2.974861000	-5.505437000
H	0.791057000	-1.930180000	-5.325782000
C	-0.548119000	-2.490004000	0.988437000
C	-1.855131000	-1.984433000	0.917102000
C	-2.982560000	-2.784043000	0.679648000
C	-2.771942000	-4.158493000	0.514441000
H	-3.616188000	-4.820906000	0.320544000
C	-1.486385000	-4.700640000	0.608823000
H	-1.341681000	-5.776644000	0.488152000
C	-0.384090000	-3.876982000	0.847951000
H	0.614154000	-4.313017000	0.902063000
P	0.864882000	-1.298738000	1.112795000
C	2.306325000	-2.423374000	0.855058000
C	2.599263000	-2.785376000	-0.473520000
H	1.963001000	-2.428952000	-1.287386000
C	3.700250000	-3.596899000	-0.755394000
H	3.919194000	-3.867510000	-1.791599000
C	4.526581000	-4.051142000	0.279801000
H	5.395478000	-4.675437000	0.056410000
C	4.232735000	-3.703980000	1.601805000
H	4.867232000	-4.061259000	2.417037000
C	3.124117000	-2.899464000	1.890663000
H	2.906664000	-2.640971000	2.928179000
C	0.935364000	-0.871696000	2.905987000
C	2.039698000	-0.123419000	3.354617000
H	2.828599000	0.152498000	2.650909000
C	2.134184000	0.272425000	4.688686000
H	3.004217000	0.842708000	5.024062000
C	1.107068000	-0.038945000	5.588042000
H	1.171159000	0.287166000	6.629126000
C	-0.005130000	-0.758592000	5.144644000
H	-0.814958000	-0.999176000	5.838187000
C	-0.088040000	-1.182469000	3.812956000
H	-0.956389000	-1.758270000	3.487694000
P	-1.026881000	1.911043000	0.212492000
C	-0.964450000	3.468442000	-0.768910000
C	-0.786544000	4.732765000	-0.185824000
H	-0.757585000	4.835651000	0.900883000
C	-0.643208000	5.868985000	-0.991284000
H	-0.503270000	6.847487000	-0.524271000
C	-0.681587000	5.755802000	-2.384044000
H	-0.570340000	6.644503000	-3.010623000
C	-0.861192000	4.497186000	-2.971920000
H	-0.890910000	4.399393000	-4.060473000
C	-0.993312000	3.359672000	-2.173163000
H	-1.124978000	2.376539000	-2.630905000
C	-1.119799000	2.490348000	1.955524000
C	-2.319370000	2.857433000	2.587216000
H	-3.264388000	2.799782000	2.041633000
C	-2.308418000	3.294328000	3.915045000
H	-3.246233000	3.573382000	4.402711000
C	-1.100705000	3.375185000	4.618706000
H	-1.094931000	3.714697000	5.657782000
C	0.096266000	3.011404000	3.993397000

H	1.039212000	3.055105000	4.542186000
C	0.085691000	2.563421000	2.669984000
H	1.010375000	2.250601000	2.177564000
C	2.245097000	1.864956000	-0.817490000
C	1.728666000	1.154282000	-1.983857000
H	2.025165000	2.908207000	-0.561839000
C	3.090011000	0.478092000	-2.217742000
H	1.168504000	1.620390000	-2.803698000
H	3.113605000	-0.613433000	-2.100832000
H	3.536453000	0.731475000	-3.193016000
C	3.665273000	1.316238000	-1.019828000
C	4.682894000	2.379364000	-1.468934000
C	4.299559000	0.517429000	0.123625000
C	6.047351000	1.755912000	-1.768942000
H	4.799647000	3.123418000	-0.660220000
H	4.311691000	2.918968000	-2.357063000
C	5.675203000	-0.018450000	-0.261971000
H	4.398884000	1.176539000	1.005027000
H	3.640544000	-0.313149000	0.404719000
O	6.553225000	1.023544000	-0.664293000
H	6.796987000	2.528508000	-2.003944000
H	5.963783000	1.091795000	-2.656216000
H	6.159425000	-0.531154000	0.584210000
H	5.565733000	-0.762102000	-1.080350000
C	-2.726872000	1.276427000	-0.109015000
C	-2.997409000	-0.020994000	0.345656000
C	-3.720800000	1.919934000	-0.858872000
C	-4.182549000	-0.711286000	0.066432000
C	-4.915161000	1.258693000	-1.160428000
C	-5.140547000	-0.046980000	-0.711250000
O	-2.011730000	-0.631838000	1.087074000
H	-3.552135000	2.932310000	-1.229481000
H	-5.679032000	1.765023000	-1.755298000
H	-6.079016000	-0.542230000	-0.963000000
C	-4.356135000	-2.100206000	0.691995000
C	-4.780587000	-1.909688000	2.174632000
H	-4.877221000	-2.887775000	2.671645000
H	-4.035586000	-1.314316000	2.723579000
H	-5.748484000	-1.387158000	2.229676000
C	-5.420393000	-2.930956000	-0.036003000
H	-5.152639000	-3.094491000	-1.091057000
H	-5.550725000	-3.909543000	0.447382000
H	-6.397907000	-2.429607000	0.001990000

TS1'-L3, G = -4699.655603

Cu	-0.600969000	0.029528000	-0.863694000
B	-1.753670000	0.687420000	0.711163000
O	-1.580385000	1.935281000	1.295944000
O	-2.333648000	-0.180021000	1.628376000
C	-2.161753000	1.944374000	2.622889000
C	-2.228187000	0.407115000	2.951991000
C	-1.256349000	2.756907000	3.542582000
C	-3.539561000	2.604355000	2.515090000
C	-0.929093000	-0.122116000	3.568494000
C	-3.427559000	-0.029349000	3.784049000
H	-1.276916000	3.815498000	3.242157000
H	-1.600127000	2.690158000	4.586595000
H	-0.215203000	2.416923000	3.485284000
H	-4.018438000	2.706554000	3.500252000
H	-3.421429000	3.608407000	2.080521000
H	-4.207856000	2.029142000	1.859496000
H	-0.799282000	0.220075000	4.605705000

H	-0.943476000	-1.221753000	3.561101000
H	-0.059639000	0.202393000	2.982642000
H	-3.391057000	-1.118298000	3.940723000
H	-3.411271000	0.458250000	4.771184000
H	-4.378279000	0.209798000	3.290920000
C	1.084142000	-2.317806000	1.207040000
C	1.904991000	-1.301998000	1.721723000
C	2.448703000	-1.325875000	3.015063000
C	2.181853000	-2.451059000	3.803077000
H	2.580645000	-2.514778000	4.815871000
C	1.418327000	-3.510260000	3.301884000
H	1.237260000	-4.390278000	3.923309000
C	0.874535000	-3.446027000	2.017709000
H	0.261184000	-4.270772000	1.653546000
P	0.220045000	-2.045725000	-0.412124000
C	-1.044662000	-3.392038000	-0.359495000
C	-2.068852000	-3.267704000	0.598171000
H	-2.091091000	-2.392732000	1.251638000
C	-3.060050000	-4.244973000	0.704462000
H	-3.848783000	-4.134296000	1.453274000
C	-3.054961000	-5.350857000	-0.153986000
H	-3.836174000	-6.111384000	-0.076536000
C	-2.045722000	-5.475813000	-1.112871000
H	-2.032400000	-6.336496000	-1.786654000
C	-1.040348000	-4.506264000	-1.212433000
H	-0.251938000	-4.629021000	-1.956768000
C	1.428021000	-2.642643000	-1.668626000
C	1.046807000	-2.550787000	-3.019569000
H	0.076825000	-2.114348000	-3.275219000
C	1.903061000	-2.995809000	-4.027241000
H	1.592117000	-2.928320000	-5.072906000
C	3.167469000	-3.501461000	-3.700626000
H	3.846407000	-3.832312000	-4.490636000
C	3.562853000	-3.570698000	-2.361857000
H	4.552219000	-3.955571000	-2.101495000
C	2.693297000	-3.153323000	-1.347153000
H	3.005916000	-3.226622000	-0.303205000
P	1.064191000	1.684047000	-0.888107000
C	0.542697000	3.316310000	-1.568498000
C	1.156049000	3.903296000	-2.687522000
H	2.017454000	3.423047000	-3.155418000
C	0.667930000	5.104898000	-3.213741000
H	1.154084000	5.548199000	-4.086817000
C	-0.432138000	5.737041000	-2.626752000
H	-0.811528000	6.675347000	-3.039669000
C	-1.045611000	5.159623000	-1.508524000
H	-1.907110000	5.646300000	-1.043326000
C	-0.569268000	3.954896000	-0.985770000
H	-1.054535000	3.502097000	-0.118695000
C	2.548248000	1.256862000	-1.893214000
C	3.855855000	1.630067000	-1.542942000
H	4.035748000	2.205023000	-0.631629000
C	4.933296000	1.264164000	-2.355475000
H	5.948933000	1.552432000	-2.071647000
C	4.712999000	0.532837000	-3.528364000
H	5.556775000	0.245367000	-4.161089000
C	3.411539000	0.164025000	-3.884291000
H	3.233927000	-0.420702000	-4.789310000
C	2.335306000	0.517236000	-3.066356000
H	1.317084000	0.210623000	-3.318218000
C	-1.670123000	0.503218000	-2.542283000
C	-2.414504000	0.910016000	-1.322999000
H	-1.230147000	1.236456000	-3.227265000

H	-2.573779000	1.962718000	-1.061392000
C	-2.771310000	-0.474787000	-2.969783000
C	1.797485000	2.100201000	0.748217000
C	2.247254000	1.009137000	1.505119000
C	1.872180000	3.371100000	1.332148000
C	2.764259000	1.121206000	2.800147000
C	2.358936000	3.516951000	2.634905000
C	2.797813000	2.405301000	3.362482000
O	2.151712000	-0.226415000	0.907776000
H	1.530639000	4.245641000	0.775962000
H	2.399145000	4.509553000	3.089420000
H	3.182463000	2.549192000	4.372759000
C	3.332807000	-0.147901000	3.446970000
C	4.756372000	-0.377244000	2.868073000
H	5.190530000	-1.302462000	3.278621000
H	4.725173000	-0.466893000	1.771774000
H	5.413641000	0.467729000	3.127022000
C	3.419848000	-0.024424000	4.974036000
H	2.427690000	0.137058000	5.422360000
H	3.859789000	-0.929312000	5.416567000
H	4.072300000	0.812735000	5.259968000
C	-3.641804000	0.054867000	-1.793661000
C	-4.755312000	0.993364000	-2.296252000
C	-4.275817000	-0.989300000	-0.876176000
C	-5.589981000	1.539793000	-1.141142000
H	-5.422428000	0.422603000	-2.966425000
H	-4.325114000	1.821545000	-2.884475000
C	-5.186501000	-0.366459000	0.178479000
H	-4.867147000	-1.681868000	-1.502602000
H	-3.495002000	-1.580585000	-0.384835000
H	-6.419268000	2.163597000	-1.510776000
H	-4.958532000	2.178617000	-0.487539000
H	-5.728986000	-1.143141000	0.740603000
H	-4.568607000	0.193106000	0.899820000
O	-6.172453000	0.491178000	-0.384870000
H	-3.226731000	-0.295332000	-3.960634000
H	-2.527360000	-1.550459000	-2.900137000

INT1-L3, G = -4699.716862

Cu	-0.868167000	0.389197000	-0.268391000
B	-1.469247000	2.502819000	1.991389000
O	-0.394260000	3.364755000	1.919889000
O	-1.300657000	1.569382000	2.992854000
C	0.485701000	3.119746000	3.042429000
C	0.057440000	1.666895000	3.496545000
C	1.930022000	3.238411000	2.567861000
C	0.180435000	4.196202000	4.088401000
C	0.872910000	0.564620000	2.821112000
C	0.032721000	1.443112000	5.004807000
H	2.129939000	4.272226000	2.248127000
H	2.629629000	2.988886000	3.380334000
H	2.134709000	2.579691000	1.715862000
H	0.843727000	4.112873000	4.961723000
H	0.327408000	5.186424000	3.632044000
H	-0.862781000	4.131769000	4.431274000
H	1.918082000	0.558708000	3.160282000
H	0.433126000	-0.411386000	3.062322000
H	0.865960000	0.674169000	1.727000000
H	-0.282050000	0.410249000	5.217487000
H	1.034490000	1.591915000	5.436198000
H	-0.670988000	2.121295000	5.504332000
C	0.891707000	-2.453303000	1.278321000

C	2.159155000	-1.929523000	0.982917000
C	3.299475000	-2.167629000	1.763747000
C	3.146160000	-2.985236000	2.889674000
H	4.001586000	-3.195241000	3.532337000
C	1.904181000	-3.550471000	3.198350000
H	1.804929000	-4.194951000	4.074752000
C	0.787036000	-3.288963000	2.402434000
H	-0.179276000	-3.716119000	2.672922000
P	-0.572137000	-1.850917000	0.317289000
C	-1.962041000	-2.475924000	1.352015000
C	-2.427088000	-1.607589000	2.355381000
H	-1.986996000	-0.612836000	2.464136000
C	-3.473493000	-1.997779000	3.194306000
H	-3.829166000	-1.311731000	3.967198000
C	-4.076327000	-3.250361000	3.029566000
H	-4.905053000	-3.549877000	3.676265000
C	-3.619155000	-4.116705000	2.030466000
H	-4.086926000	-5.095666000	1.897734000
C	-2.560728000	-3.735682000	1.198179000
H	-2.207680000	-4.421066000	0.424843000
C	-0.598345000	-2.905015000	-1.191769000
C	-1.780380000	-2.947698000	-1.954449000
H	-2.667068000	-2.409128000	-1.615314000
C	-1.832055000	-3.681836000	-3.140594000
H	-2.760456000	-3.713040000	-3.716503000
C	-0.698057000	-4.363808000	-3.595290000
H	-0.734969000	-4.929342000	-4.529644000
C	0.485654000	-4.307539000	-2.854572000
H	1.379280000	-4.827681000	-3.208348000
C	0.536612000	-3.586897000	-1.657320000
H	1.467832000	-3.558210000	-1.089312000
P	1.065572000	1.127166000	-1.460474000
C	0.942979000	2.835309000	-2.129092000
C	1.341221000	3.191824000	-3.427726000
H	1.745788000	2.435419000	-4.104230000
C	1.223021000	4.516586000	-3.861147000
H	1.532337000	4.784642000	-4.874825000
C	0.713310000	5.495448000	-3.000723000
H	0.621957000	6.530067000	-3.341682000
C	0.316078000	5.144537000	-1.705404000
H	-0.087442000	5.902562000	-1.028972000
C	0.420196000	3.819729000	-1.271883000
H	0.096702000	3.552539000	-0.263598000
C	1.256462000	0.073289000	-2.954395000
C	2.498380000	-0.294547000	-3.495189000
H	3.423193000	0.054597000	-3.029884000
C	2.558336000	-1.108585000	-4.631161000
H	3.530192000	-1.392693000	-5.043298000
C	1.380611000	-1.555274000	-5.239115000
H	1.429152000	-2.194471000	-6.124319000
C	0.139858000	-1.194519000	-4.702627000
H	-0.782762000	-1.558402000	-5.159018000
C	0.077345000	-0.392122000	-3.562274000
H	-0.893367000	-0.135096000	-3.128209000
C	-2.376366000	1.733721000	-0.349730000
C	-2.653858000	2.557666000	0.987833000
H	-2.164098000	2.401396000	-1.207798000
C	-3.920121000	1.688979000	1.183944000
H	-2.925715000	3.607366000	0.766036000
H	-3.735867000	0.830717000	1.846641000
H	-4.830567000	2.203968000	1.535883000
C	-3.864548000	1.273648000	-0.315184000
C	-4.787652000	2.143075000	-1.190517000

C	-4.167829000	-0.189330000	-0.645574000
C	-6.251779000	1.716962000	-1.072870000
H	-4.471242000	2.041269000	-2.244730000
H	-4.688056000	3.209196000	-0.922700000
C	-5.657696000	-0.502279000	-0.545456000
H	-3.822641000	-0.391792000	-1.676192000
H	-3.597290000	-0.847055000	0.024315000
O	-6.432719000	0.343478000	-1.383593000
H	-6.892099000	2.282224000	-1.769465000
H	-6.619270000	1.920930000	-0.044281000
H	-5.875459000	-1.537074000	-0.855856000
H	-5.987526000	-0.395943000	0.510866000
C	2.741293000	1.119054000	-0.701216000
C	3.126300000	-0.055281000	-0.038212000
C	3.605549000	2.221521000	-0.622809000
C	4.309872000	-0.180741000	0.697374000
C	4.789064000	2.135094000	0.115981000
C	5.135981000	0.949233000	0.773680000
O	2.255242000	-1.116386000	-0.119778000
H	3.338467000	3.154890000	-1.121581000
H	5.450229000	3.002246000	0.181640000
H	6.066329000	0.907379000	1.341083000
C	4.626575000	-1.559945000	1.289289000
C	5.182837000	-2.454750000	0.146599000
H	5.383508000	-3.470224000	0.522573000
H	4.464694000	-2.529445000	-0.683855000
H	6.120552000	-2.031361000	-0.246234000
C	5.663316000	-1.480889000	2.416900000
H	5.307581000	-0.857325000	3.251137000
H	5.895563000	-2.482893000	2.804743000
H	6.608965000	-1.060597000	2.046049000

INT1'-L3, G = -4699.712740

Cu	0.500500000	0.070253000	-0.900649000
B	3.038235000	-0.954892000	0.662364000
O	2.699343000	-2.192474000	1.168729000
O	2.841354000	0.038626000	1.600339000
C	2.418287000	-2.068930000	2.582804000
C	2.139910000	-0.520230000	2.740801000
C	1.235423000	-2.967988000	2.925699000
C	3.675293000	-2.535475000	3.323400000
C	0.661576000	-0.157302000	2.593118000
C	2.702840000	0.111941000	4.009335000
H	1.512218000	-4.020140000	2.760373000
H	0.948672000	-2.849580000	3.981826000
H	0.362145000	-2.748625000	2.299605000
H	3.536346000	-2.513546000	4.414162000
H	3.903705000	-3.568310000	3.021151000
H	4.541395000	-1.907681000	3.066009000
H	0.061346000	-0.529272000	3.435388000
H	0.557151000	0.934797000	2.556678000
H	0.239653000	-0.565894000	1.664283000
H	2.466281000	1.186703000	4.018763000
H	2.253911000	-0.345657000	4.904227000
H	3.793668000	0.004860000	4.067231000
C	-1.572069000	2.200557000	1.302564000
C	-2.407475000	1.134032000	1.667407000
C	-3.058250000	1.045655000	2.906637000
C	-2.867635000	2.099118000	3.808791000
H	-3.348437000	2.075569000	4.787302000
C	-2.068432000	3.195373000	3.466646000
H	-1.937884000	4.013724000	4.178487000

C	-1.424441000	3.246828000	2.228715000
H	-0.780728000	4.094559000	1.990969000
P	-0.556867000	2.044343000	-0.236755000
C	0.627234000	3.438780000	-0.033369000
C	1.849514000	3.137497000	0.590561000
H	2.074957000	2.105731000	0.872302000
C	2.784725000	4.148383000	0.829542000
H	3.734993000	3.902407000	1.309880000
C	2.515078000	5.463001000	0.433637000
H	3.251135000	6.251372000	0.611230000
C	1.304054000	5.765632000	-0.200147000
H	1.091952000	6.790423000	-0.515967000
C	0.360309000	4.759396000	-0.429536000
H	-0.585325000	5.003718000	-0.918896000
C	-1.660271000	2.593072000	-1.599501000
C	-1.066382000	2.837015000	-2.852186000
H	0.016473000	2.736967000	-2.968287000
C	-1.849805000	3.208759000	-3.945501000
H	-1.375497000	3.404485000	-4.910561000
C	-3.238925000	3.315721000	-3.809572000
H	-3.854351000	3.595175000	-4.668320000
C	-3.836791000	3.051315000	-2.574364000
H	-4.921838000	3.122140000	-2.464264000
C	-3.053020000	2.696507000	-1.471393000
H	-3.532655000	2.499397000	-0.510543000
P	-1.087711000	-1.661083000	-0.981075000
C	-0.441412000	-3.261251000	-1.617047000
C	-1.172099000	-4.116160000	-2.458296000
H	-2.177874000	-3.838670000	-2.782432000
C	-0.614037000	-5.325250000	-2.885530000
H	-1.186608000	-5.984368000	-3.543385000
C	0.672243000	-5.691923000	-2.472071000
H	1.105406000	-6.637889000	-2.807559000
C	1.403062000	-4.842894000	-1.633405000
H	2.409203000	-5.120269000	-1.308378000
C	0.853627000	-3.627807000	-1.213967000
H	1.428631000	-2.964146000	-0.565660000
C	-2.419065000	-1.216554000	-2.167665000
C	-3.770001000	-1.552068000	-1.987413000
H	-4.082240000	-2.112093000	-1.102881000
C	-4.722781000	-1.169514000	-2.937355000
H	-5.773181000	-1.432493000	-2.787127000
C	-4.333800000	-0.456641000	-4.076237000
H	-5.079963000	-0.156461000	-4.816282000
C	-2.989383000	-0.114610000	-4.257924000
H	-2.682510000	0.462451000	-5.132434000
C	-2.038534000	-0.484488000	-3.305329000
H	-0.992908000	-0.189616000	-3.432408000
C	2.324118000	-0.141137000	-1.709992000
C	3.489176000	-0.776441000	-0.818269000
H	2.232972000	-0.749417000	-2.629604000
H	3.822865000	-1.765190000	-1.180936000
C	3.250867000	1.080934000	-1.982934000
C	-2.003491000	-2.177619000	0.528993000
C	-2.616365000	-1.162235000	1.277305000
C	-2.037126000	-3.479545000	1.051463000
C	-3.253355000	-1.378857000	2.504516000
C	-2.647009000	-3.726540000	2.284819000
C	-3.247164000	-2.687787000	3.005659000
O	-2.550166000	0.111017000	0.759628000
H	-1.566991000	-4.296522000	0.501312000
H	-2.657224000	-4.741228000	2.689335000
H	-3.722584000	-2.909080000	3.961973000

C	-3.953297000	-0.176583000	3.150573000
C	-5.299008000	0.051589000	2.407645000
H	-5.813248000	0.935305000	2.816649000
H	-5.135309000	0.213715000	1.331809000
H	-5.953376000	-0.825791000	2.528919000
C	-4.233508000	-0.402177000	4.640961000
H	-3.304647000	-0.562827000	5.209008000
H	-4.761913000	0.459649000	5.072868000
H	-4.885339000	-1.275682000	4.784971000
C	4.442417000	0.387134000	-1.276048000
C	5.519891000	-0.114852000	-2.248660000
C	5.149190000	1.197919000	-0.181269000
C	6.627020000	-0.863207000	-1.508017000
H	5.963247000	0.750173000	-2.775014000
H	5.070037000	-0.769319000	-3.015189000
C	6.272039000	0.387658000	0.461755000
H	5.579758000	2.109755000	-0.633900000
H	4.435030000	1.515517000	0.590818000
H	7.439760000	-1.156520000	-2.191471000
H	6.215060000	-1.792786000	-1.060608000
H	6.831523000	0.986726000	1.197887000
H	5.839706000	-0.481714000	1.003994000
O	7.219429000	-0.062659000	-0.495655000
H	3.431637000	1.332425000	-3.045144000
H	2.947325000	2.008666000	-1.474139000

Profile involving L2 = dppbz
L2Cu-Bpin, G = -3890.238770

Cu	0.703124000	0.058724000	-0.964871000
B	2.717956000	0.193467000	-1.075205000
O	3.593316000	-0.888840000	-1.186987000
O	3.459202000	1.334222000	-0.748792000
C	4.964501000	-0.431394000	-1.092327000
C	4.798064000	0.946856000	-0.357843000
C	5.780226000	-1.473269000	-0.332772000
C	5.495892000	-0.289112000	-2.523156000
C	4.791552000	0.815310000	1.170434000
C	5.779670000	2.032447000	-0.786067000
H	5.841251000	-2.400295000	-0.923364000
H	6.805861000	-1.113484000	-0.153535000
H	5.320216000	-1.719908000	0.633262000
H	6.560221000	-0.009182000	-2.538234000
H	5.381070000	-1.252651000	-3.042027000
H	4.923960000	0.466849000	-3.080734000
H	5.791242000	0.584125000	1.568319000
H	4.454238000	1.767401000	1.607177000
H	4.091750000	0.029611000	1.490978000
H	5.584929000	2.954887000	-0.217452000
H	6.817493000	1.720925000	-0.587889000
H	5.680977000	2.268316000	-1.853712000
C	-1.091375000	0.591323000	1.597251000
C	-0.945160000	-0.820296000	1.586112000
C	-1.009077000	-1.530580000	2.793990000
C	-1.233749000	-0.861391000	4.000431000
H	-1.282821000	-1.427037000	4.934245000
C	-1.389692000	0.528470000	4.011106000
H	-1.562938000	1.055013000	4.952891000
C	-1.312728000	1.249581000	2.816332000
H	-1.416969000	2.336583000	2.835466000

P	-0.903263000	1.491250000	-0.012869000
C	-0.367544000	3.178359000	0.475392000
C	1.018950000	3.411447000	0.515458000
H	1.714820000	2.617275000	0.226653000
C	1.504771000	4.663469000	0.904206000
C	0.616279000	5.691811000	1.238992000
H	0.998811000	6.672201000	1.534792000
C	-0.764423000	5.467254000	1.184571000
H	-1.460981000	6.270415000	1.438897000
C	-1.257113000	4.214700000	0.805238000
H	-2.335683000	4.043930000	0.763068000
C	-2.620135000	1.703744000	-0.629798000
C	-2.789958000	2.357711000	-1.864859000
H	-1.922148000	2.771525000	-2.386954000
C	-4.059296000	2.481111000	-2.432890000
H	-4.177403000	2.996463000	-3.389563000
C	-5.174620000	1.935130000	-1.785336000
H	-6.166624000	2.020119000	-2.236028000
C	-5.011802000	1.274409000	-0.564687000
H	-5.875112000	0.836231000	-0.058091000
C	-3.743831000	1.163539000	0.014817000
H	-3.631949000	0.641757000	0.966589000
P	-0.653987000	-1.625316000	-0.058062000
C	0.086389000	-3.255009000	0.352648000
C	-0.646982000	-4.412082000	0.663046000
H	-1.738740000	-4.390786000	0.657280000
C	0.018275000	-5.601860000	0.976437000
H	-0.559097000	-6.498559000	1.216059000
C	1.417129000	-5.645490000	0.981997000
H	1.933839000	-6.577074000	1.227015000
C	2.151887000	-4.498557000	0.660127000
H	3.244495000	-4.531082000	0.645845000
C	1.492665000	-3.309160000	0.337382000
H	2.066480000	-2.422421000	0.049793000
C	-2.366282000	-1.942253000	-0.647779000
C	-3.422783000	-2.325179000	0.196653000
H	-3.242867000	-2.485260000	1.262424000
C	-4.714337000	-2.475247000	-0.314321000
H	-5.530407000	-2.769821000	0.350428000
C	-4.966029000	-2.232601000	-1.669506000
H	-5.979622000	-2.337666000	-2.064497000
C	-3.922856000	-1.839657000	-2.513839000
H	-4.118359000	-1.632334000	-3.568731000
C	-2.629797000	-1.692535000	-2.004489000
H	-1.818442000	-1.357952000	-2.656784000
H	-0.876918000	-2.614865000	2.793658000

INT0-L2, G = -4277.068050

Cu	0.349835000	-0.301316000	-0.589408000
B	1.820432000	-1.648243000	-0.162015000
O	1.719383000	-2.950540000	0.338568000
O	3.178069000	-1.358645000	-0.351149000
C	2.995259000	-3.624240000	0.227789000
C	3.997856000	-2.415526000	0.201463000
C	3.159560000	-4.577773000	1.406793000
C	2.971637000	-4.414194000	-1.086429000
C	4.434140000	-1.966137000	1.601102000
C	5.221415000	-2.608041000	-0.688899000
H	2.413053000	-5.383958000	1.337987000
H	4.160590000	-5.037588000	1.402290000
H	3.012419000	-4.064613000	2.366073000
H	3.886251000	-5.010613000	-1.223902000

H	2.108841000	-5.097417000	-1.075047000
H	2.856024000	-3.739136000	-1.946967000
H	5.129643000	-2.682322000	2.063978000
H	4.941072000	-0.992948000	1.518069000
H	3.565708000	-1.841274000	2.263503000
H	5.857278000	-1.710296000	-0.643856000
H	5.820959000	-3.469250000	-0.353411000
H	4.937236000	-2.765207000	-1.737696000
C	-1.326291000	0.081020000	2.159413000
C	-2.071286000	-0.929226000	1.503648000
C	-2.870305000	-1.798058000	2.262016000
C	-2.943764000	-1.665676000	3.651785000
H	-3.568591000	-2.350501000	4.230594000
C	-2.214626000	-0.662340000	4.297637000
H	-2.266148000	-0.558040000	5.384283000
C	-1.409233000	0.205193000	3.553955000
H	-0.833279000	0.978708000	4.066394000
P	-0.240699000	1.156961000	1.116393000
C	1.055754000	1.769407000	2.266888000
C	2.264687000	1.054404000	2.304313000
H	2.394043000	0.194024000	1.643941000
C	3.293367000	1.453256000	3.161500000
H	4.228714000	0.888333000	3.182784000
C	3.129402000	2.577553000	3.978767000
H	3.936600000	2.894056000	4.644360000
C	1.932332000	3.301832000	3.936384000
H	1.802561000	4.183359000	4.569534000
C	0.898092000	2.901235000	3.084451000
H	-0.032124000	3.473567000	3.052850000
C	-1.286672000	2.639185000	0.792120000
C	-0.716591000	3.711421000	0.078386000
H	0.342397000	3.685818000	-0.187065000
C	-1.491502000	4.808824000	-0.300173000
H	-1.029546000	5.633464000	-0.849070000
C	-2.856630000	4.846520000	0.011326000
H	-3.467000000	5.699320000	-0.296164000
C	-3.432668000	3.785930000	0.715505000
H	-4.497743000	3.801849000	0.959512000
C	-2.653162000	2.693333000	1.110480000
H	-3.120110000	1.875786000	1.660850000
P	-1.915756000	-1.049093000	-0.340060000
C	-2.383623000	-2.793090000	-0.691606000
C	-3.644805000	-3.186252000	-1.168597000
H	-4.426430000	-2.441811000	-1.334786000
C	-3.909367000	-4.535185000	-1.432365000
H	-4.894002000	-4.830514000	-1.804506000
C	-2.920881000	-5.501205000	-1.218973000
H	-3.129980000	-6.554176000	-1.425252000
C	-1.661419000	-5.112860000	-0.745535000
H	-0.881989000	-5.862130000	-0.581674000
C	-1.386596000	-3.766944000	-0.490123000
H	-0.393637000	-3.471918000	-0.138779000
C	-3.353730000	-0.060535000	-0.941024000
C	-4.529311000	0.148063000	-0.199897000
H	-4.645434000	-0.314156000	0.782558000
C	-5.550659000	0.958616000	-0.703203000
H	-6.456369000	1.118355000	-0.112457000
C	-5.412551000	1.569341000	-1.954469000
H	-6.208666000	2.209342000	-2.343143000
C	-4.249904000	1.361683000	-2.704001000
H	-4.134199000	1.836725000	-3.681453000
C	-3.226221000	0.555001000	-2.198462000
H	-2.316319000	0.407769000	-2.785377000

C	0.379930000	1.307279000	-2.274989000
C	0.630237000	0.077703000	-2.822322000
H	-0.530371000	1.859569000	-2.032225000
C	2.091002000	0.377090000	-3.111270000
H	-0.020000000	-0.726488000	-3.178625000
H	2.827214000	-0.261644000	-2.602993000
H	2.329224000	0.438962000	-4.186623000
C	1.827177000	1.785033000	-2.442557000
C	1.985614000	2.986794000	-3.384190000
C	2.654100000	2.037274000	-1.173729000
C	3.462536000	3.313135000	-3.612550000
H	1.490855000	3.862630000	-2.927768000
H	1.488161000	2.793772000	-4.349879000
C	4.090740000	2.404775000	-1.532006000
H	2.209146000	2.868035000	-0.601417000
H	2.648355000	1.144883000	-0.532486000
O	4.151032000	3.533245000	-2.393226000
H	3.576311000	4.230358000	-4.211386000
H	3.942380000	2.484005000	-4.175241000
H	4.666209000	2.664240000	-0.629860000
H	4.589685000	1.534993000	-2.009394000
H	-3.435652000	-2.588732000	1.763211000

INT0′-L2, G = -4277.068346

Cu	0.554034000	0.135333000	-0.920884000
B	1.975935000	-0.814935000	0.215979000
O	2.022784000	-2.165911000	0.559110000
O	3.005235000	-0.161178000	0.902943000
C	3.190002000	-2.449014000	1.367256000
C	3.536548000	-1.027350000	1.934572000
C	2.814264000	-3.491450000	2.416016000
C	4.266371000	-3.003585000	0.428300000
C	2.781691000	-0.700964000	3.229469000
C	5.024068000	-0.739721000	2.102970000
H	2.590710000	-4.450457000	1.923339000
H	3.643639000	-3.655331000	3.122102000
H	1.922892000	-3.189043000	2.981320000
H	5.170876000	-3.307680000	0.976412000
H	3.863541000	-3.884000000	-0.094556000
H	4.546122000	-2.257341000	-0.329412000
H	3.159406000	-1.286708000	4.081024000
H	2.909089000	0.367066000	3.460489000
H	1.704471000	-0.891387000	3.117752000
H	5.161472000	0.280765000	2.492568000
H	5.481625000	-1.442808000	2.816777000
H	5.560292000	-0.809664000	1.147480000
C	-1.048120000	0.179854000	1.868720000
C	-1.369692000	-1.122917000	1.407363000
C	-1.568299000	-2.149925000	2.339420000
C	-1.455741000	-1.900296000	3.711853000
H	-1.604824000	-2.714829000	4.425185000
C	-1.155840000	-0.613456000	4.166012000
H	-1.068177000	-0.413297000	5.236732000
C	-0.955500000	0.421475000	3.246410000
H	-0.703648000	1.421702000	3.605289000
P	-0.622806000	1.459195000	0.601496000
C	0.251466000	2.772159000	1.550696000
C	1.628042000	2.601266000	1.785237000
H	2.137948000	1.707447000	1.419532000
C	2.342954000	3.565400000	2.501532000
H	3.411496000	3.419086000	2.681179000
C	1.700311000	4.713512000	2.978554000
H	2.263360000	5.469349000	3.532288000

C	0.333564000	4.892251000	2.739625000
H	-0.175369000	5.787008000	3.107586000
C	-0.389920000	3.927077000	2.030958000
H	-1.456809000	4.077926000	1.853992000
C	-2.223726000	2.258533000	0.172254000
C	-2.255871000	3.057938000	-0.983581000
H	-1.352288000	3.180146000	-1.584504000
C	-3.436800000	3.692306000	-1.374395000
H	-3.447329000	4.314085000	-2.273306000
C	-4.606429000	3.517258000	-0.626460000
H	-5.535749000	3.999176000	-0.940497000
C	-4.585658000	2.712416000	0.516305000
H	-5.499442000	2.559161000	1.095487000
C	-3.399734000	2.090959000	0.919153000
H	-3.395674000	1.460535000	1.810640000
P	-1.485388000	-1.365782000	-0.429084000
C	-1.538436000	-3.199059000	-0.649684000
C	-2.658522000	-3.874125000	-1.166314000
H	-3.570528000	-3.325913000	-1.407701000
C	-2.619224000	-5.257643000	-1.374239000
H	-3.499223000	-5.767040000	-1.776038000
C	-1.466175000	-5.985592000	-1.066520000
H	-1.439670000	-7.066832000	-1.225350000
C	-0.344779000	-5.317958000	-0.559781000
H	0.564934000	-5.876434000	-0.321960000
C	-0.372295000	-3.935004000	-0.362086000
H	0.513600000	-3.419662000	0.016779000
C	-3.246948000	-0.881050000	-0.725267000
C	-4.283731000	-1.201418000	0.169017000
H	-4.063819000	-1.756379000	1.083932000
C	-5.597371000	-0.811480000	-0.099783000
H	-6.393873000	-1.065015000	0.604689000
C	-5.891704000	-0.089769000	-1.262303000
H	-6.918152000	0.225178000	-1.466761000
C	-4.867208000	0.238569000	-2.154123000
H	-5.087053000	0.815091000	-3.055825000
C	-3.552801000	-0.154575000	-1.885010000
H	-2.753707000	0.118874000	-2.576154000
C	0.208331000	1.075449000	-2.862744000
C	1.354779000	0.292439000	-2.873250000
H	-0.826102000	0.863195000	-3.139605000
H	1.534444000	-0.740422000	-3.185528000
C	0.987308000	2.386953000	-2.928359000
C	2.291724000	1.515815000	-2.965495000
C	3.057726000	1.557853000	-4.297275000
C	3.292025000	1.753321000	-1.830573000
C	4.233962000	0.578460000	-4.263728000
H	3.444010000	2.579110000	-4.461028000
H	2.390681000	1.313227000	-5.141480000
C	4.440850000	0.755045000	-1.918401000
H	3.694162000	2.778472000	-1.918377000
H	2.796751000	1.659668000	-0.854997000
H	4.840178000	0.659382000	-5.179728000
H	3.848812000	-0.462245000	-4.210175000
H	5.199937000	0.946297000	-1.144872000
H	4.046977000	-0.267756000	-1.751468000
O	5.105050000	0.829967000	-3.174630000
H	0.821624000	2.964360000	-3.854073000
H	0.910206000	3.068499000	-2.065590000
H	-1.802894000	-3.158520000	1.992662000

TS1-L2, G = -4277.052979

Cu	0.320286000	-0.265457000	-0.810008000

B	0.415517000	-2.242320000	-0.255662000
O	-0.706501000	-3.055844000	-0.261718000
O	1.443125000	-2.856555000	0.447683000
C	-0.362224000	-4.340257000	0.318623000
C	0.885680000	-3.965939000	1.196376000
C	-1.563053000	-4.868903000	1.094528000
C	-0.019990000	-5.278140000	-0.842578000
C	0.502265000	-3.428512000	2.581030000
C	1.941734000	-5.056733000	1.322616000
H	-2.383747000	-5.099226000	0.398175000
H	-1.302174000	-5.793394000	1.633100000
H	-1.931826000	-4.132002000	1.820168000
H	0.187720000	-6.301520000	-0.496039000
H	-0.875401000	-5.310019000	-1.533700000
H	0.854529000	-4.909889000	-1.399157000
H	0.119075000	-4.226601000	3.234040000
H	1.392958000	-2.989483000	3.053942000
H	-0.258260000	-2.637454000	2.502587000
H	2.772959000	-4.696773000	1.947987000
H	1.518813000	-5.954773000	1.799464000
H	2.352357000	-5.336696000	0.343724000
C	-0.904954000	0.525344000	2.055744000
C	-2.082425000	0.196311000	1.337343000
C	-3.240797000	-0.151665000	2.046552000
C	-3.233924000	-0.201793000	3.444678000
H	-4.142742000	-0.485969000	3.981093000
C	-2.068013000	0.107359000	4.150179000
H	-2.056376000	0.063032000	5.242005000
C	-0.911740000	0.479276000	3.456618000
H	-0.005867000	0.728936000	4.013006000
P	0.593221000	0.994332000	1.076087000
C	2.020083000	0.768945000	2.214381000
C	2.741680000	-0.434163000	2.133049000
H	2.438418000	-1.203167000	1.419972000
C	3.835759000	-0.654295000	2.975926000
H	4.389694000	-1.594127000	2.905380000
C	4.224791000	0.323766000	3.897381000
H	5.084360000	0.152323000	4.550505000
C	3.514007000	1.527005000	3.976344000
H	3.815502000	2.296322000	4.691909000
C	2.417212000	1.751175000	3.138989000
H	1.872116000	2.695556000	3.203247000
C	0.452384000	2.832110000	0.960333000
C	1.436650000	3.515131000	0.224125000
H	2.261611000	2.959311000	-0.225914000
C	1.365661000	4.898919000	0.056984000
H	2.140519000	5.416019000	-0.514846000
C	0.298305000	5.618704000	0.607558000
H	0.233523000	6.700314000	0.464699000
C	-0.688450000	4.945825000	1.332303000
H	-1.531003000	5.497888000	1.755863000
C	-0.610645000	3.561085000	1.514325000
H	-1.393170000	3.050360000	2.077424000
P	-1.973703000	0.223897000	-0.516734000
C	-3.425013000	-0.747039000	-1.094389000
C	-4.276600000	-0.259467000	-2.101718000
H	-4.133398000	0.745985000	-2.502429000
C	-5.316369000	-1.053317000	-2.597728000
H	-5.970027000	-0.658213000	-3.379734000
C	-5.522422000	-2.341304000	-2.094467000
H	-6.338149000	-2.958382000	-2.479772000
C	-4.673978000	-2.836611000	-1.097058000
H	-4.821941000	-3.844951000	-0.701110000

C	-3.628603000	-2.051952000	-0.606420000
H	-2.954366000	-2.457496000	0.146352000
C	-2.424097000	1.969107000	-0.909327000
C	-3.574603000	2.592163000	-0.396627000
H	-4.265206000	2.029405000	0.236824000
C	-3.835166000	3.932574000	-0.688052000
H	-4.729123000	4.414246000	-0.283318000
C	-2.947281000	4.660977000	-1.490657000
H	-3.147730000	5.713068000	-1.709312000
C	-1.800507000	4.046503000	-2.000735000
H	-1.098105000	4.616458000	-2.613492000
C	-1.537990000	2.704415000	-1.709297000
H	-0.627664000	2.216184000	-2.071110000
C	1.157737000	-0.180451000	-2.697236000
C	1.112731000	-1.548774000	-2.162473000
H	0.544539000	0.189958000	-3.526768000
C	2.648895000	-1.625952000	-2.141283000
H	0.495002000	-2.358114000	-2.574297000
H	3.115485000	-1.793744000	-1.159347000
H	3.039367000	-2.377988000	-2.846037000
C	2.696977000	-0.163737000	-2.707956000
C	3.317481000	-0.058791000	-4.109361000
C	3.402226000	0.850745000	-1.796917000
C	4.843088000	-0.151102000	-4.058475000
H	3.033275000	0.913865000	-4.550111000
H	2.913317000	-0.845837000	-4.768860000
C	4.920218000	0.703217000	-1.863978000
H	3.120166000	1.870025000	-2.113712000
H	3.055147000	0.718184000	-0.759633000
O	5.403153000	0.824419000	-3.193956000
H	5.287712000	0.016506000	-5.052485000
H	5.143388000	-1.168274000	-3.725891000
H	5.426215000	1.482779000	-1.272679000
H	5.217204000	-0.280807000	-1.441367000
H	-4.155899000	-0.402131000	1.506286000

TS1'-L2, G = -4277.049670

Cu	-0.133237000	0.105599000	-1.015285000
B	-1.787902000	1.149834000	-0.353580000
O	-1.978625000	2.517686000	-0.503937000
O	-2.501976000	0.692803000	0.743606000
C	-2.966514000	2.975429000	0.456020000
C	-2.908752000	1.837118000	1.538455000
C	-2.568065000	4.359830000	0.957010000
C	-4.309642000	3.045871000	-0.275910000
C	-1.810719000	2.065395000	2.585435000
C	-4.234076000	1.521173000	2.220923000
H	-2.621662000	5.083269000	0.129294000
H	-3.254570000	4.694885000	1.750007000
H	-1.544490000	4.374184000	1.352125000
H	-5.102223000	3.454738000	0.367980000
H	-4.201729000	3.699526000	-1.154229000
H	-4.622187000	2.052980000	-0.628310000
H	-2.064087000	2.893387000	3.263306000
H	-1.687040000	1.153499000	3.187497000
H	-0.843654000	2.278158000	2.107456000
H	-4.093862000	0.699765000	2.940221000
H	-4.603959000	2.399480000	2.772583000
H	-5.001865000	1.210721000	1.501081000
P	0.348153000	-1.525997000	0.609086000
C	-0.832476000	-2.645240000	1.465878000
C	-1.889099000	-2.074744000	2.201288000

H	-1.959253000	-0.991258000	2.290059000
C	-2.873178000	-2.883366000	2.771448000
H	-3.685958000	-2.423553000	3.339860000
C	-2.830231000	-4.272967000	2.602539000
H	-3.605416000	-4.904956000	3.043200000
C	-1.793709000	-4.846004000	1.860203000
H	-1.754447000	-5.929040000	1.717742000
C	-0.798911000	-4.039543000	1.295688000
H	0.002906000	-4.503771000	0.718695000
C	1.743789000	-2.625121000	0.119180000
C	2.059855000	-2.701903000	-1.244966000
H	1.476590000	-2.105804000	-1.953760000
C	3.129134000	-3.494223000	-1.674853000
H	3.377408000	-3.539054000	-2.737921000
C	3.888868000	-4.209389000	-0.744875000
H	4.729852000	-4.821981000	-1.080222000
C	3.580519000	-4.132478000	0.619814000
H	4.178782000	-4.685022000	1.348970000
C	2.513387000	-3.341840000	1.051414000
H	2.281490000	-3.278172000	2.117603000
P	1.725187000	1.147881000	-0.223614000
C	2.063124000	2.956634000	-0.250012000
C	3.364788000	3.487129000	-0.216902000
H	4.229209000	2.819707000	-0.200827000
C	3.560089000	4.871563000	-0.203288000
H	4.575699000	5.274805000	-0.176760000
C	2.460733000	5.737641000	-0.223482000
H	2.616113000	6.819557000	-0.212887000
C	1.164277000	5.213802000	-0.266888000
H	0.300895000	5.883873000	-0.292903000
C	0.963181000	3.830004000	-0.287162000
H	-0.049286000	3.425649000	-0.341998000
C	3.298566000	0.422619000	-0.855221000
C	4.327763000	-0.050646000	-0.027963000
H	4.231792000	0.009575000	1.058122000
C	5.477517000	-0.616972000	-0.589706000
H	6.267651000	-0.996043000	0.063376000
C	5.613562000	-0.703311000	-1.978116000
H	6.511411000	-1.148782000	-2.413995000
C	4.590143000	-0.230459000	-2.808428000
H	4.685357000	-0.306245000	-3.894660000
C	3.434566000	0.317336000	-2.249814000
H	2.614231000	0.642422000	-2.895743000
C	-0.568408000	-0.369339000	-2.938005000
C	-1.744962000	0.250530000	-2.263905000
H	-0.029004000	0.125303000	-3.754252000
H	-2.174515000	1.204368000	-2.596379000
C	-1.287438000	-1.711565000	-3.107020000
C	1.723235000	0.710305000	1.577273000
C	1.094556000	-0.501916000	1.959164000
C	2.275795000	1.540410000	2.562986000
C	1.043723000	-0.852401000	3.315657000
C	2.205858000	1.185564000	3.914187000
C	1.591737000	-0.011984000	4.290143000
H	2.756878000	2.478109000	2.277634000
H	2.631197000	1.847992000	4.672110000
H	1.534730000	-0.294057000	5.344435000
C	-2.565773000	-1.069170000	-2.488316000
C	-3.654103000	-0.847844000	-3.553583000
C	-3.190520000	-1.802566000	-1.298706000
C	-4.912080000	-0.224678000	-2.957461000
H	-3.925794000	-1.827639000	-3.985040000
H	-3.266013000	-0.222472000	-4.375469000

C	-4.514356000	-1.184957000	-0.848048000
H	-3.366648000	-2.851162000	-1.599250000
H	-2.489258000	-1.815854000	-0.454586000
H	-5.703595000	-0.124549000	-3.717083000
H	-4.686287000	0.794170000	-2.577156000
H	-5.004874000	-1.821942000	-0.095211000
H	-4.317610000	-0.207547000	-0.377855000
O	-5.440224000	-1.031001000	-1.918171000
H	-1.445695000	-2.051690000	-4.146953000
H	-0.880181000	-2.565259000	-2.534422000
H	0.558328000	-1.782339000	3.619197000

INT1-L2, G = -4277.103542

Cu	-0.218413000	0.066808000	-0.845467000
B	-2.610105000	2.255646000	-1.096497000
O	-2.266525000	3.588622000	-1.052176000
O	-2.916016000	1.773433000	0.164869000
C	-2.526749000	4.090808000	0.280374000
C	-2.501087000	2.767845000	1.136260000
C	-1.453461000	5.113704000	0.637066000
C	-3.907187000	4.754418000	0.248486000
C	-1.086767000	2.394118000	1.588598000
C	-3.461796000	2.739764000	2.318852000
H	-1.569305000	6.007258000	0.005300000
H	-1.542563000	5.423739000	1.689739000
H	-0.446346000	4.712798000	0.466473000
H	-4.153251000	5.226947000	1.210817000
H	-3.910529000	5.529990000	-0.531651000
H	-4.691090000	4.022201000	0.004587000
H	-0.691252000	3.102037000	2.330618000
H	-1.092392000	1.395620000	2.043063000
H	-0.407337000	2.364663000	0.725603000
H	-3.360473000	1.785991000	2.858575000
H	-3.232891000	3.554633000	3.022797000
H	-4.505946000	2.836122000	1.994314000
C	1.367702000	-0.441277000	2.106773000
C	2.192649000	0.543940000	1.508145000
C	3.133249000	1.223072000	2.298619000
H	3.775925000	1.983024000	1.849624000
C	3.246618000	0.949212000	3.664198000
H	3.979965000	1.490723000	4.266827000
C	2.420568000	-0.011513000	4.256715000
H	2.502341000	-0.223349000	5.325641000
C	1.489420000	-0.705545000	3.479725000
H	0.850248000	-1.458192000	3.946491000
P	0.166135000	-1.313548000	1.008379000
C	-1.117003000	-1.976832000	2.141005000
C	-2.287335000	-1.215998000	2.308589000
H	-2.411513000	-0.285460000	1.751409000
C	-3.304633000	-1.663502000	3.155373000
H	-4.211554000	-1.065414000	3.275484000
C	-3.169090000	-2.882034000	3.830475000
H	-3.968562000	-3.236883000	4.485836000
C	-2.013350000	-3.651878000	3.656052000
H	-1.908068000	-4.607477000	4.176016000
C	-0.990412000	-3.203345000	2.814629000
H	-0.093251000	-3.811983000	2.678288000
C	1.084129000	-2.798009000	0.430349000
C	0.484163000	-3.568344000	-0.583528000
H	-0.506016000	-3.299282000	-0.960150000
C	1.152216000	-4.668522000	-1.123880000
H	0.674345000	-5.259331000	-1.909350000

C	2.435046000	-5.001650000	-0.673223000
H	2.964163000	-5.854133000	-1.106429000
C	3.041336000	-4.233551000	0.324779000
H	4.047063000	-4.481459000	0.672951000
C	2.369691000	-3.138853000	0.878375000
H	2.858699000	-2.541318000	1.650059000
P	1.953642000	0.851476000	-0.305973000
C	2.631158000	2.535016000	-0.591844000
C	3.417360000	2.809140000	-1.724863000
H	3.729090000	1.995925000	-2.384102000
C	3.807333000	4.119798000	-2.017619000
H	4.420138000	4.314643000	-2.901458000
C	3.419672000	5.175160000	-1.186076000
H	3.726184000	6.198418000	-1.416435000
C	2.636654000	4.912172000	-0.056301000
H	2.327935000	5.728839000	0.601170000
C	2.241224000	3.605058000	0.236518000
H	1.632393000	3.418610000	1.122939000
C	3.182626000	-0.285076000	-1.074655000
C	4.527943000	-0.321165000	-0.670149000
H	4.888183000	0.367283000	0.098174000
C	5.408764000	-1.241780000	-1.241409000
H	6.453364000	-1.265788000	-0.920669000
C	4.952313000	-2.137847000	-2.216787000
H	5.641040000	-2.864043000	-2.656005000
C	3.615255000	-2.107961000	-2.621989000
H	3.251424000	-2.812606000	-3.373107000
C	2.733452000	-1.183503000	-2.054588000
H	1.682903000	-1.169414000	-2.356908000
C	-1.559153000	0.187023000	-2.316647000
C	-2.569216000	1.417375000	-2.411168000
H	-1.051193000	0.108207000	-3.298176000
C	-3.721312000	0.414597000	-2.702922000
H	-2.361087000	2.102850000	-3.250630000
H	-4.639230000	0.512098000	-2.096030000
H	-4.020120000	0.419126000	-3.764463000
C	-2.789352000	-0.783750000	-2.348308000
C	-2.705388000	-1.914663000	-3.378479000
C	-3.148992000	-1.408949000	-0.993454000
C	-3.939249000	-2.815228000	-3.347669000
H	-1.808706000	-2.523257000	-3.154594000
H	-2.571061000	-1.502249000	-4.393723000
C	-4.361008000	-2.328510000	-1.084728000
H	-2.286828000	-1.992611000	-0.633708000
H	-3.322451000	-0.616917000	-0.252020000
O	-4.173668000	-3.350217000	-2.054806000
H	-3.827936000	-3.674624000	-4.028767000
H	-4.827941000	-2.237562000	-3.684229000
H	-4.546576000	-2.836316000	-0.124467000
H	-5.270420000	-1.738981000	-1.331886000

INT1'-L2, G = -4277.104479

Cu	-0.002849000	-0.093843000	-1.045292000
B	2.905283000	-1.389554000	-0.690862000
O	2.859787000	-2.759150000	-0.510908000
O	2.976461000	-0.732930000	0.523410000
C	3.166294000	-3.053172000	0.873130000
C	2.789068000	-1.700468000	1.588738000
C	2.355360000	-4.264356000	1.320266000
C	4.664865000	-3.368327000	0.938097000
C	1.319809000	-1.635835000	2.005833000
C	3.686094000	-1.316607000	2.760488000

H	2.677775000	-5.152904000	0.756574000
H	2.514813000	-4.462164000	2.391535000
H	1.281863000	-4.124820000	1.144616000
H	4.975348000	-3.665054000	1.950620000
H	4.882942000	-4.198232000	0.249693000
H	5.265550000	-2.501106000	0.625577000
H	1.087867000	-2.348499000	2.809578000
H	1.084373000	-0.627587000	2.368558000
H	0.662647000	-1.842403000	1.150812000
H	3.345323000	-0.363136000	3.191646000
H	3.640415000	-2.083255000	3.549154000
H	4.731040000	-1.192831000	2.448337000
P	-0.485409000	1.506596000	0.625283000
C	0.597612000	2.661765000	1.560228000
C	1.899417000	2.221447000	1.861592000
H	2.238567000	1.243329000	1.513771000
C	2.774236000	3.045107000	2.575509000
H	3.784010000	2.692571000	2.800909000
C	2.365287000	4.320166000	2.982547000
H	3.052910000	4.967169000	3.533116000
C	1.076665000	4.769354000	2.672668000
H	0.754946000	5.767121000	2.982093000
C	0.195051000	3.945463000	1.965245000
H	-0.808719000	4.305926000	1.728623000
C	-1.770483000	2.575497000	-0.138757000
C	-1.527353000	3.016032000	-1.452259000
H	-0.599602000	2.726149000	-1.954085000
C	-2.471971000	3.797332000	-2.121275000
H	-2.274646000	4.133001000	-3.142367000
C	-3.675851000	4.131856000	-1.490543000
H	-4.421673000	4.731935000	-2.017876000
C	-3.929760000	3.685306000	-0.189351000
H	-4.874800000	3.933338000	0.300210000
C	-2.981535000	2.911118000	0.486278000
H	-3.193189000	2.553244000	1.496500000
P	-1.963960000	-1.070186000	-0.225308000
C	-2.104711000	-2.898388000	-0.307134000
C	-3.308560000	-3.603341000	-0.138643000
H	-4.240964000	-3.062194000	0.040180000
C	-3.319648000	-4.999423000	-0.210721000
H	-4.258989000	-5.542126000	-0.077757000
C	-2.132846000	-5.700915000	-0.456390000
H	-2.145438000	-6.792327000	-0.512984000
C	-0.934691000	-5.003391000	-0.643231000
H	-0.007753000	-5.543724000	-0.849880000
C	-0.921192000	-3.606890000	-0.578779000
H	0.009317000	-3.059054000	-0.753401000
C	-3.553716000	-0.437070000	-0.890879000
C	-4.702087000	-0.232124000	-0.108597000
H	-4.683744000	-0.448005000	0.961754000
C	-5.871450000	0.264968000	-0.691868000
H	-6.759190000	0.423839000	-0.074410000
C	-5.903866000	0.564685000	-2.057815000
H	-6.817553000	0.957969000	-2.510573000
C	-4.760514000	0.371295000	-2.841178000
H	-4.776380000	0.616664000	-3.905957000
C	-3.589370000	-0.121249000	-2.260202000
H	-2.689213000	-0.250597000	-2.867814000
C	1.267593000	-0.513593000	-2.522913000
C	2.783336000	-0.773265000	-2.116631000
H	0.868680000	-1.333158000	-3.148634000
H	3.259656000	-1.468575000	-2.835473000
C	1.761700000	0.706624000	-3.351301000

C	-2.022264000	-0.639454000	1.570094000
C	-1.384399000	0.570859000	1.947250000
C	-2.610566000	-1.446280000	2.554031000
C	-1.374906000	0.949831000	3.297647000
C	-2.587112000	-1.060121000	3.897928000
C	-1.974630000	0.140928000	4.267926000
H	-3.082283000	-2.390423000	2.273759000
H	-3.045242000	-1.700564000	4.655575000
H	-1.953296000	0.446324000	5.316981000
C	3.104114000	0.696615000	-2.575482000
C	4.371388000	0.869632000	-3.421256000
C	3.133841000	1.750191000	-1.457284000
C	5.626297000	0.894097000	-2.551523000
H	4.306705000	1.827448000	-3.969910000
H	4.448770000	0.066539000	-4.174195000
C	4.464416000	1.776669000	-0.707295000
H	2.970959000	2.742710000	-1.915755000
H	2.304201000	1.573322000	-0.760576000
H	6.530790000	1.068710000	-3.156306000
H	5.747201000	-0.088795000	-2.045296000
H	4.498391000	2.624149000	-0.004371000
H	4.582360000	0.848791000	-0.118871000
O	5.570721000	1.934065000	-1.589515000
H	1.926244000	0.476094000	-4.422690000
H	1.187406000	1.650944000	-3.300710000
H	-0.881181000	1.876002000	3.598871000

10. NMR Spectra

^{1}H-NMR (300 MHz, $CDCl_3$) of compound **SI-1**

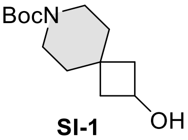

SI-1

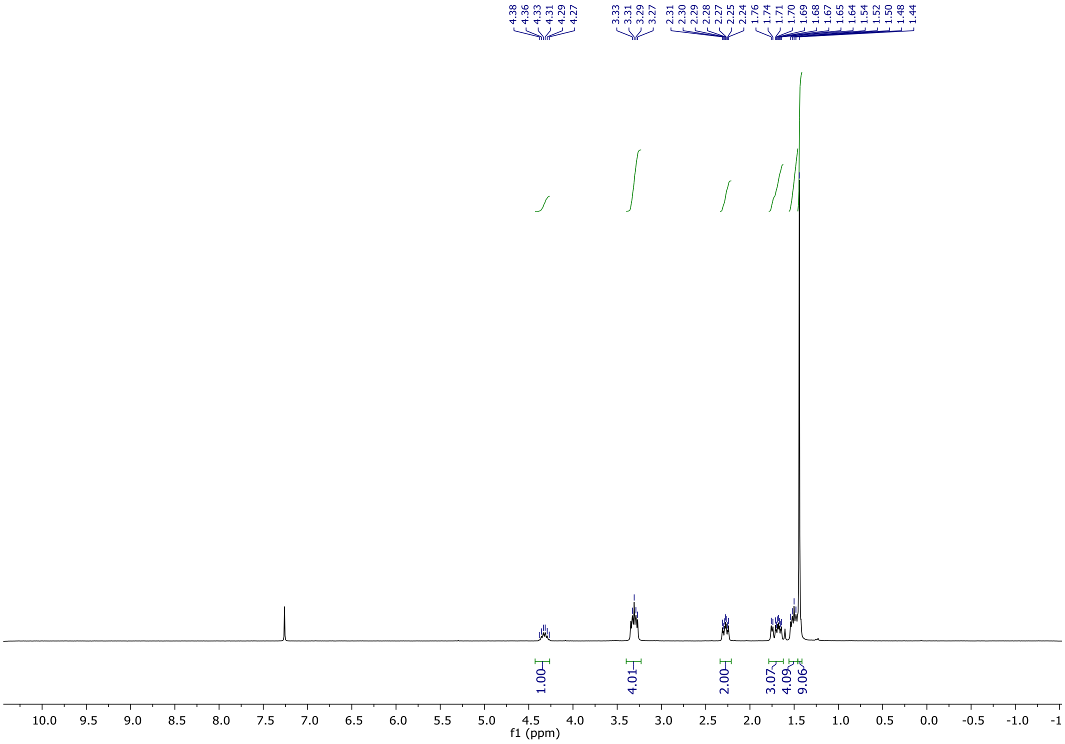

^{13}C-NMR (75 MHz, $CDCl_3$) of compound **SI-1**

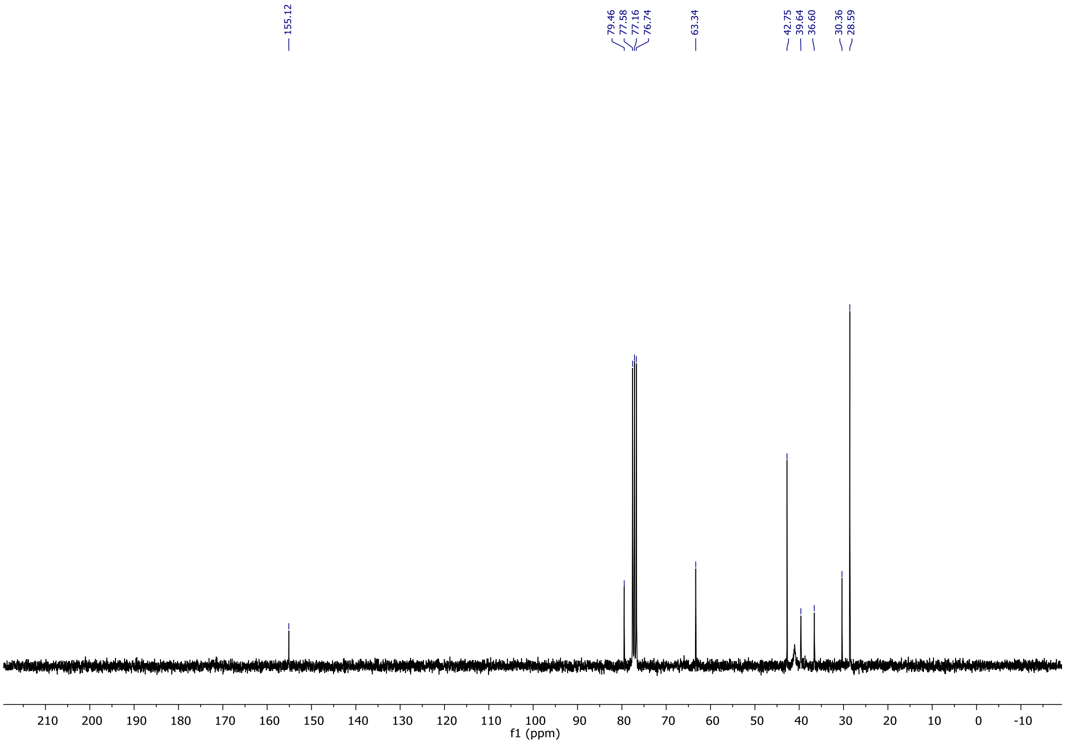

^{1}H-NMR (300 MHz, $CDCl_3$) of compound **2a**

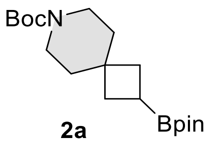

2a

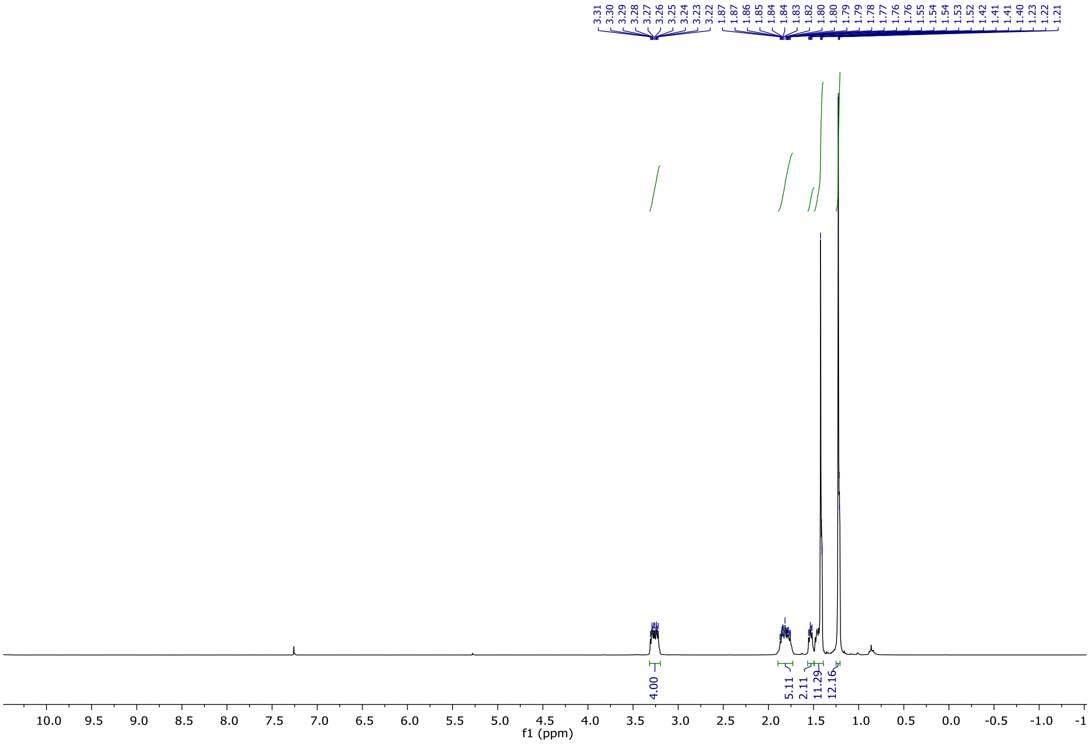

^{13}C-NMR (75 MHz, $CDCl_3$) of compound **2a**

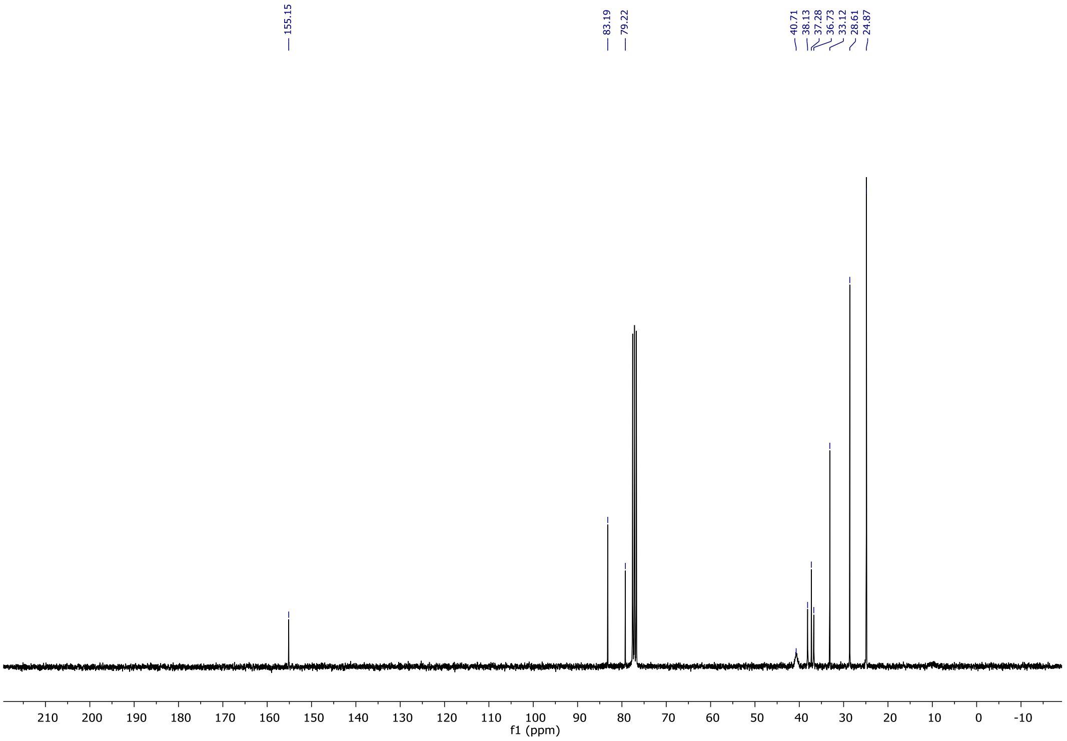

^{11}B-NMR (96 MHz, $CDCl_3$) of compound **2a**

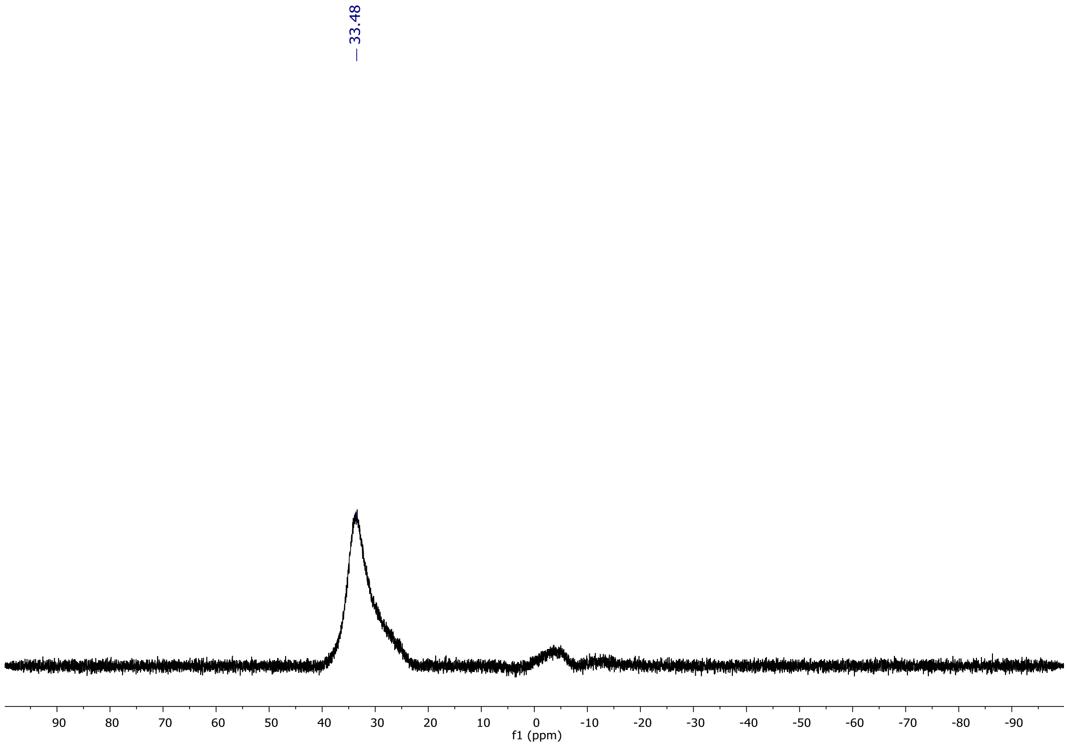

^{1}H-NMR (300 MHz, $CDCl_3$) of compound **2b**

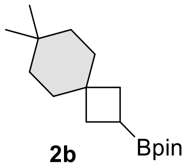

2b

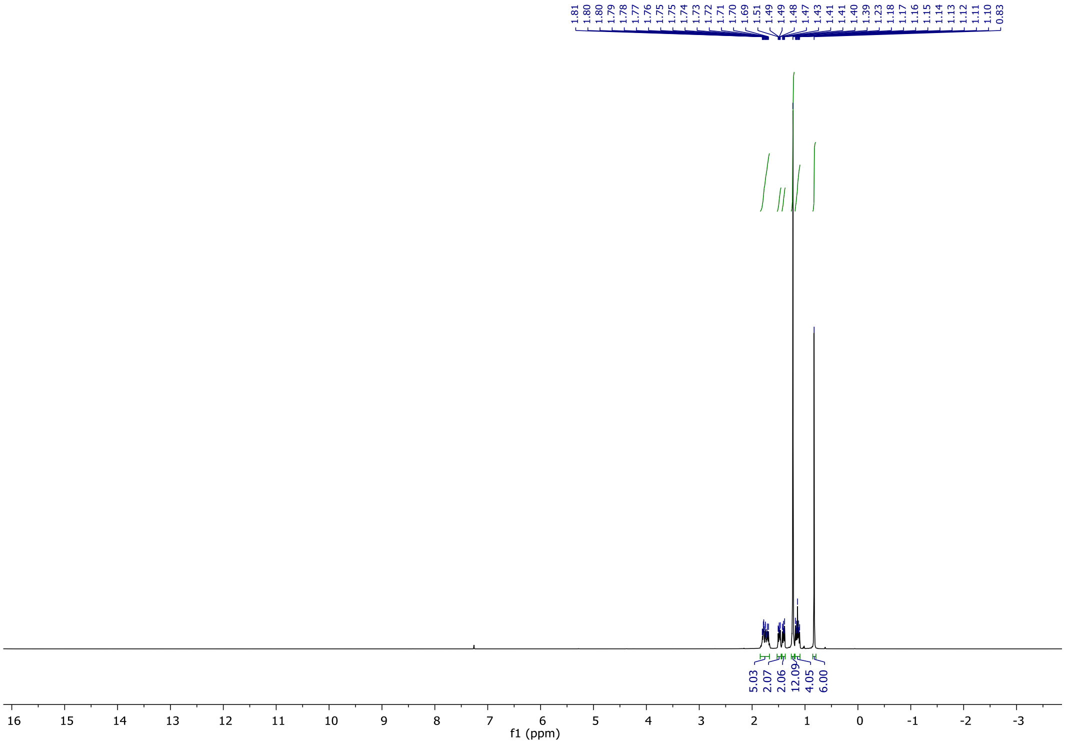

^{13}C-NMR (75 MHz, CDCl3) of compound **2b**

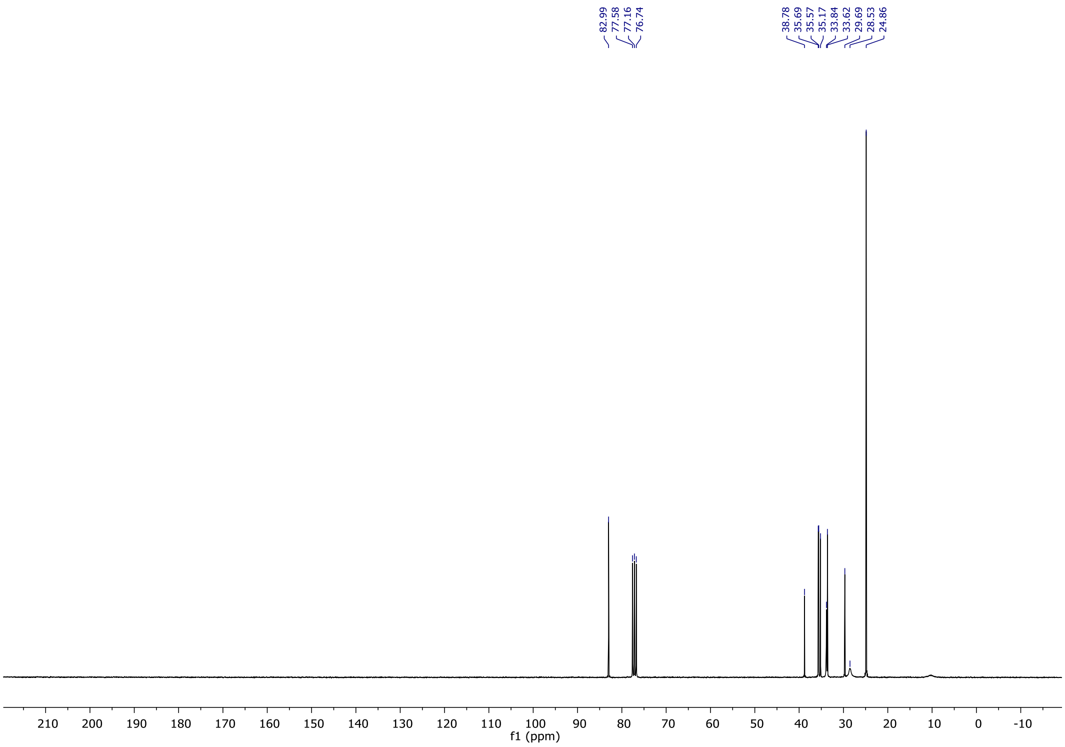

^{11}**B-NMR** (96 MHz, $CDCl_3$) of compound **2b**

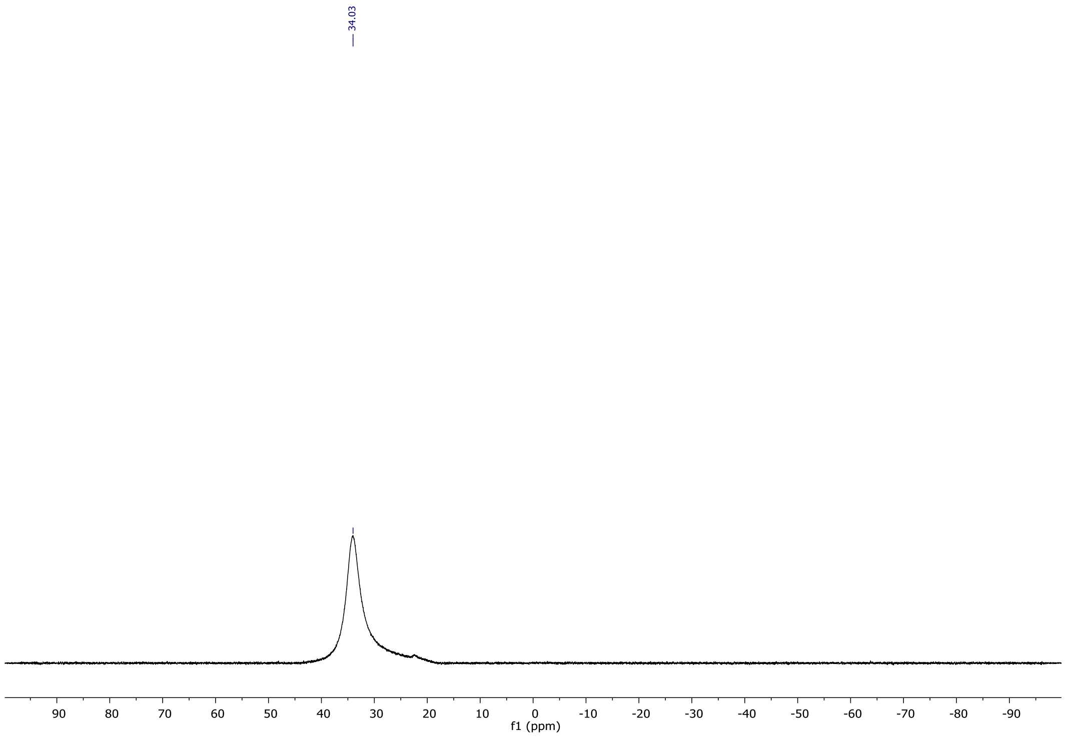

^{1}H-NMR (300 MHz, $CDCl_3$) of compound **2c**

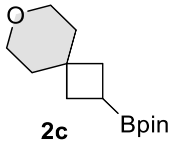

2c

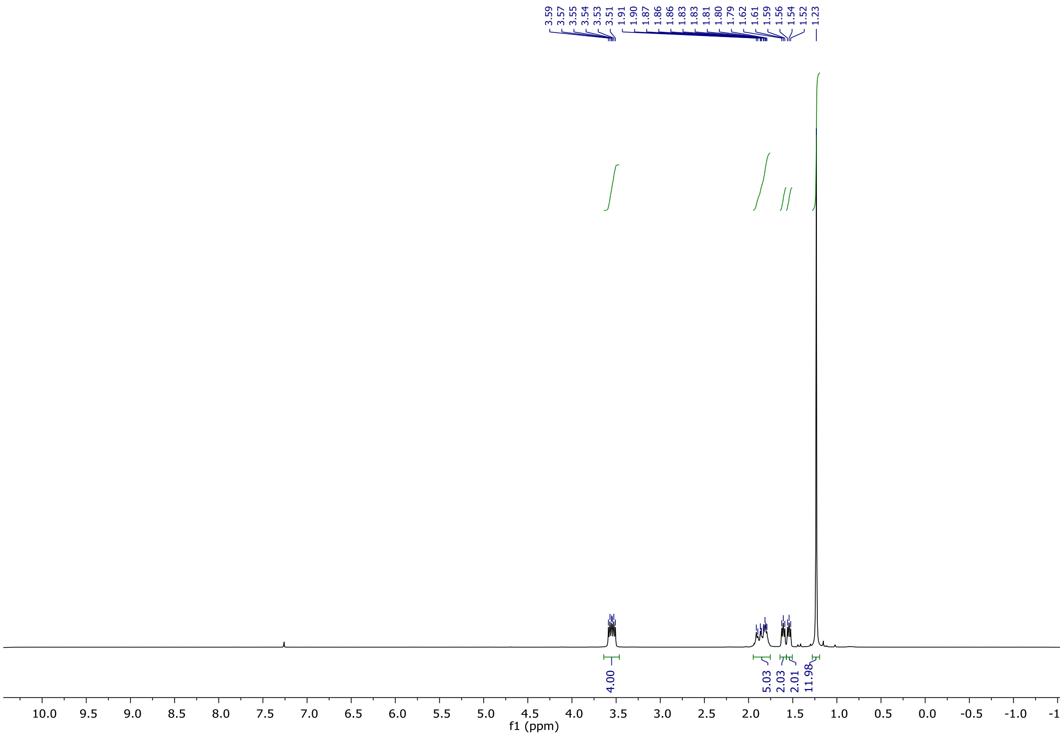

^{13}C-NMR (75 MHz, $CDCl_3$) of compound **2c**

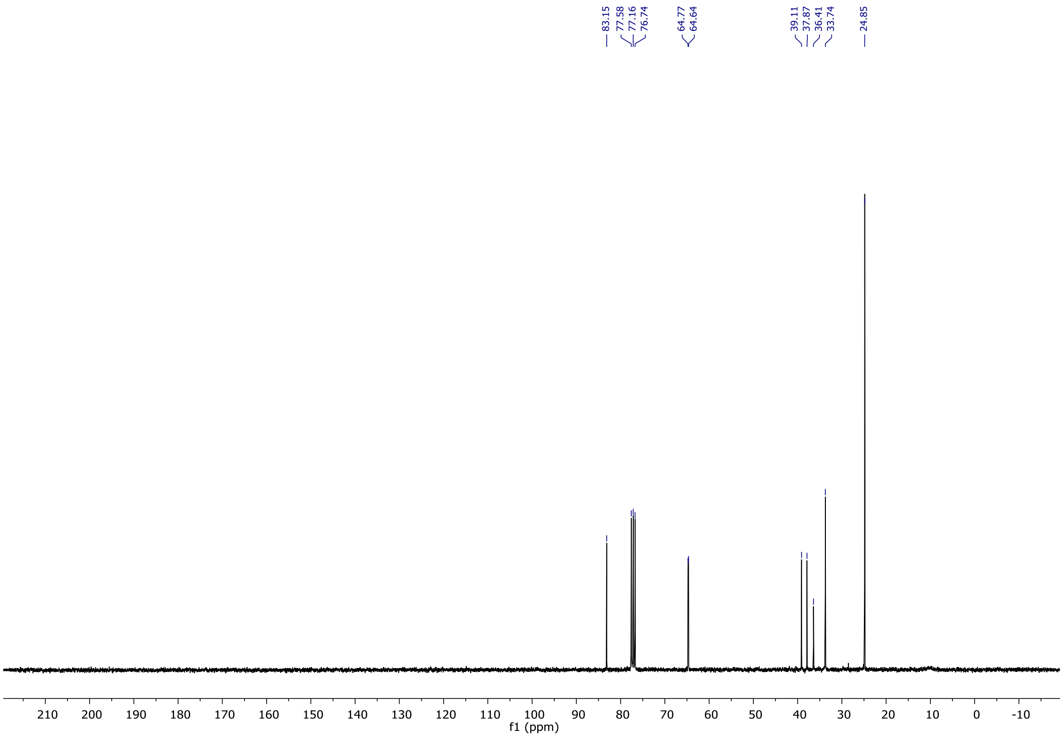

^{11}**B-NMR** (96 MHz, $CDCl_3$) of compound **2c**

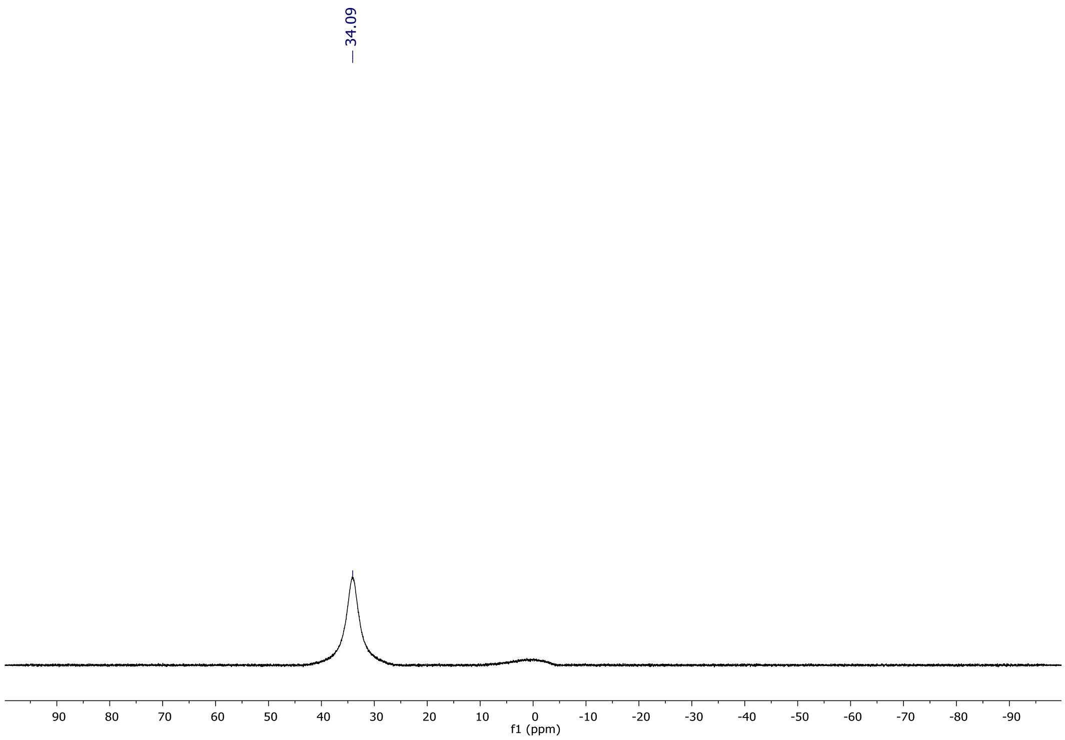

¹H-NMR (300 MHz, $CDCl_3$) of compound **2d**

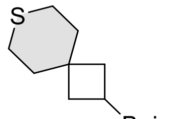

2d

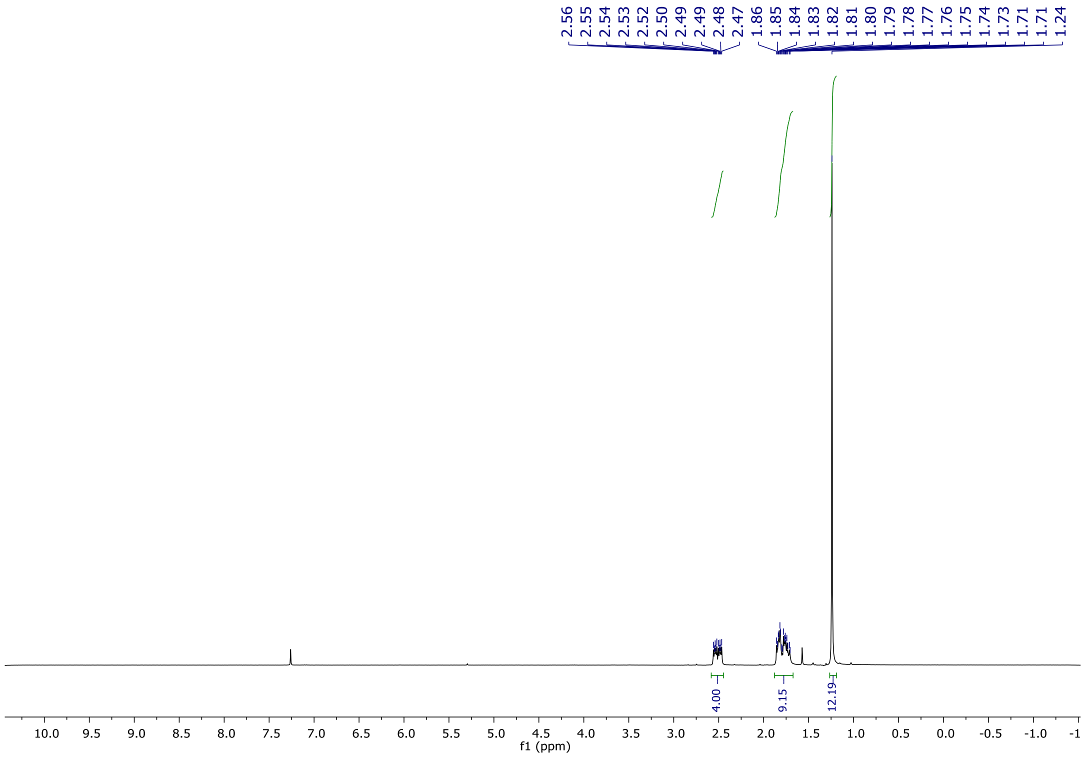

¹³C-NMR (75 MHz, $CDCl_3$) of compound **2d**

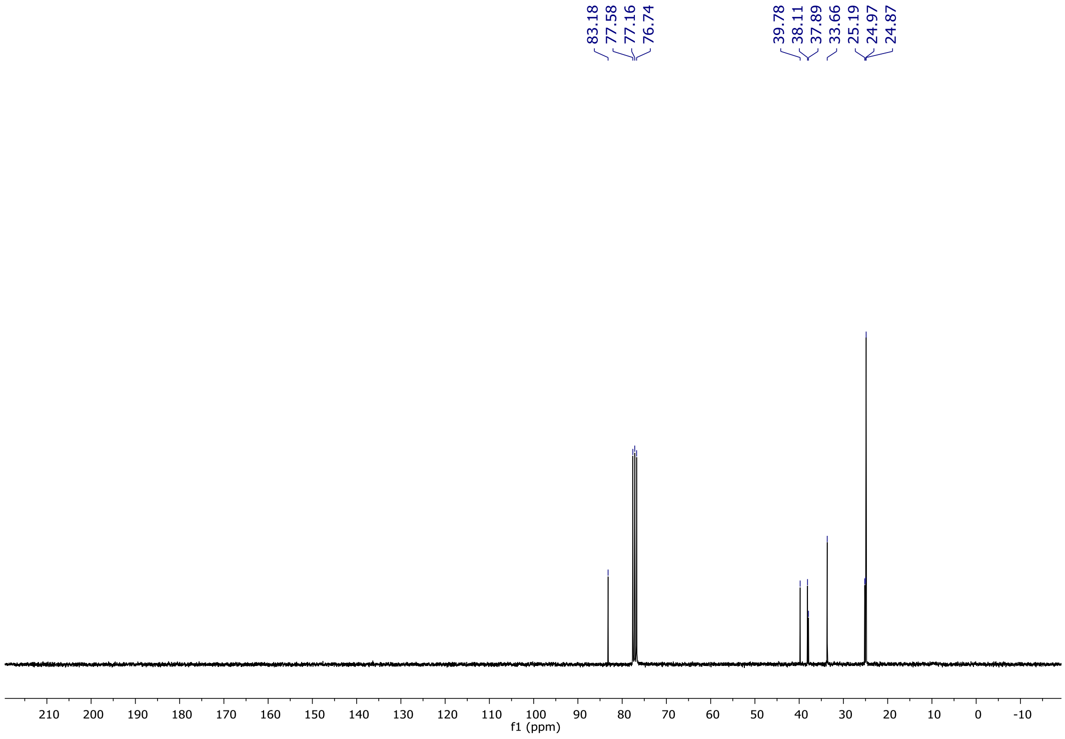

^{11}B-NMR (96 MHz, $CDCl_3$) of compound **2d**

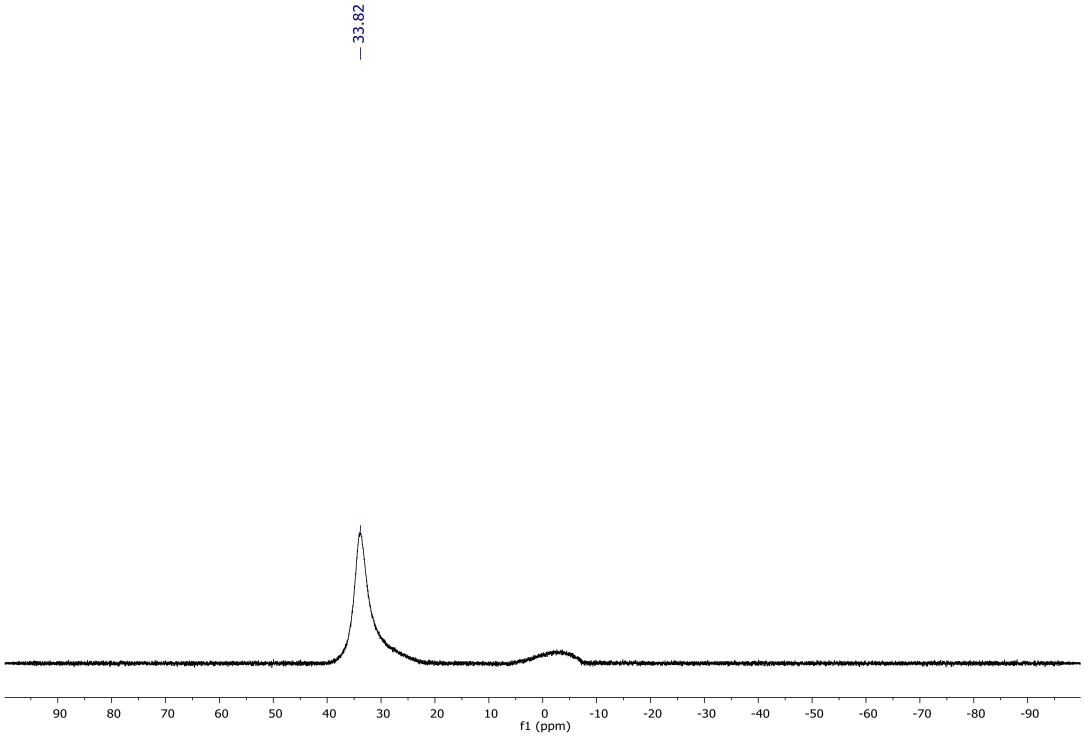

^{1}H-NMR (300 MHz, $CDCl_3$) of compound **2e**

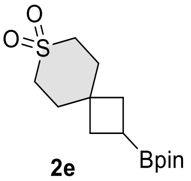

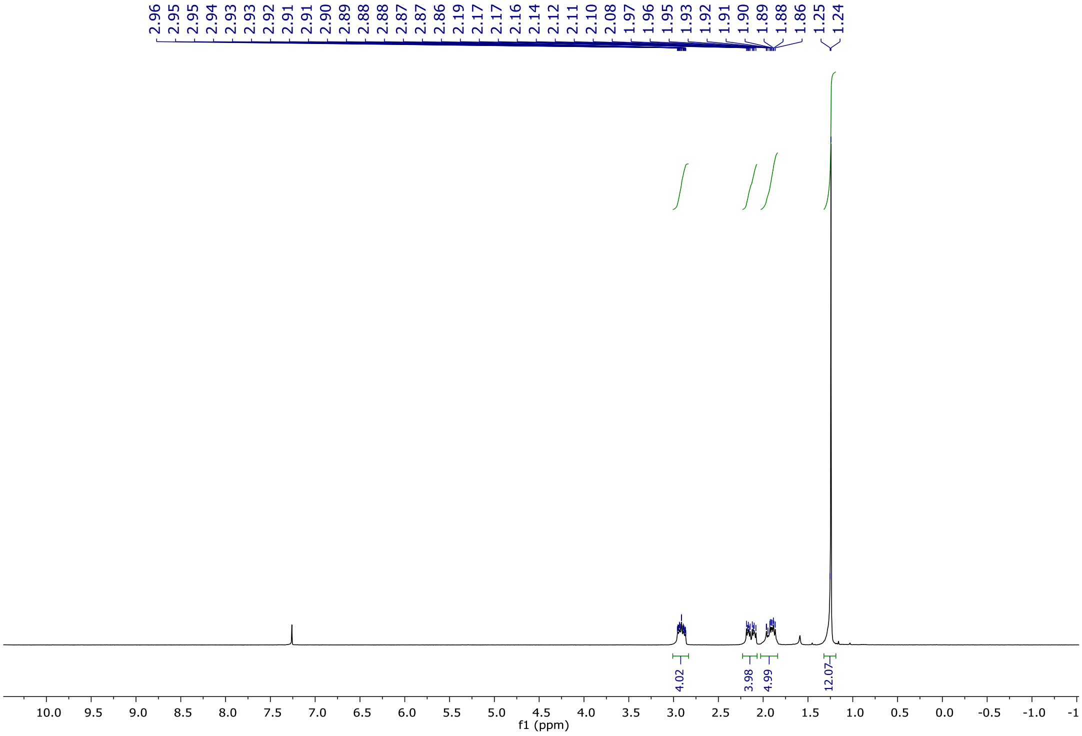

^{13}C-NMR (75 MHz, $CDCl_3$) of compound **2e**

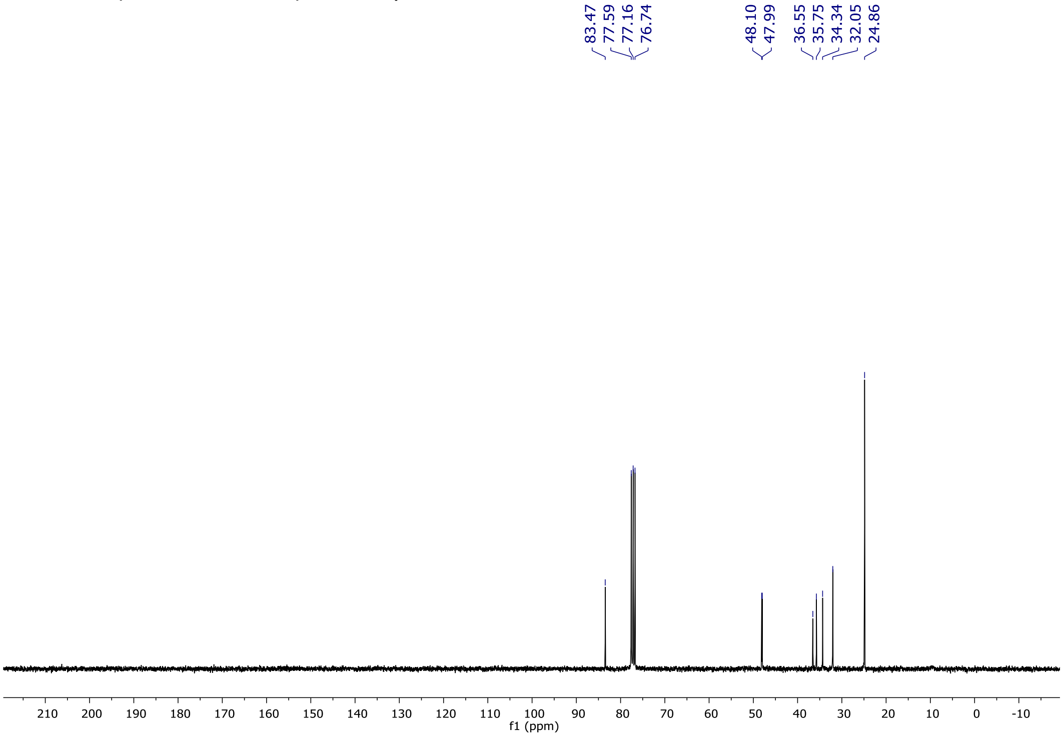

^{1}H-NMR (300 MHz, $CDCl_3$) of compound **2f**

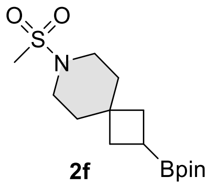

2f

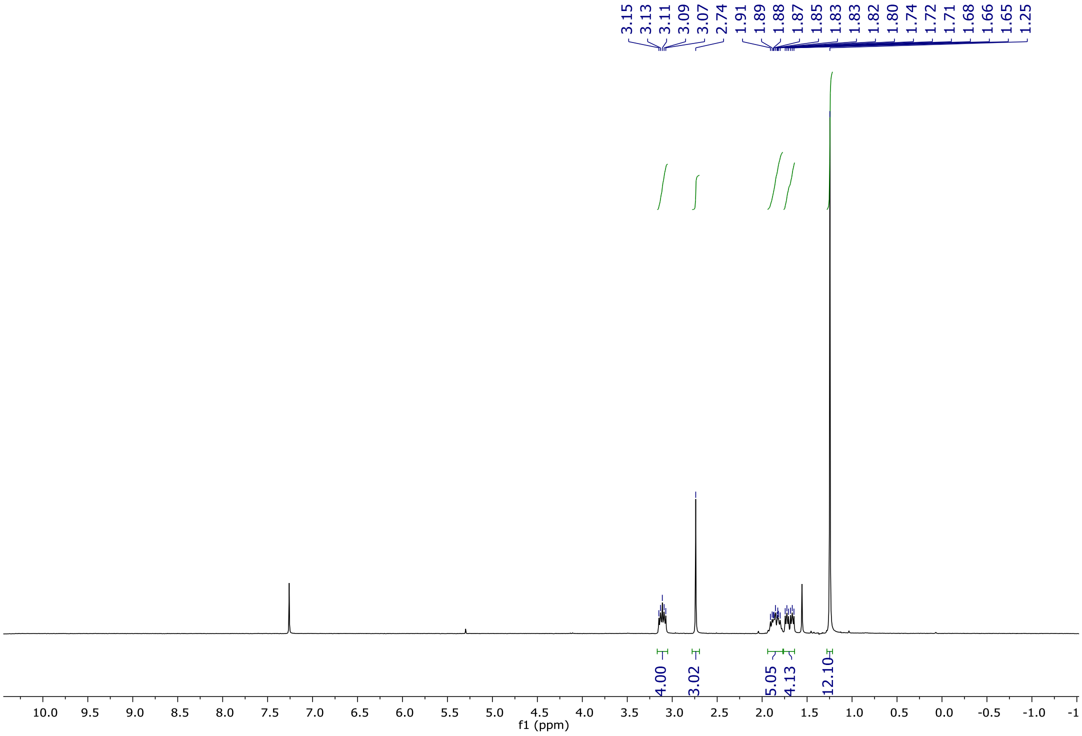

^{13}C-NMR (75 MHz, $CDCl_3$) of compound **2f**

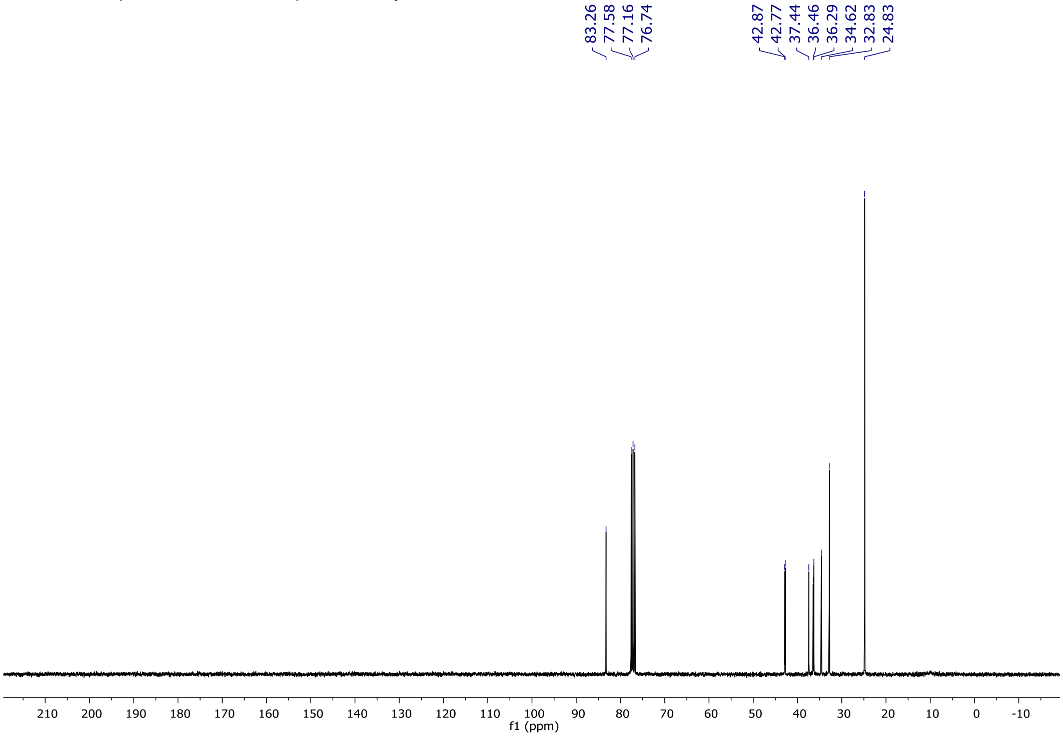

^{11}B-NMR (96 MHz, $CDCl_3$) of compound **2f**

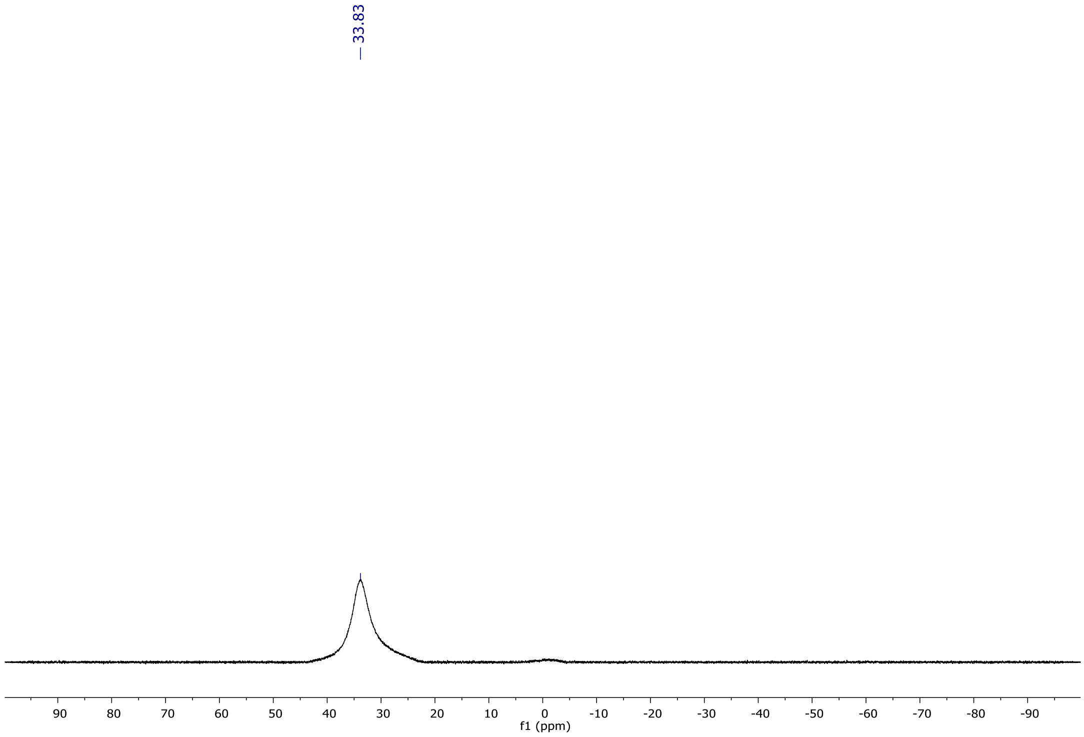

^{1}H-NMR (300 MHz, $CDCl_3$) of compound **2g**

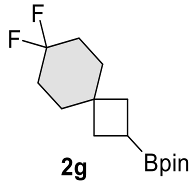

2g

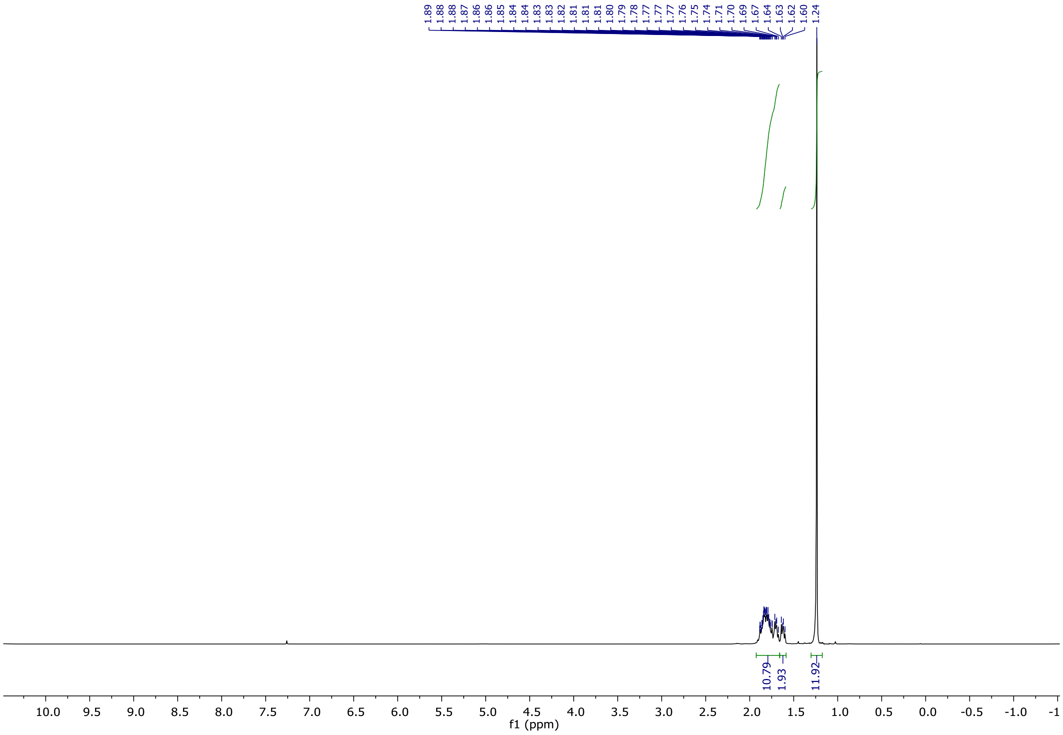

^{13}C-NMR (75 MHz, $CDCl_3$) of compound **2g**

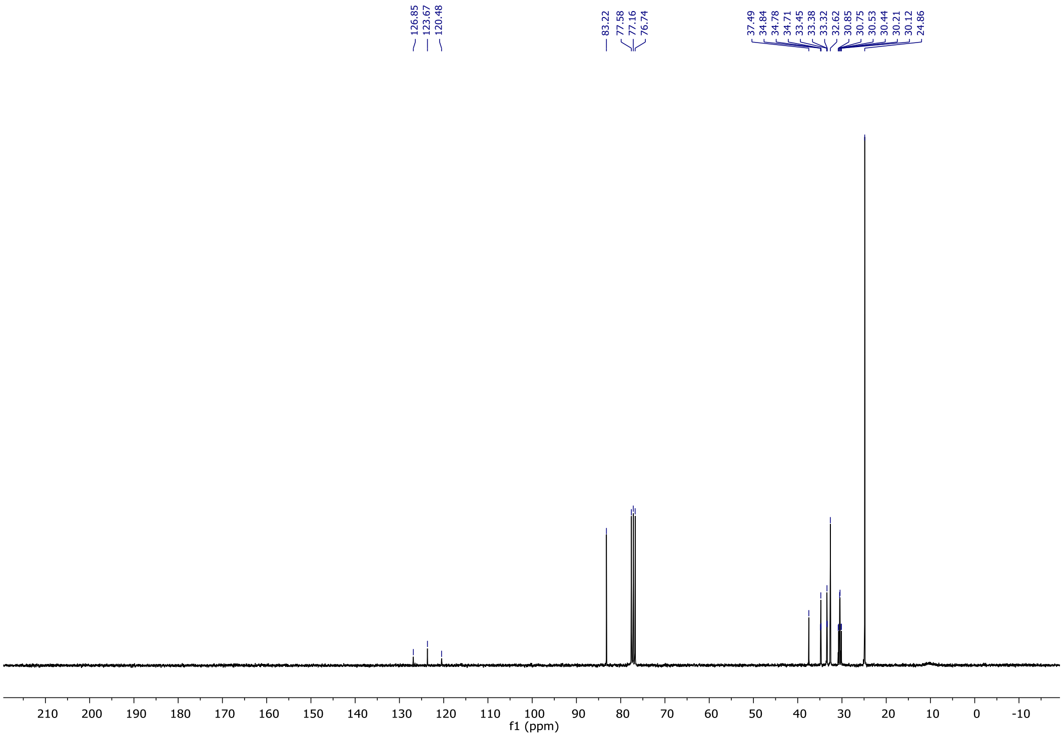

^{19}F-NMR (282 MHz, $CDCl_3$) of compound **2g**

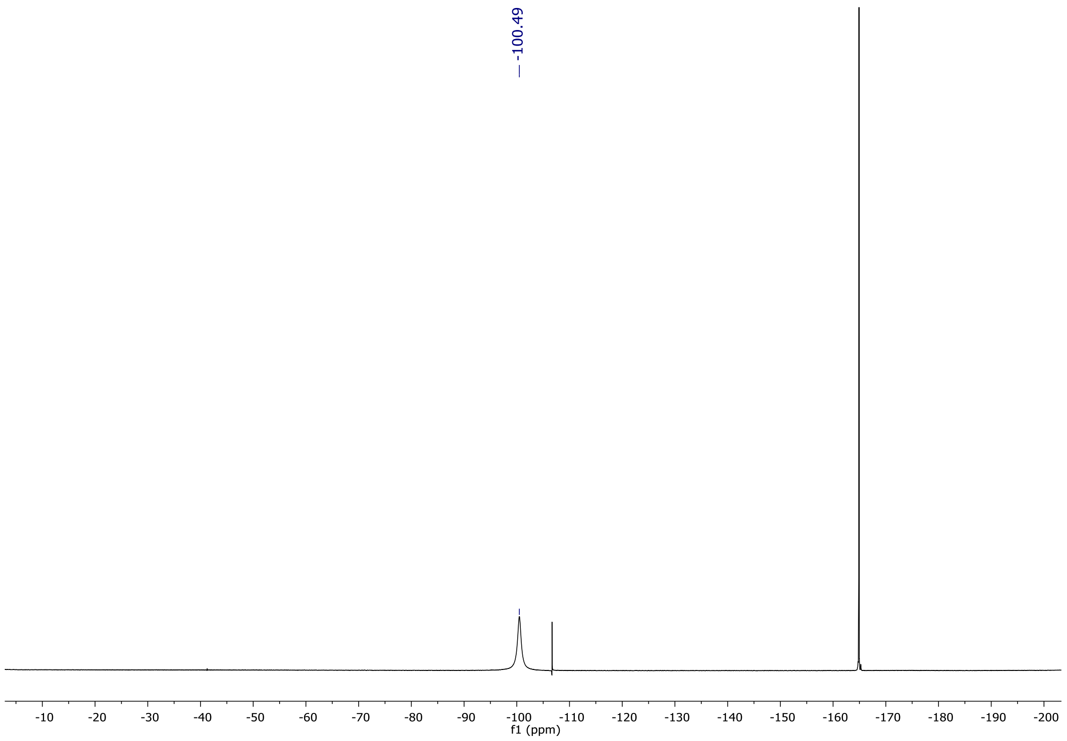

^{11}B-NMR (96 MHz, $CDCl_3$) of compound **2g**

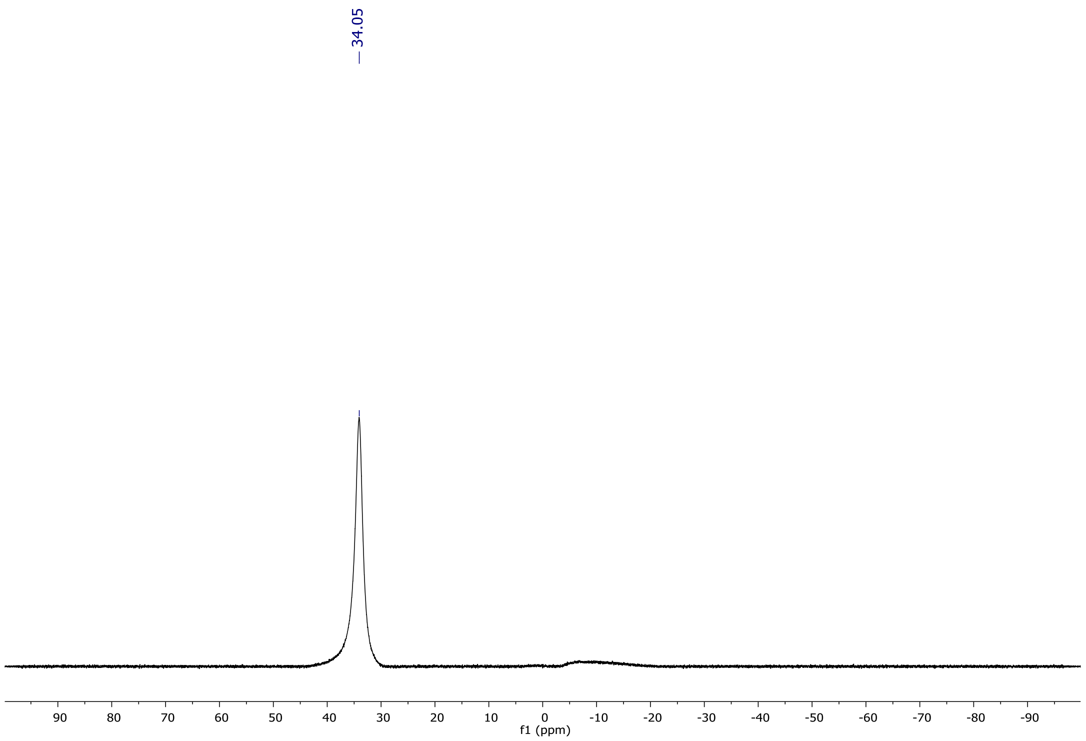

^{1}H-NMR (300 MHz, $CDCl_3$) of compound **2h**

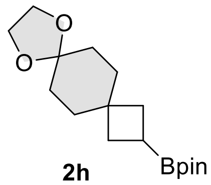

2h

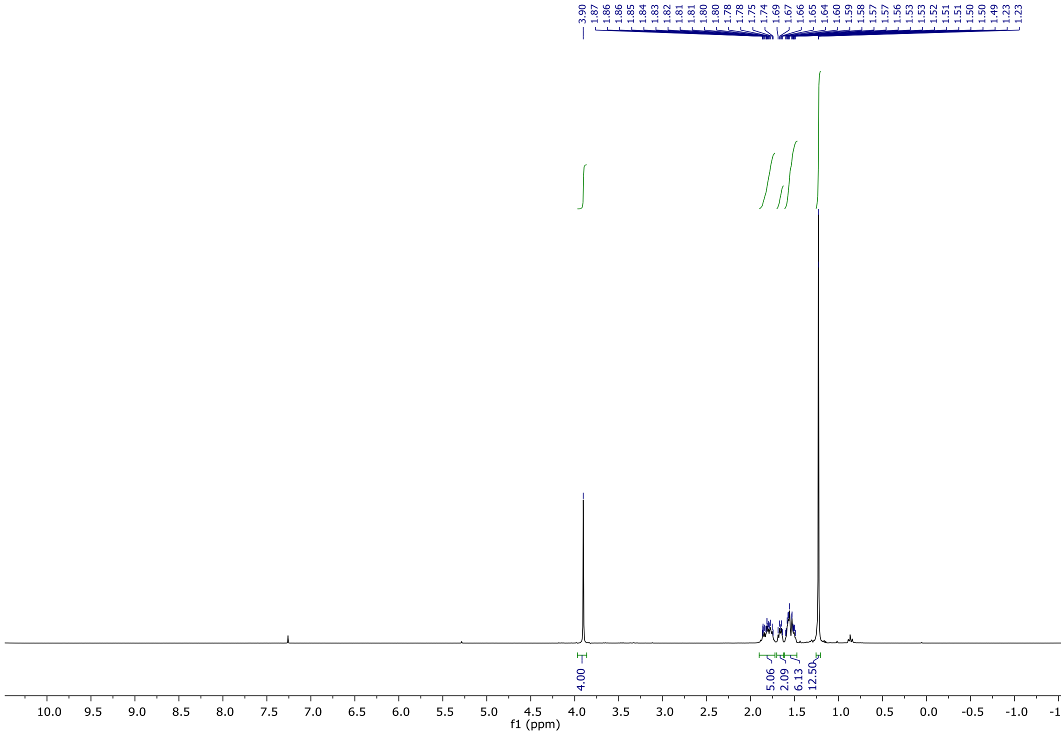

^{13}C-NMR (75 MHz, $CDCl_3$) of compound **2h**

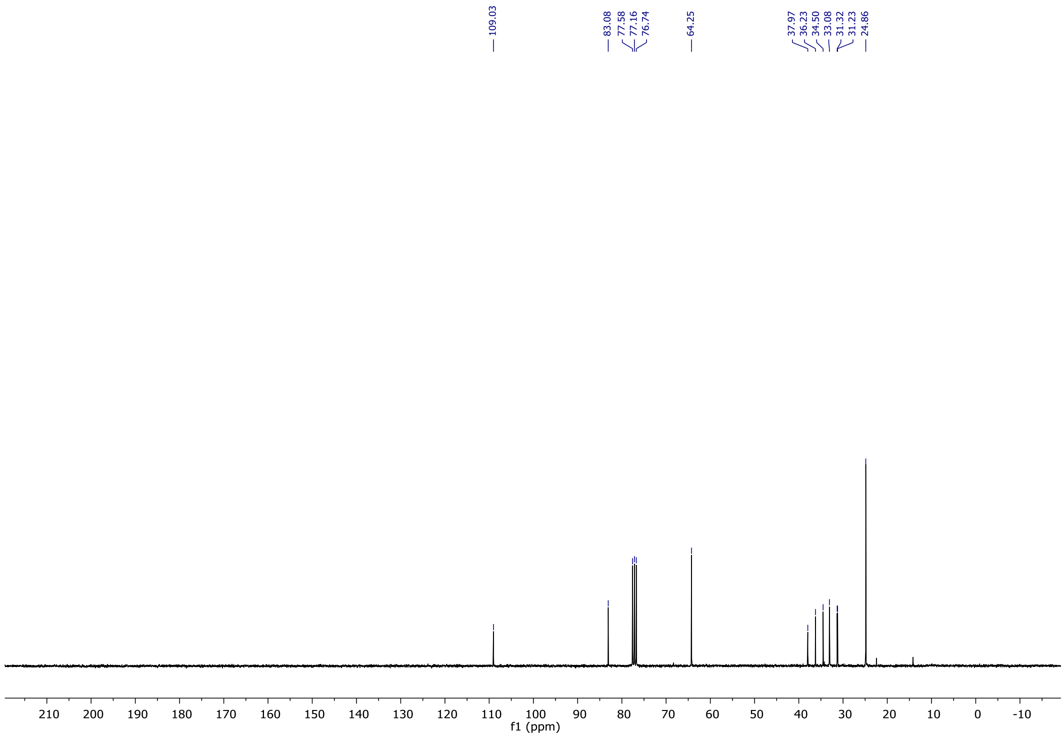

^{11}B-NMR (96 MHz, $CDCl_3$) of compound **2h**

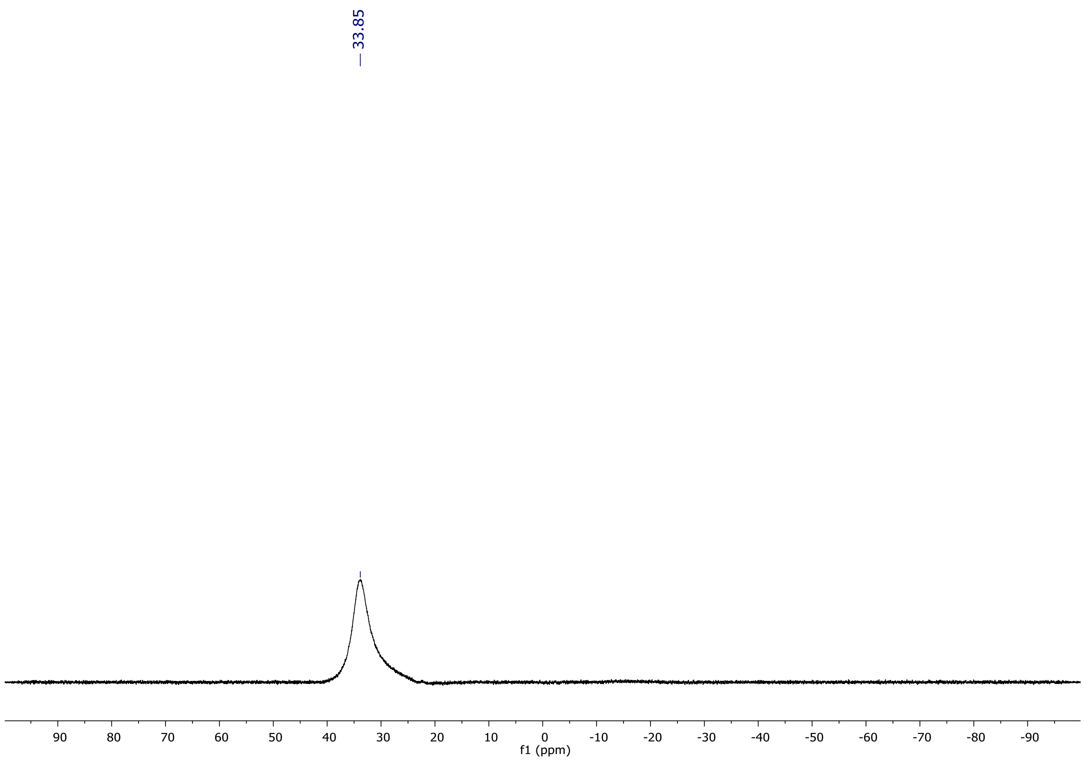

^{1}H-NMR (300 MHz, $CDCl_3$) of compound **2i**

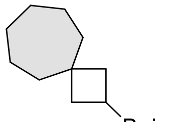

2i

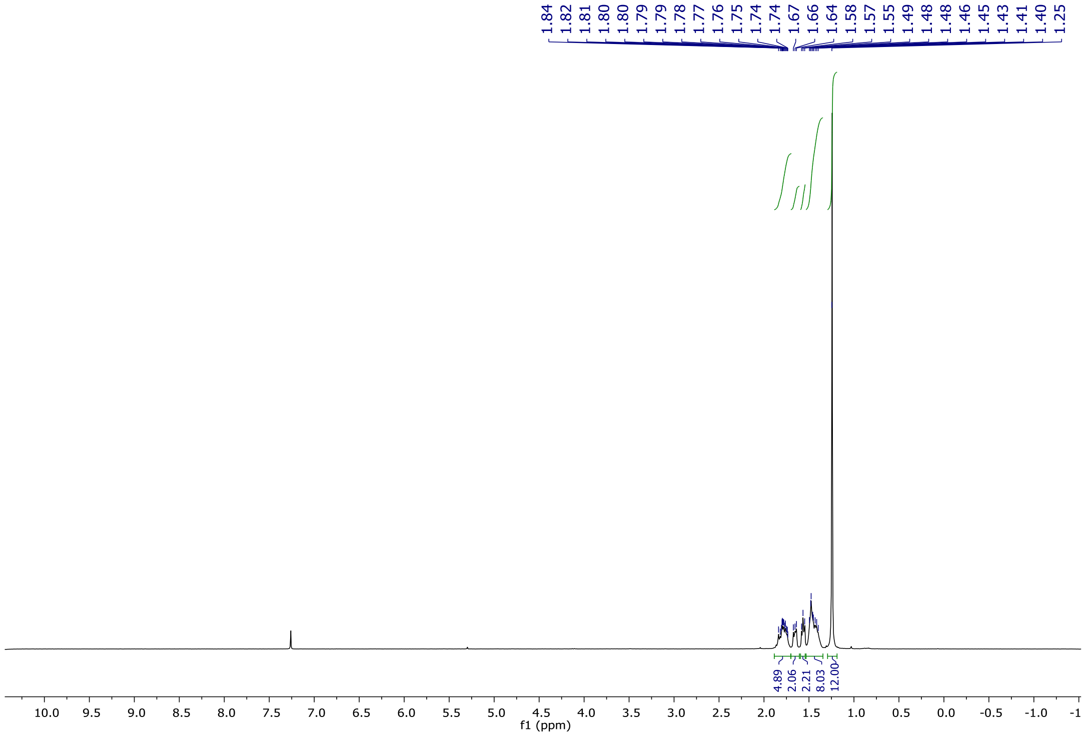

^{13}C-NMR (75 MHz, $CDCl_3$) of compound **2i**

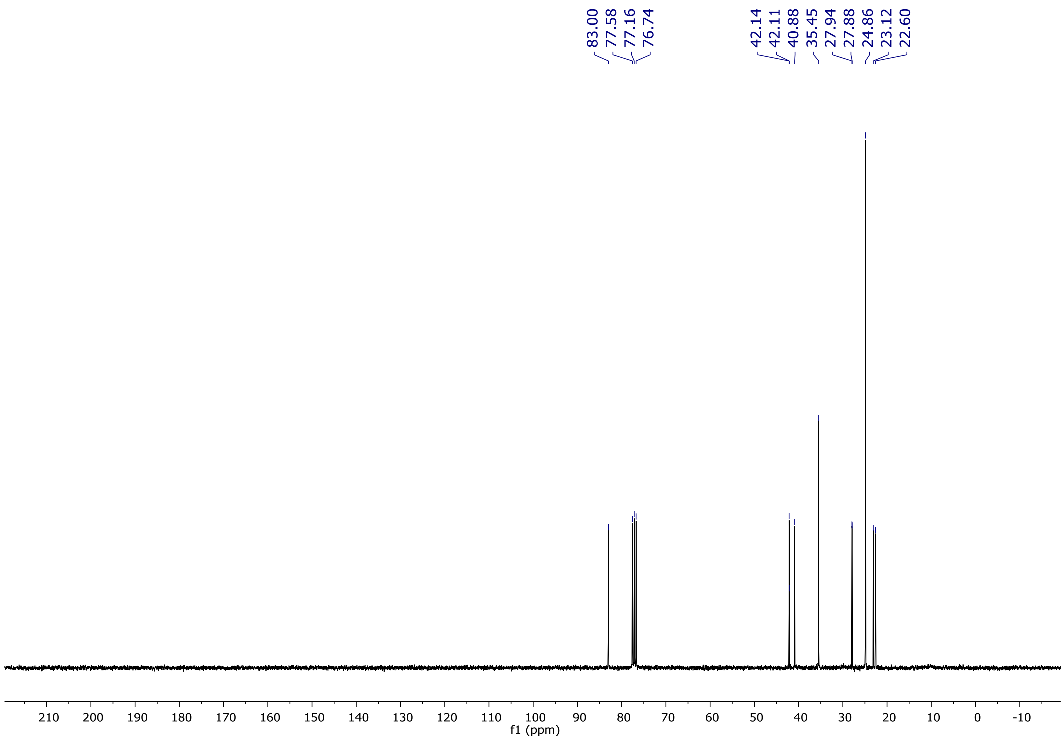

^{1}H-NMR (300 MHz, $CDCl_3$) of compound **2j**

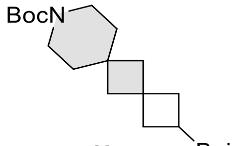

2j

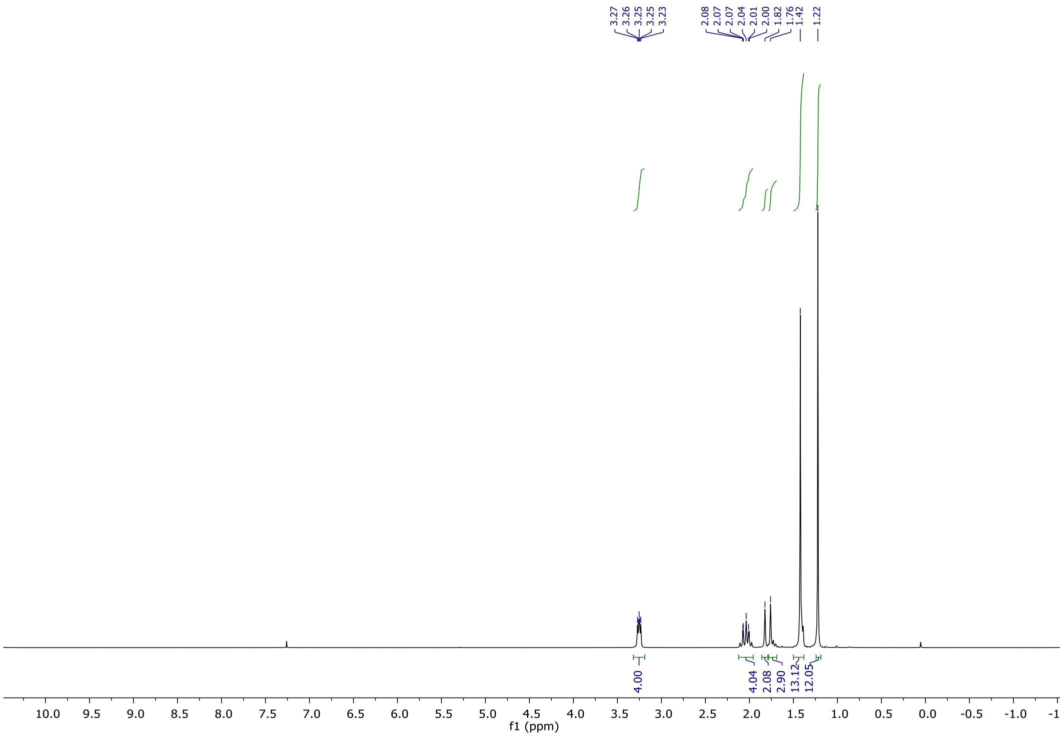

^{13}C-NMR (75 MHz, $CDCl_3$) of compound **2j**

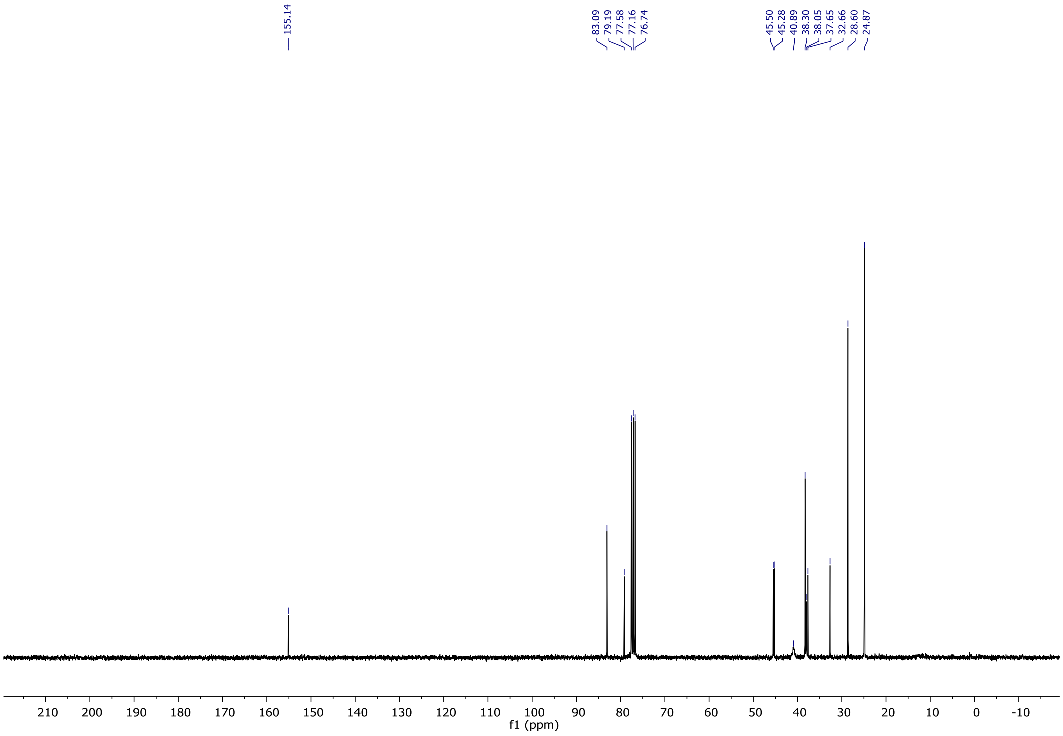

^{11}**B-NMR** (96 MHz, $CDCl_3$) of compound **2j**

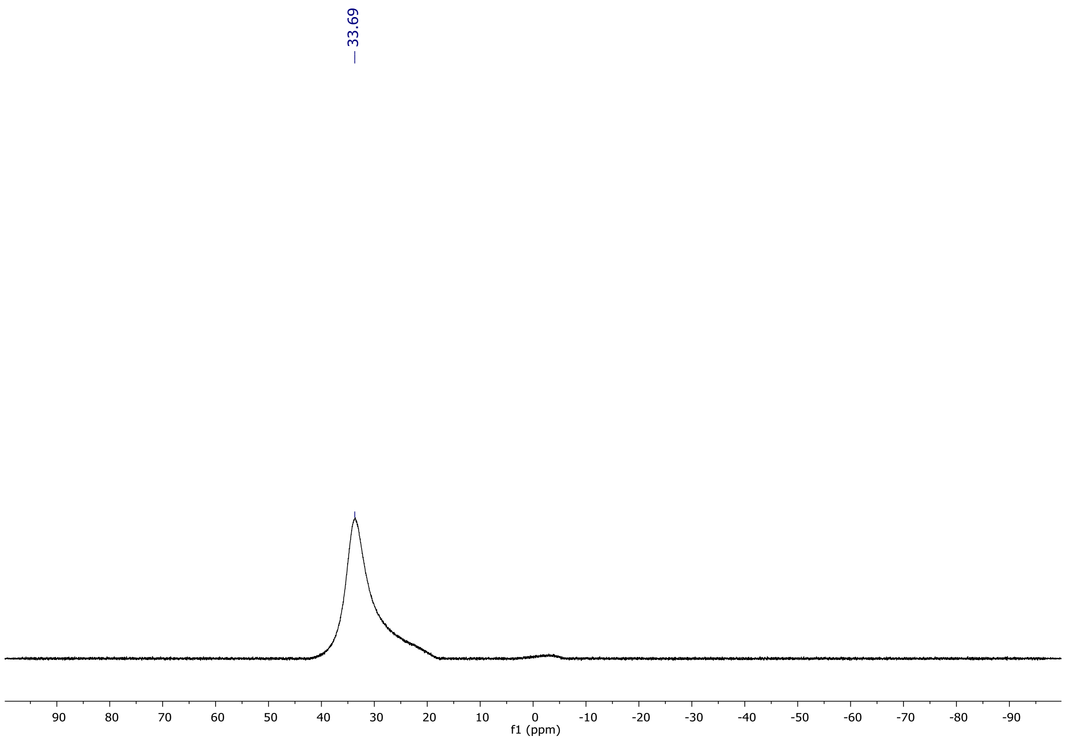

^{1}H-NMR (300 MHz, $CDCl_3$) of compound **2k**

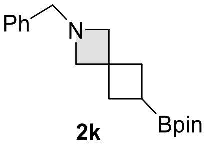

2k

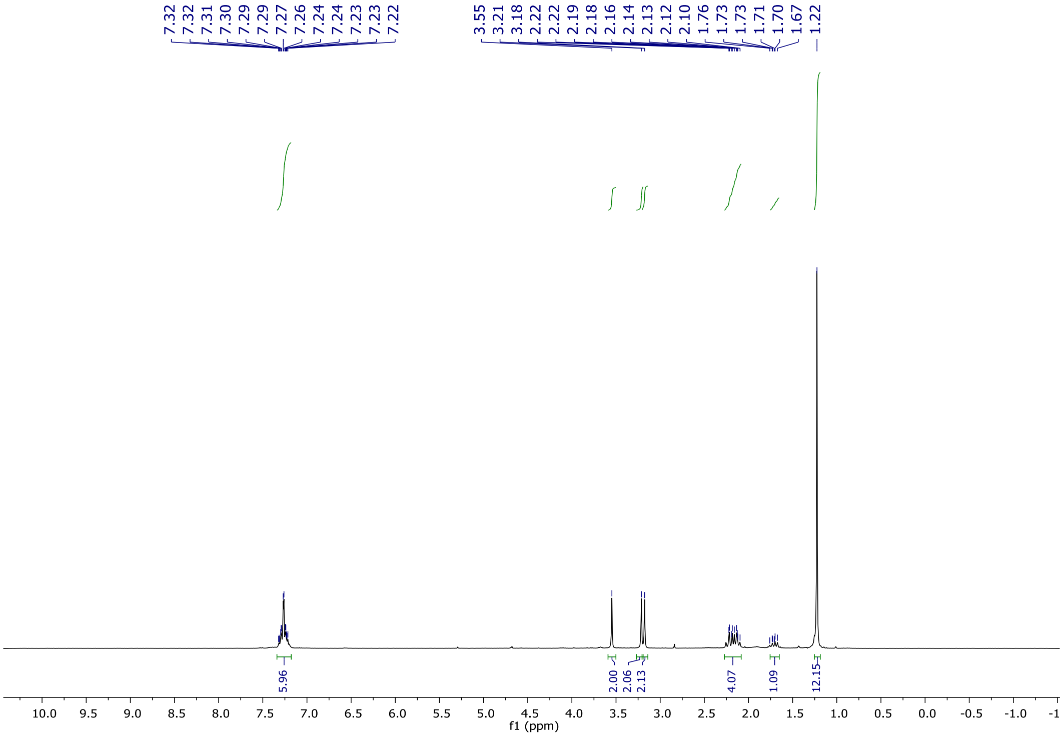

^{13}C-NMR (75 MHz, $CDCl_3$) of compound **2k**

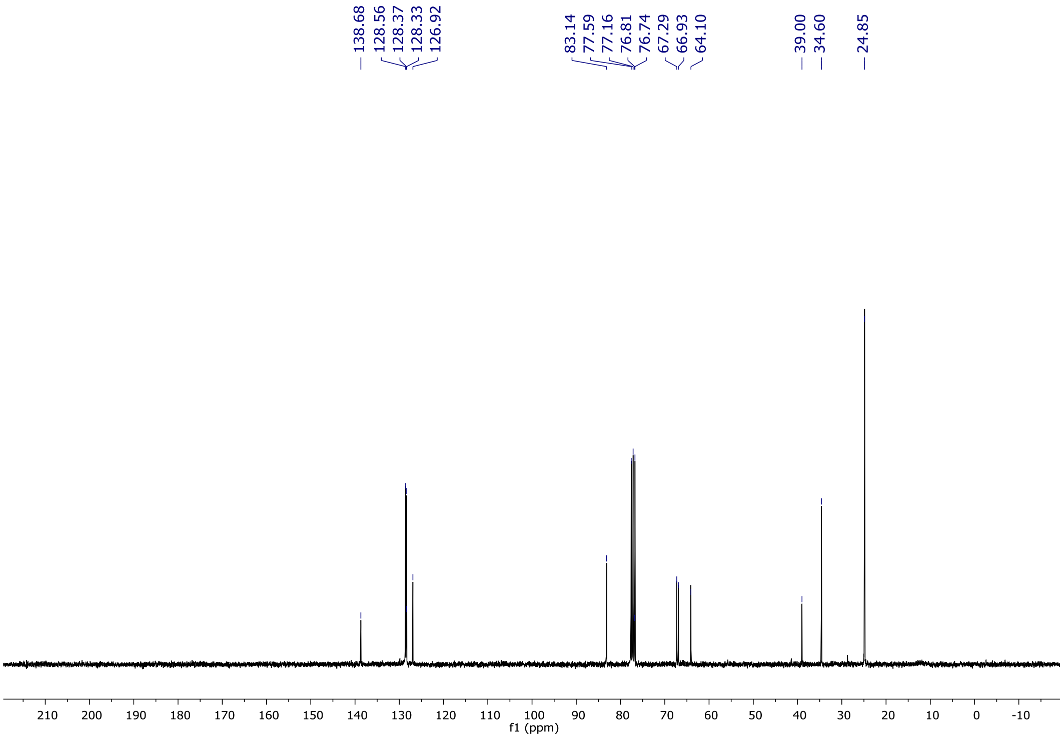

^{11}B-NMR (96 MHz, $CDCl_3$) of compound **2k**

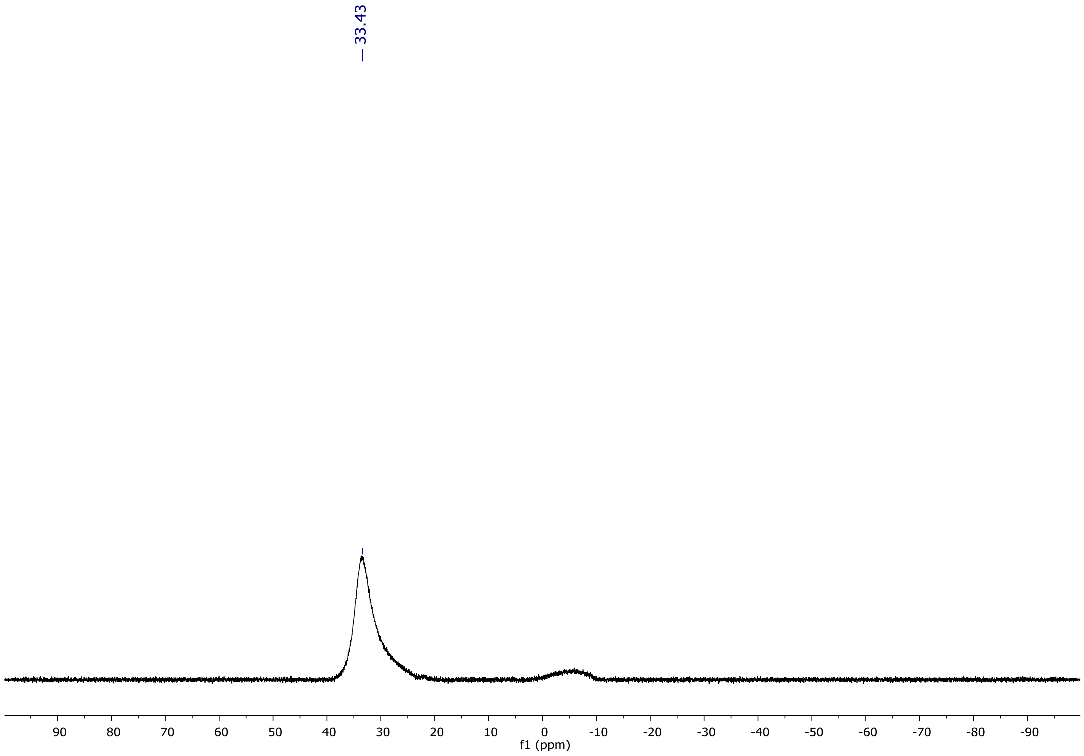

^{1}H-NMR (300 MHz, Toluene-d_8, 373K) of compound **2l**

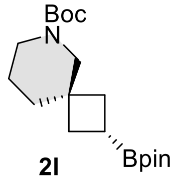

2l

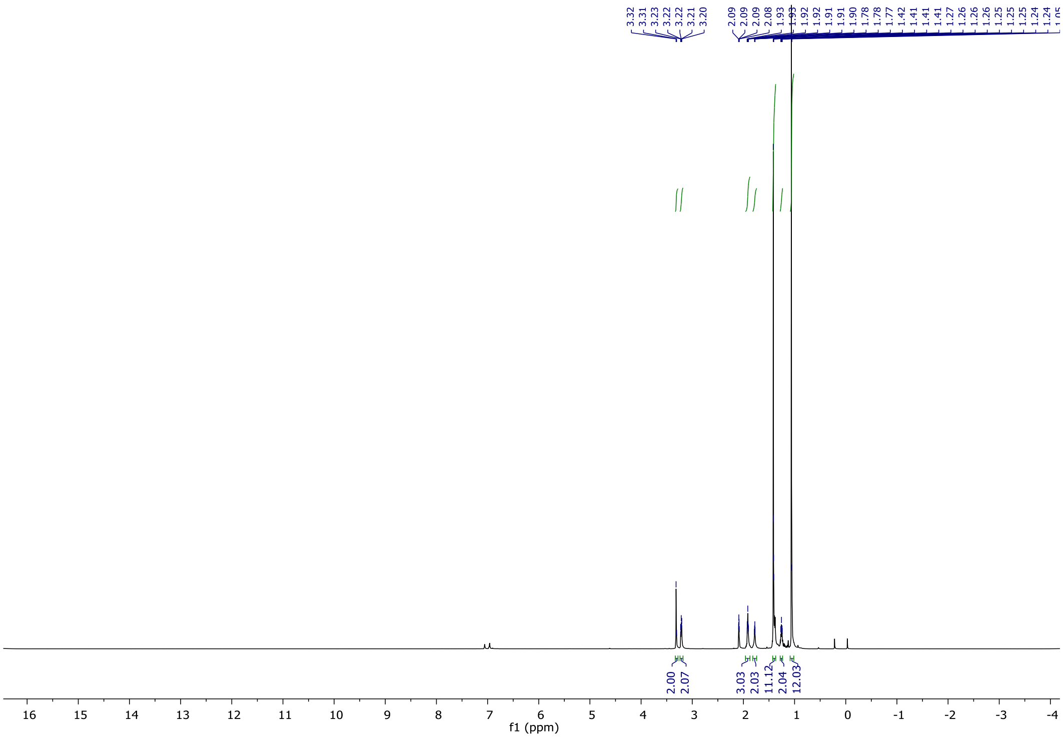

^{13}C-NMR (75 MHz, Toluene-d_8, 373K) of compound **2l**

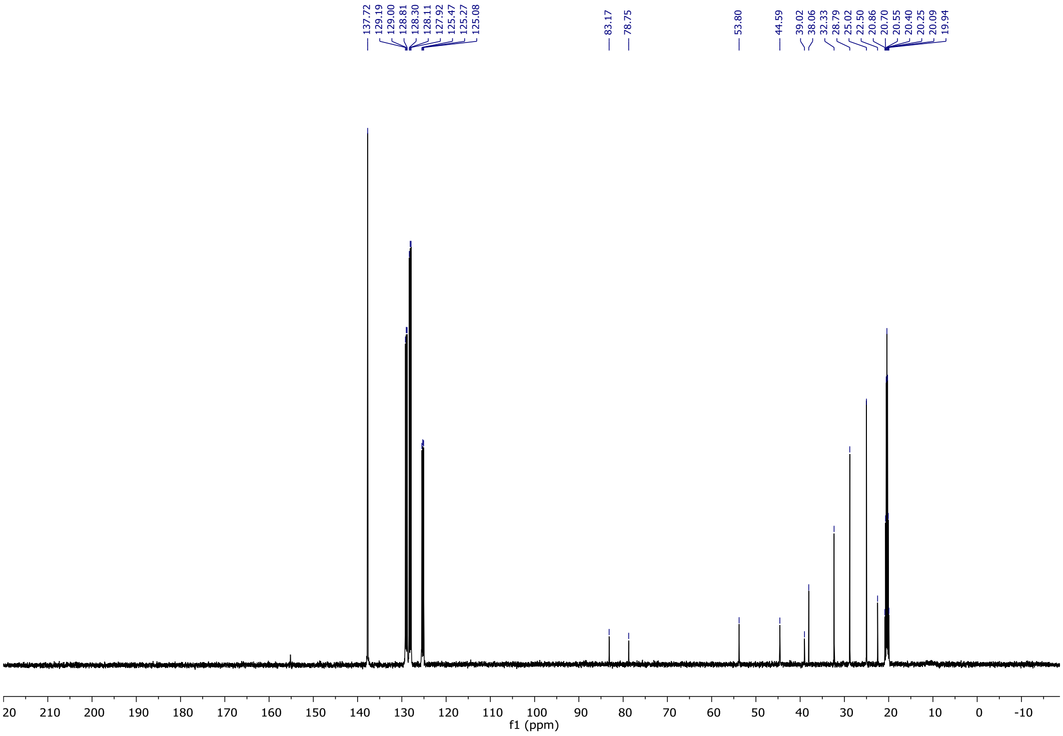

^{1}H-NMR (300 MHz, $CDCl_3$) of compound **2m**

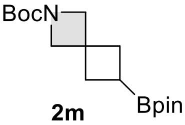

2m

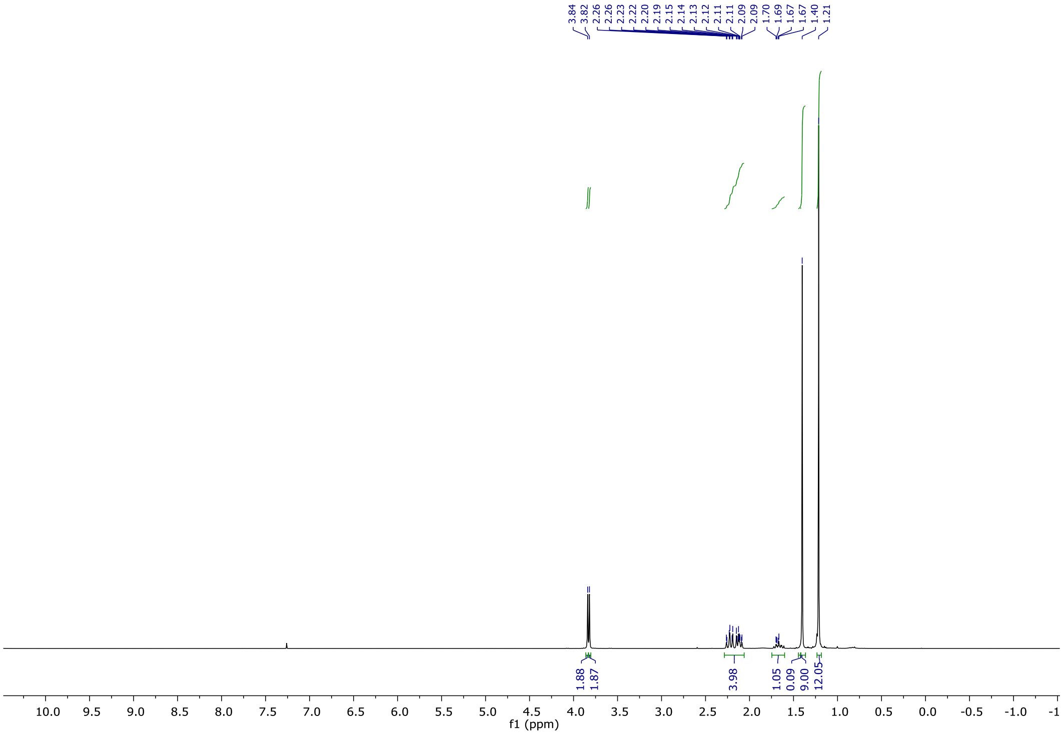

^{13}C-NMR (75 MHz, $CDCl_3$) of compound **2m**

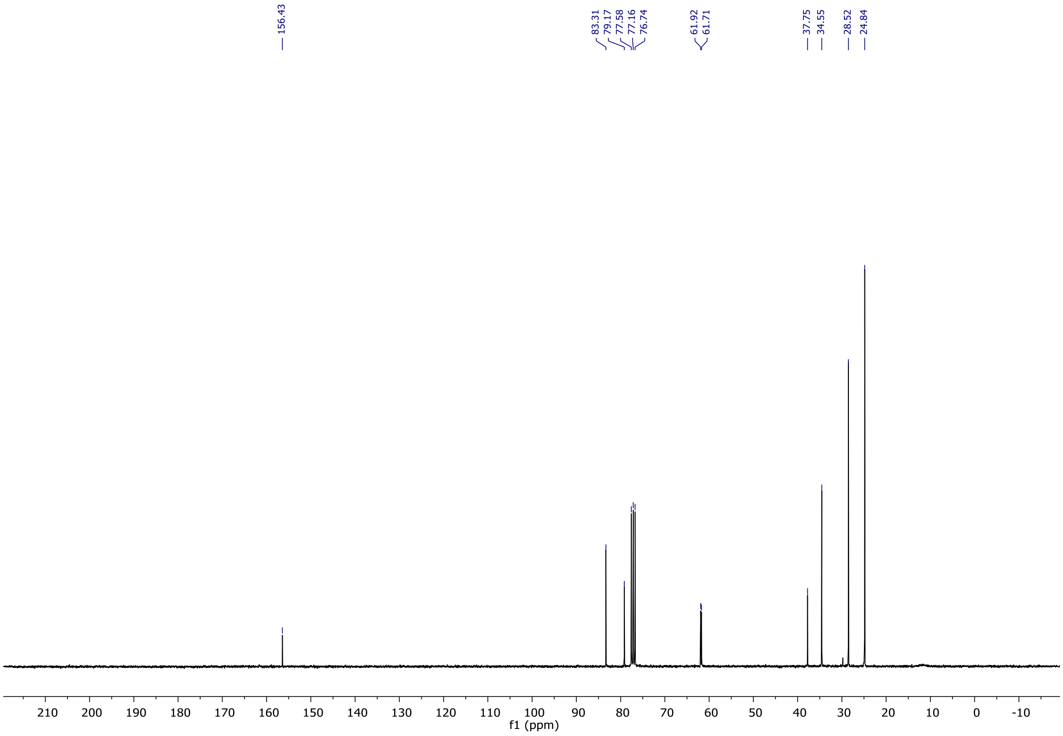

^{1}H-NMR (300 MHz, $CDCl_3$) of compound **SI-4**

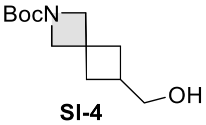

SI-4

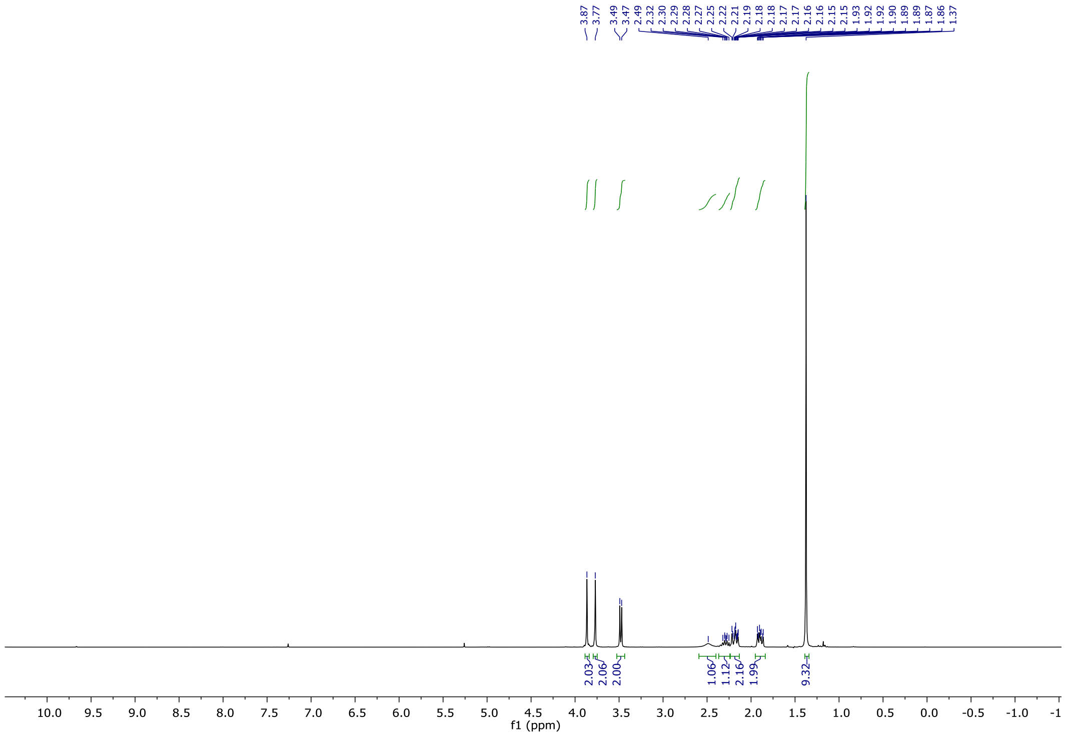

^{13}C-NMR (75 MHz, $CDCl_3$) of compound **SI-4**

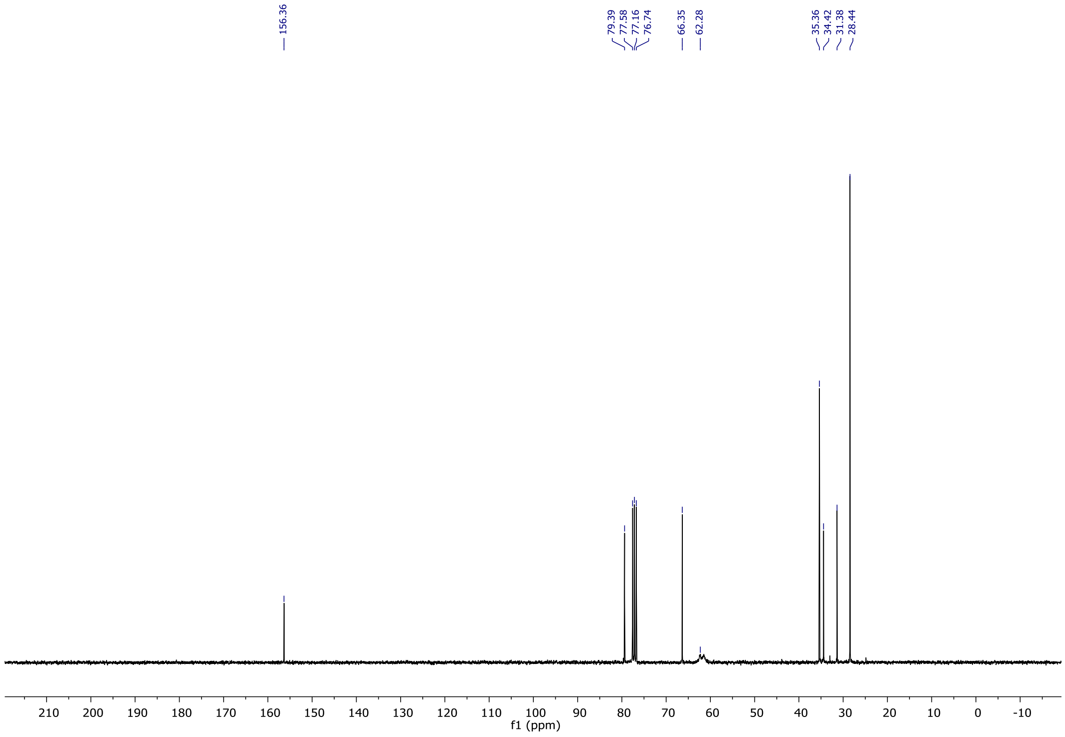

^{1}H-NMR (300 MHz, $CDCl_3$) of compound **4**

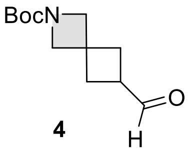

4

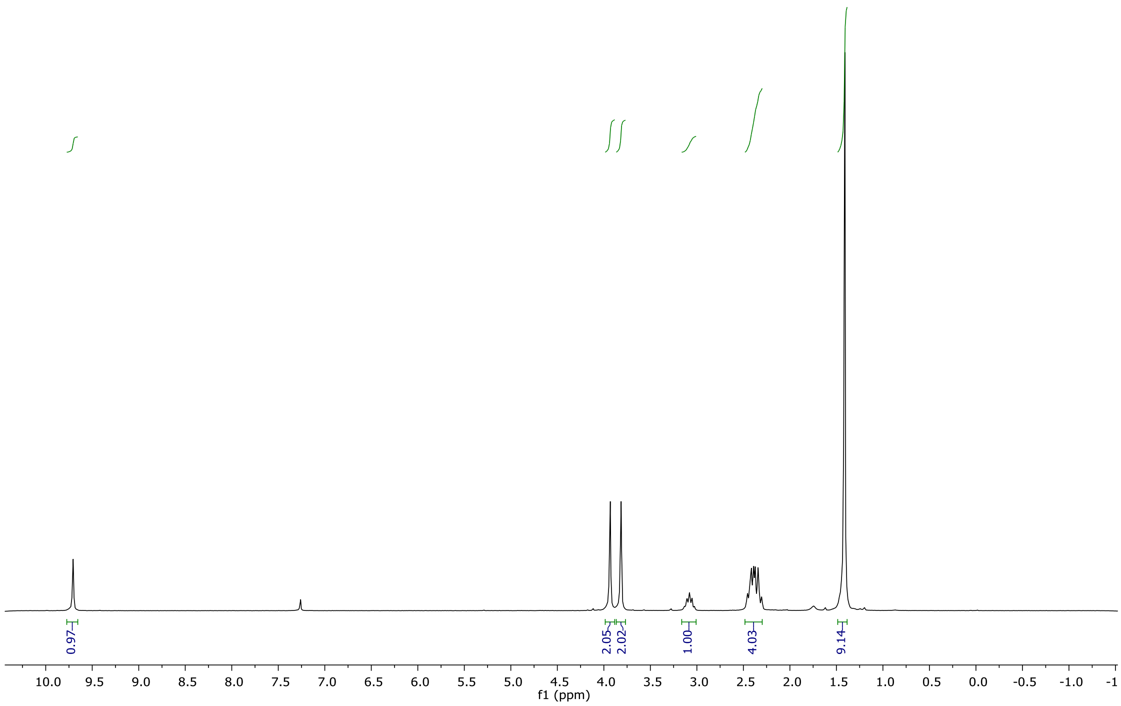

^{13}C-NMR (75 MHz, $CDCl_3$) of compound **4**

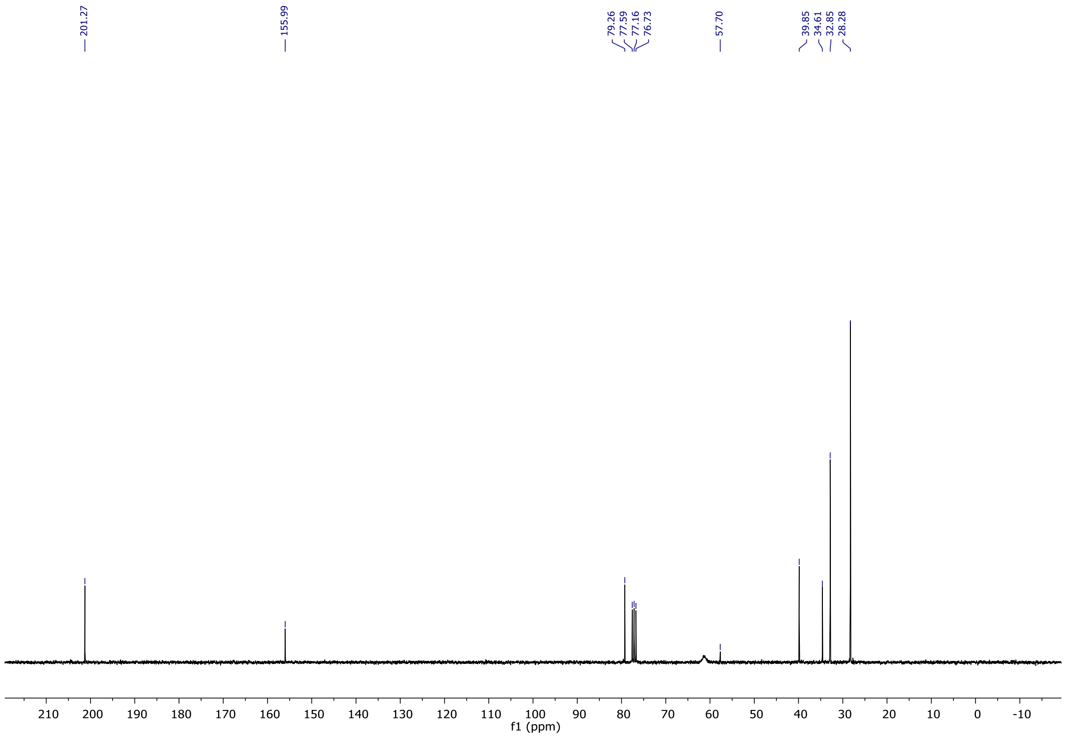

^{1}H-NMR (300 MHz, $CDCl_3$) of compound **5**

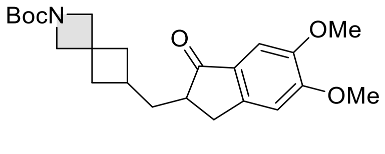

5

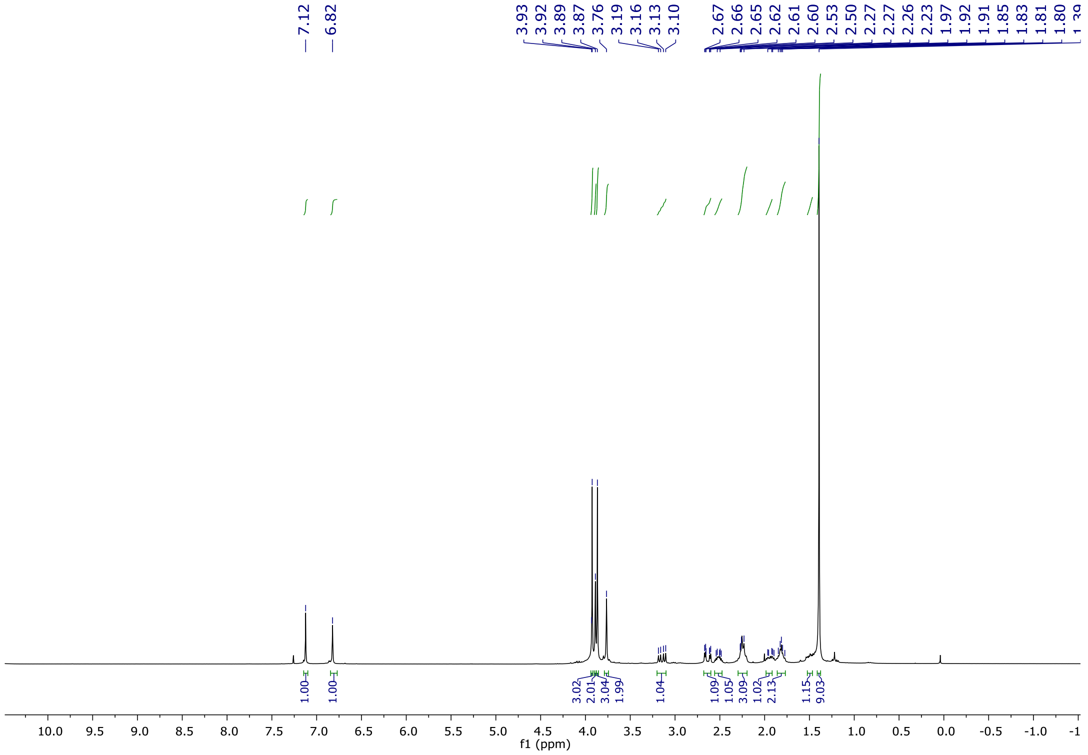

^{13}C-NMR (75 MHz, $CDCl_3$) of compound **5**

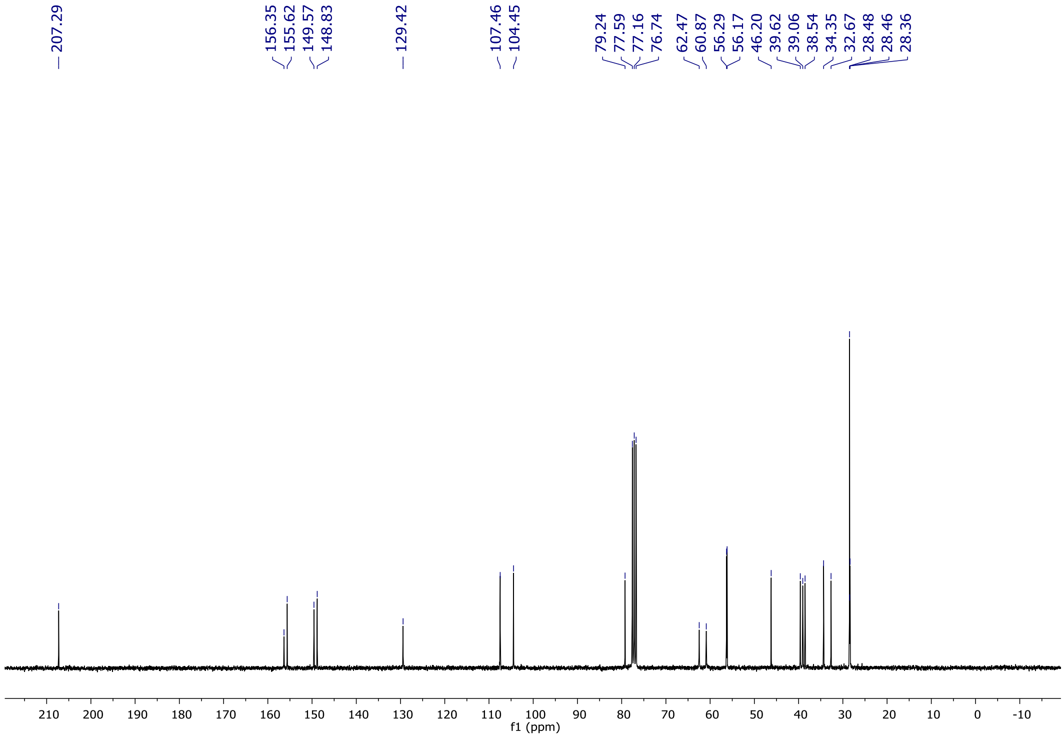

^{1}H-NMR (300 MHz, $CDCl_3$) of compound **6**

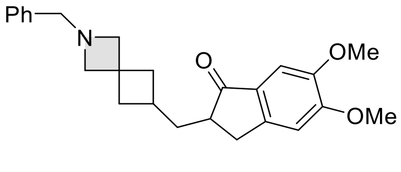

6

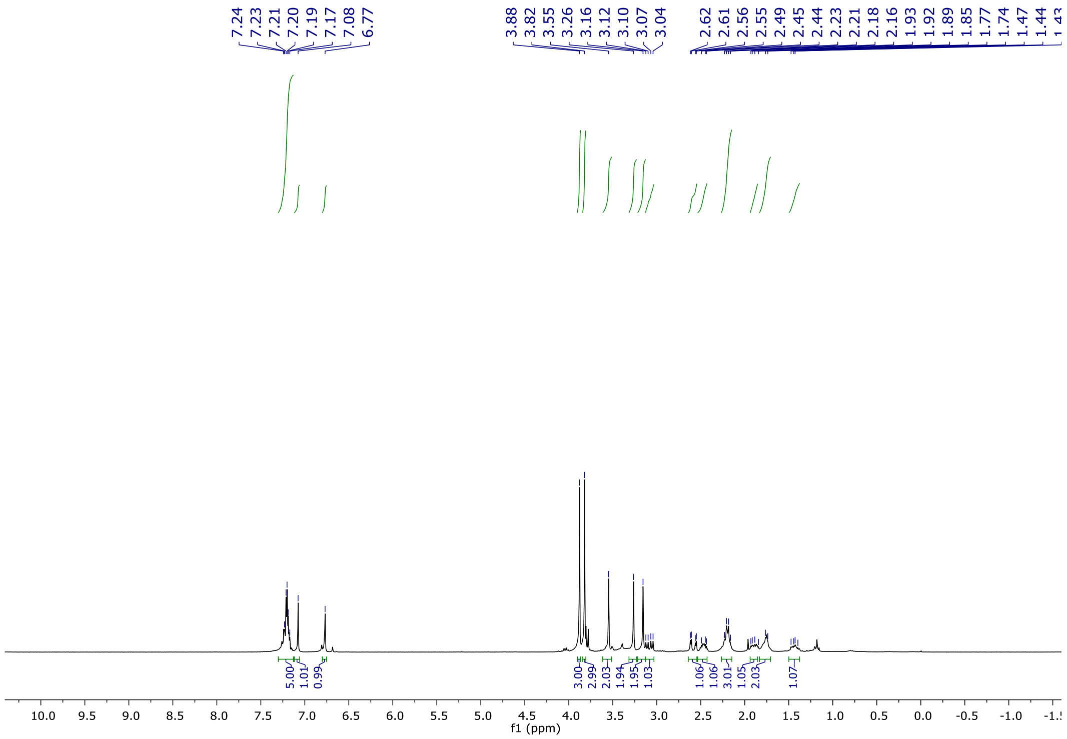

^{13}C-NMR (75 MHz, $CDCl_3$) of compound **6**

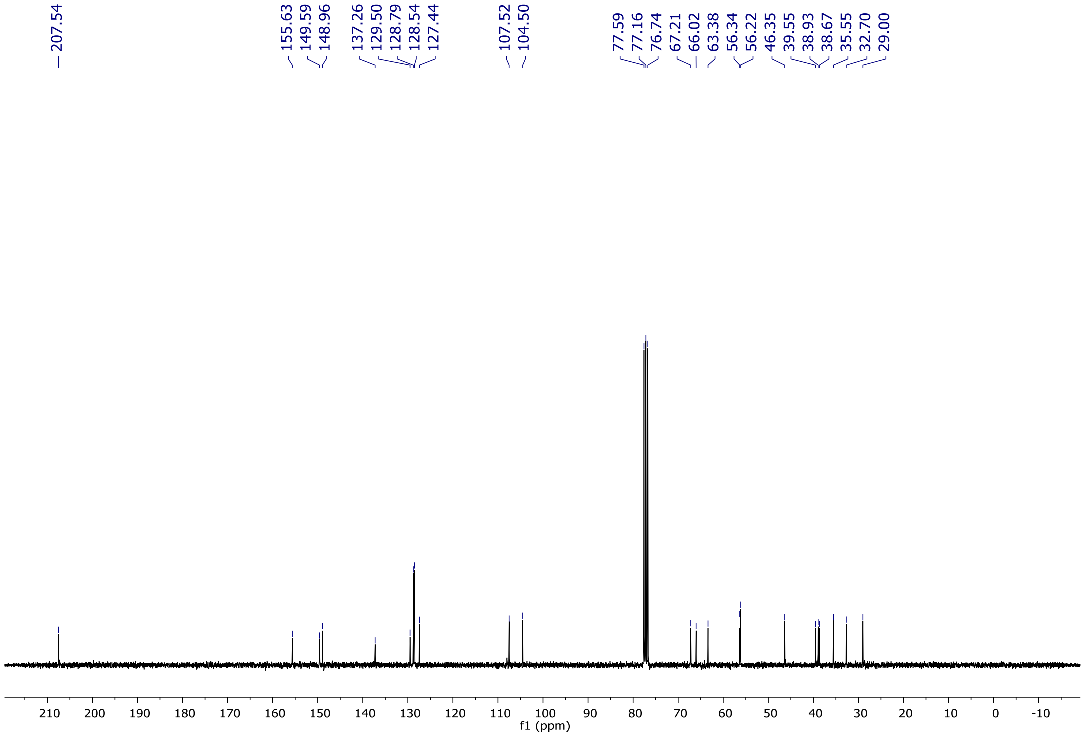

^{1}H-NMR (300 MHz, $CDCl_3$) of compound **7**

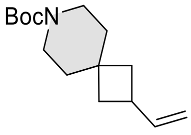

7

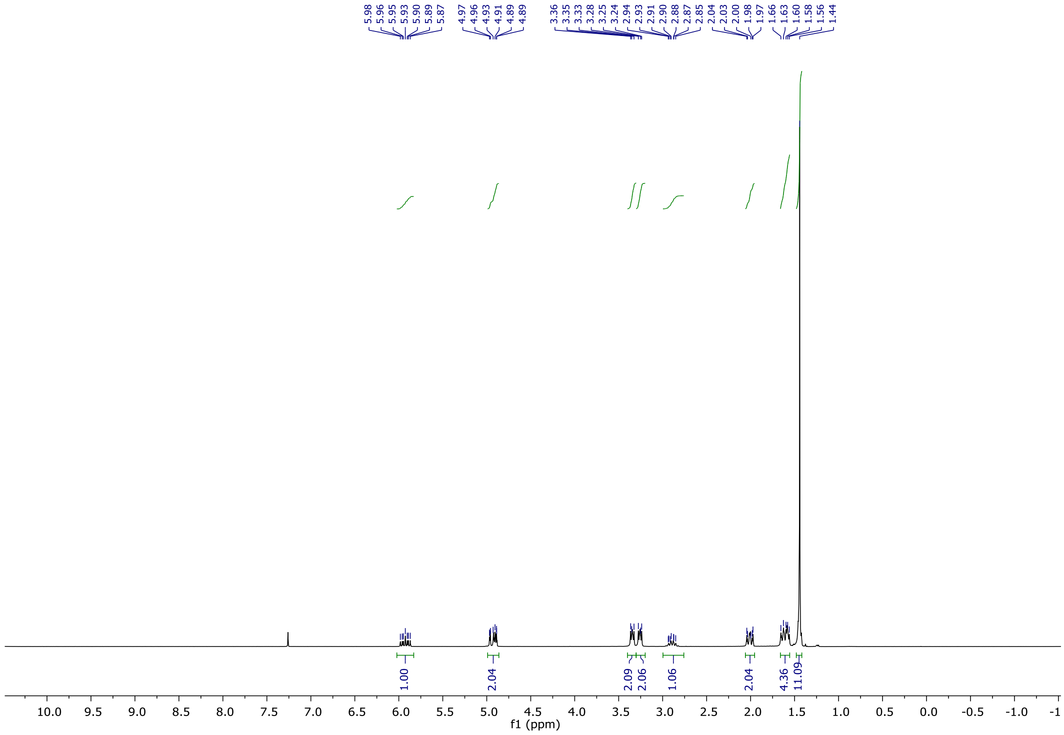

^{13}C-NMR (75 MHz, $CDCl_3$) of compound **7**

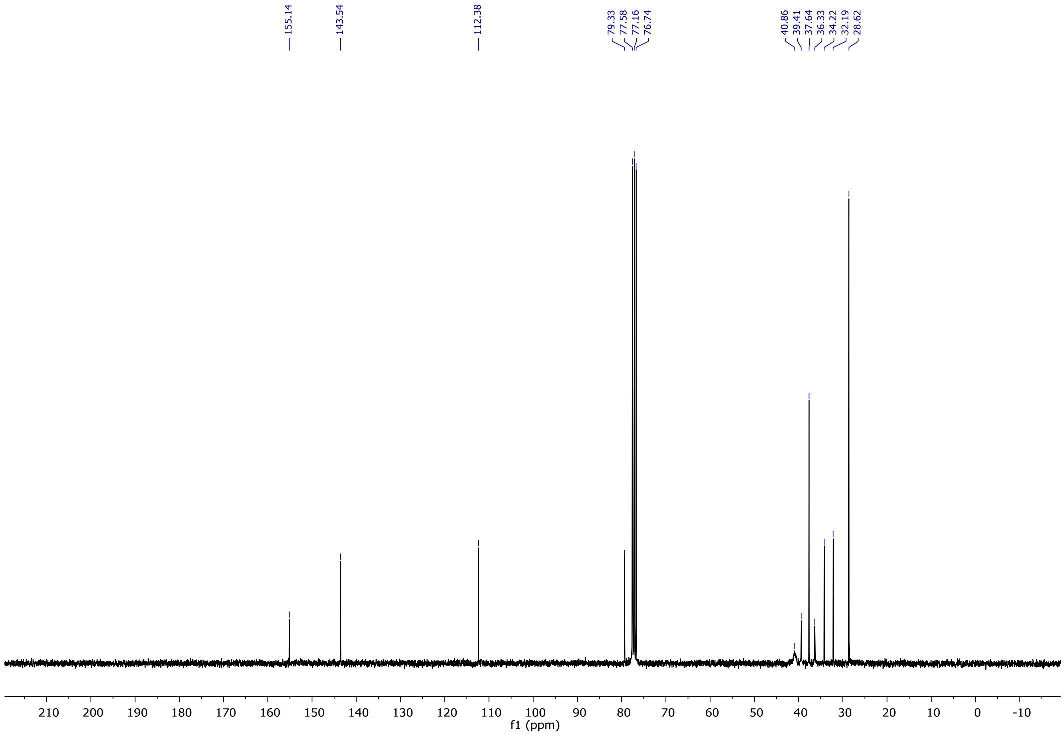

^{1}H-NMR (300 MHz, $CDCl_3$) of compound **8**

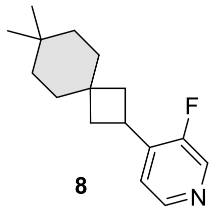

8

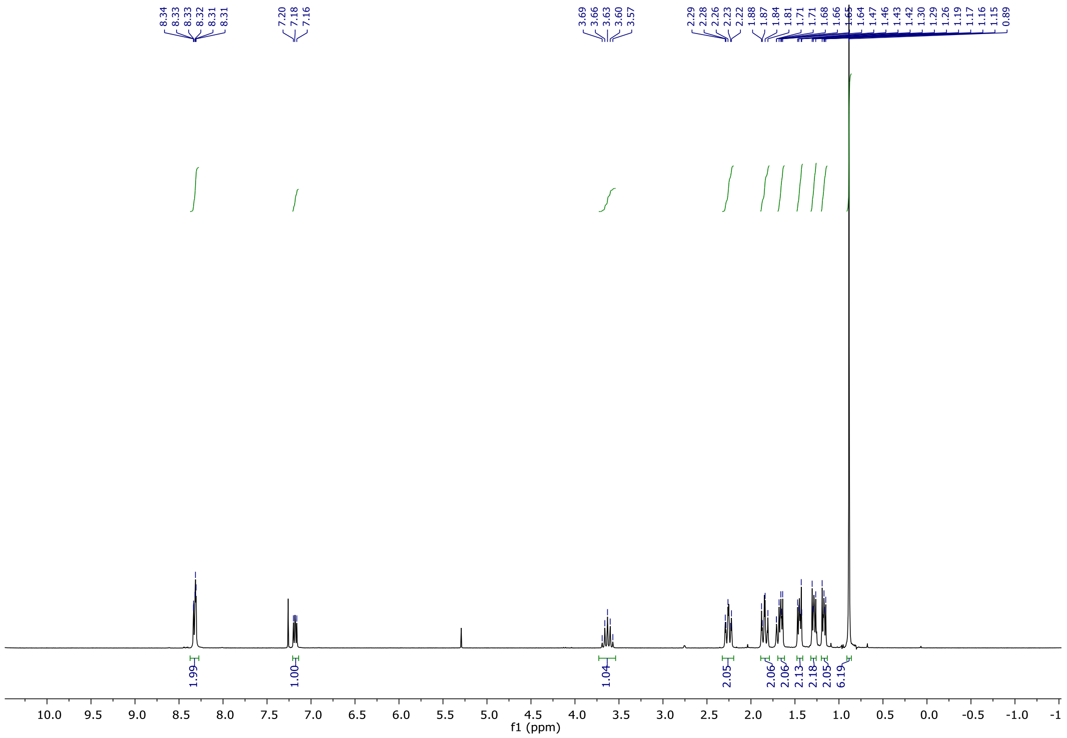

^{13}C-NMR (75 MHz, $CDCl_3$) of compound **8**

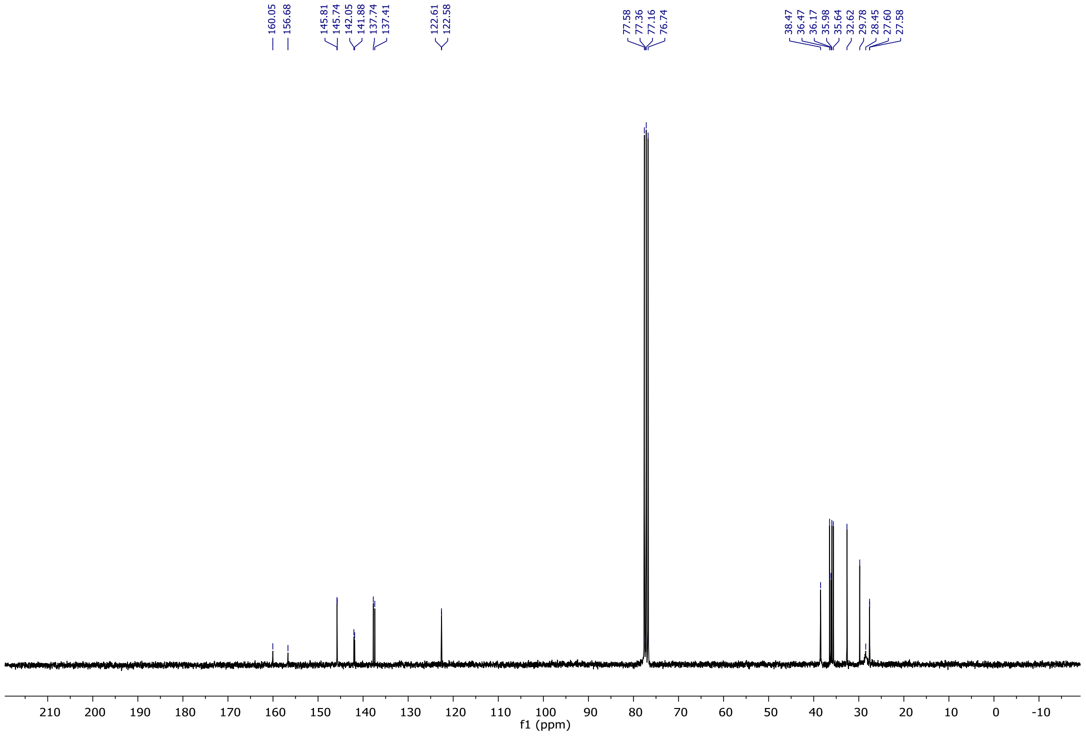

^{19}F-NMR (282 MHz, $CDCl_3$) of compound **8**

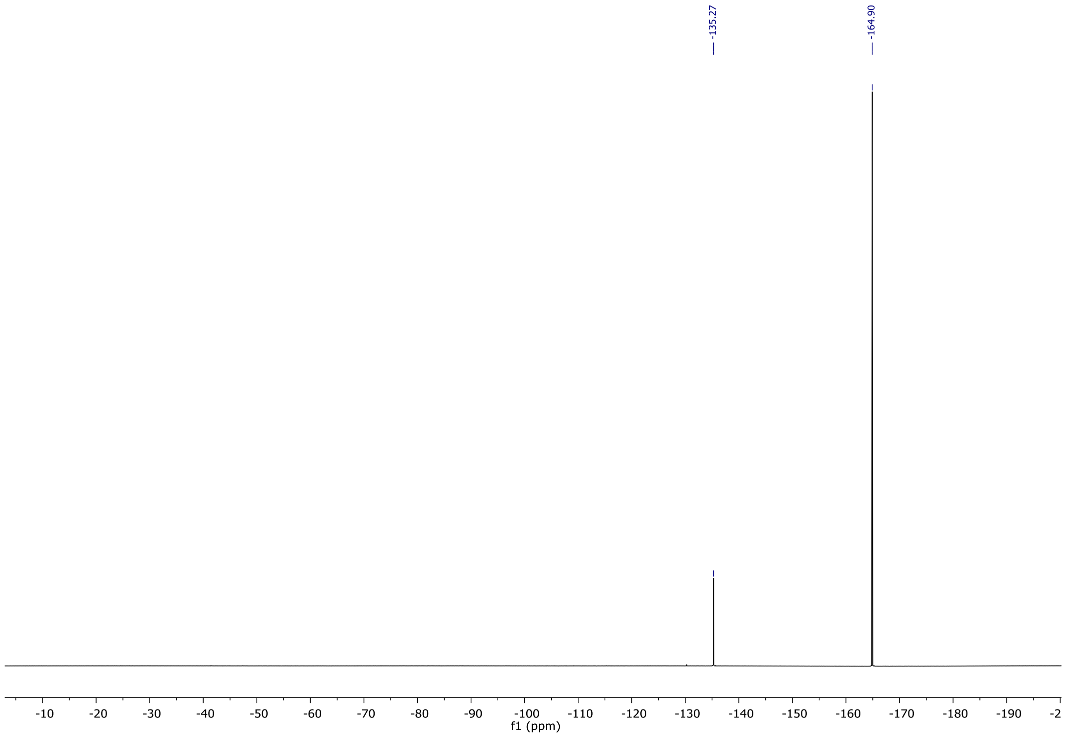

^{1}H-NMR (300 MHz, D_2O) of compound **9**

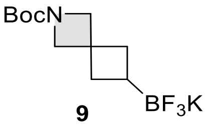

9

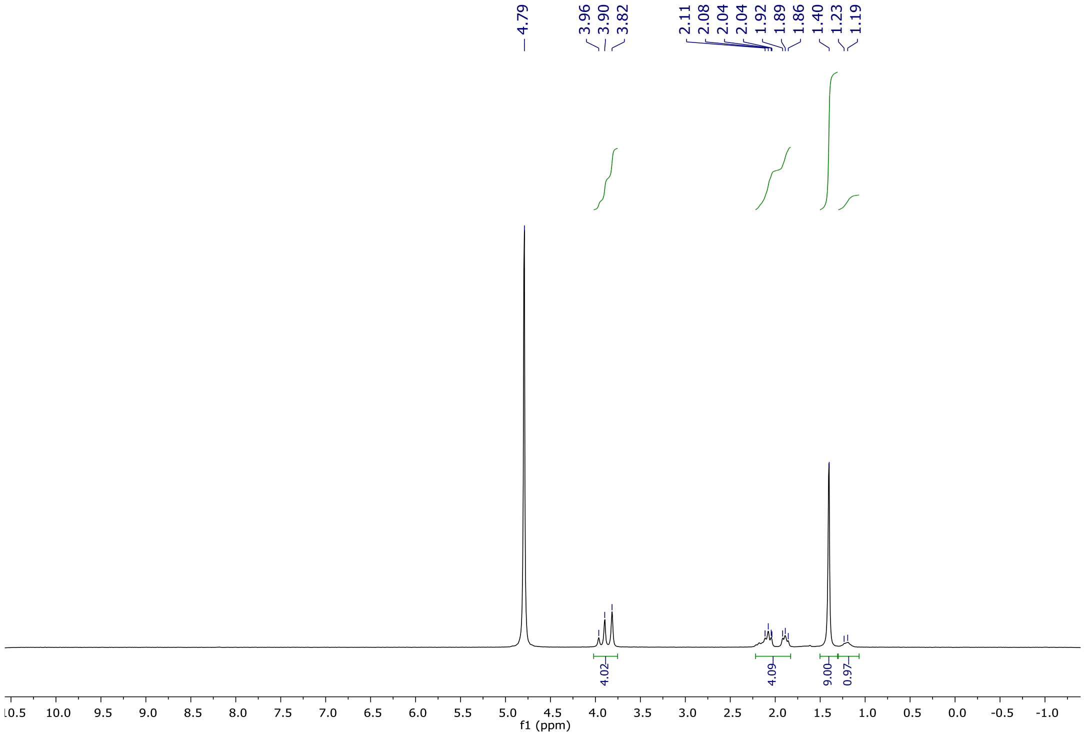

^{13}C-NMR (75 MHz, D_2O) of compound **9**

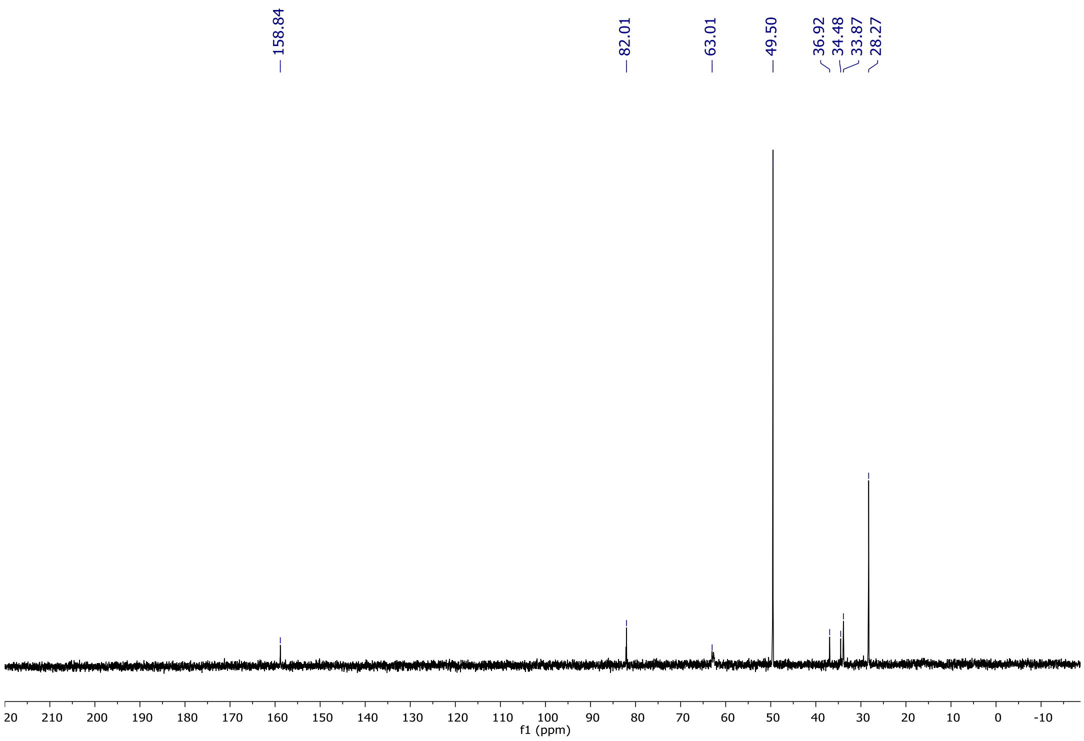

^{1}H-NMR (300 MHz, $CDCl_3$) of compound **10**

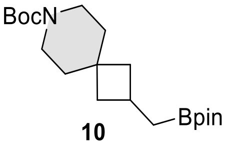

10

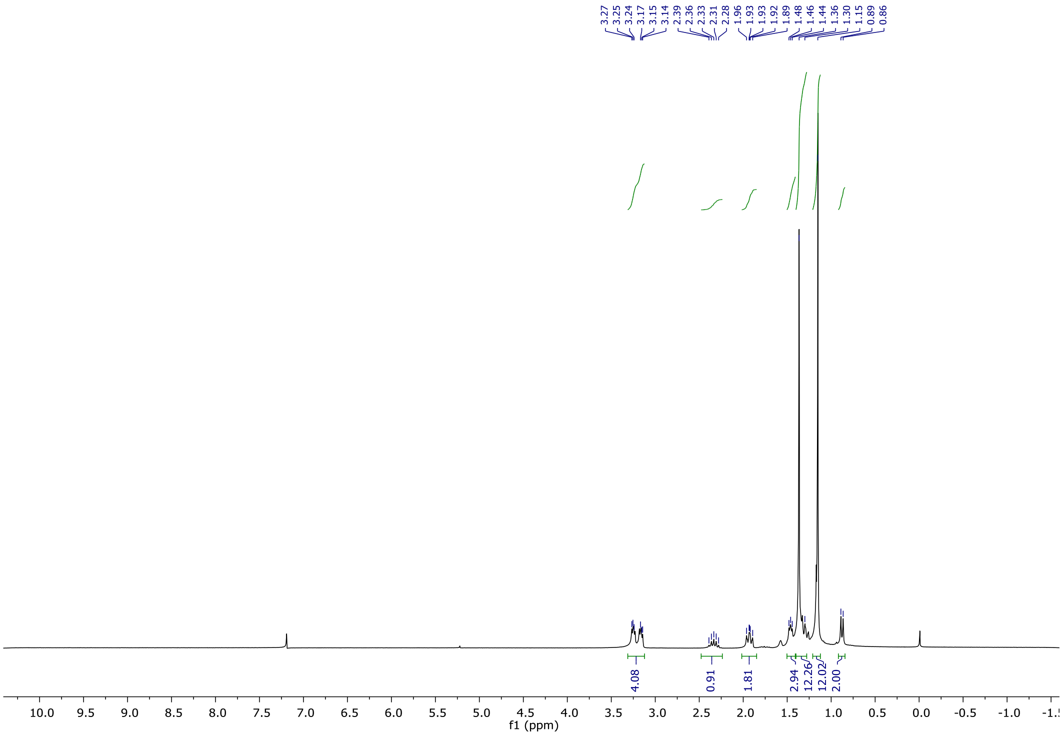

^{13}C-NMR (75 MHz, $CDCl_3$) of compound **10**

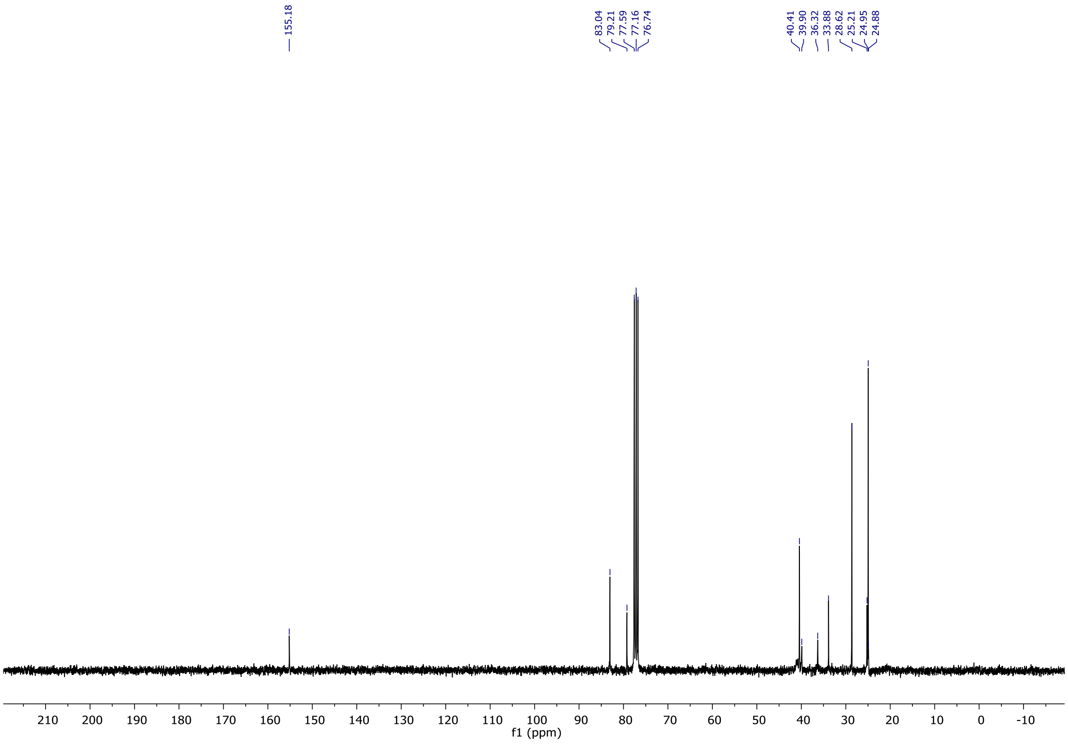

^{1}H-NMR (300 MHz, $CDCl_3$) of compound **11**

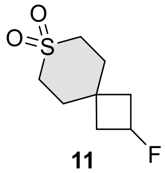

11

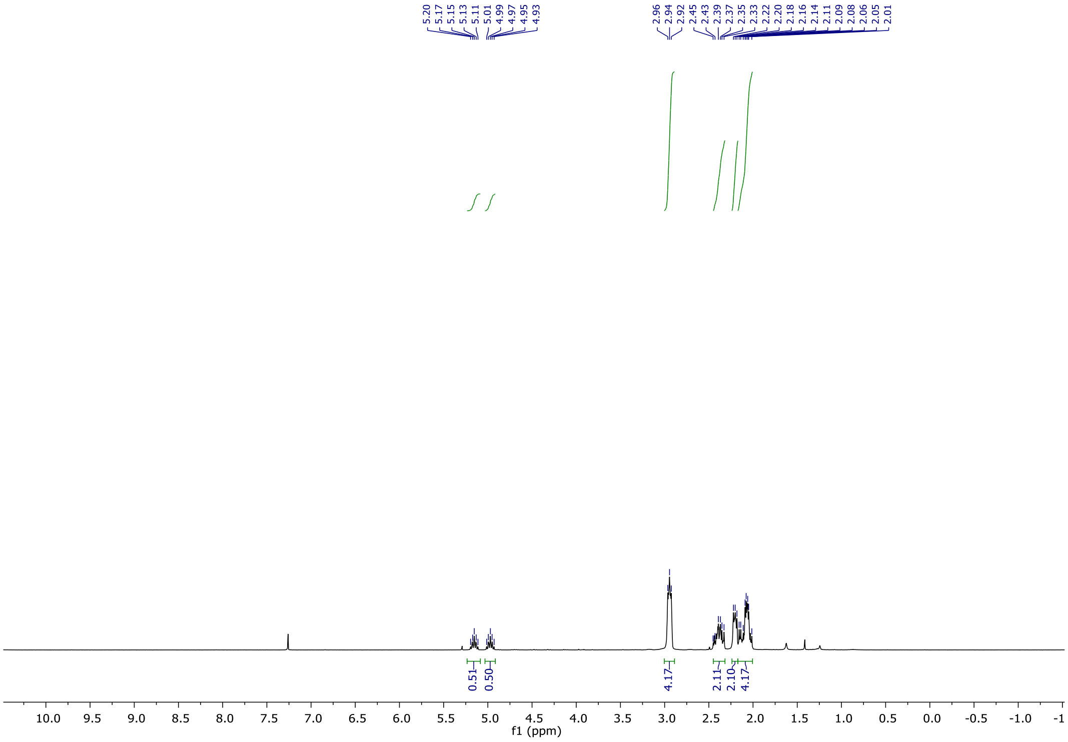

^{13}C-NMR (75 MHz, $CDCl_3$) of compound **11**

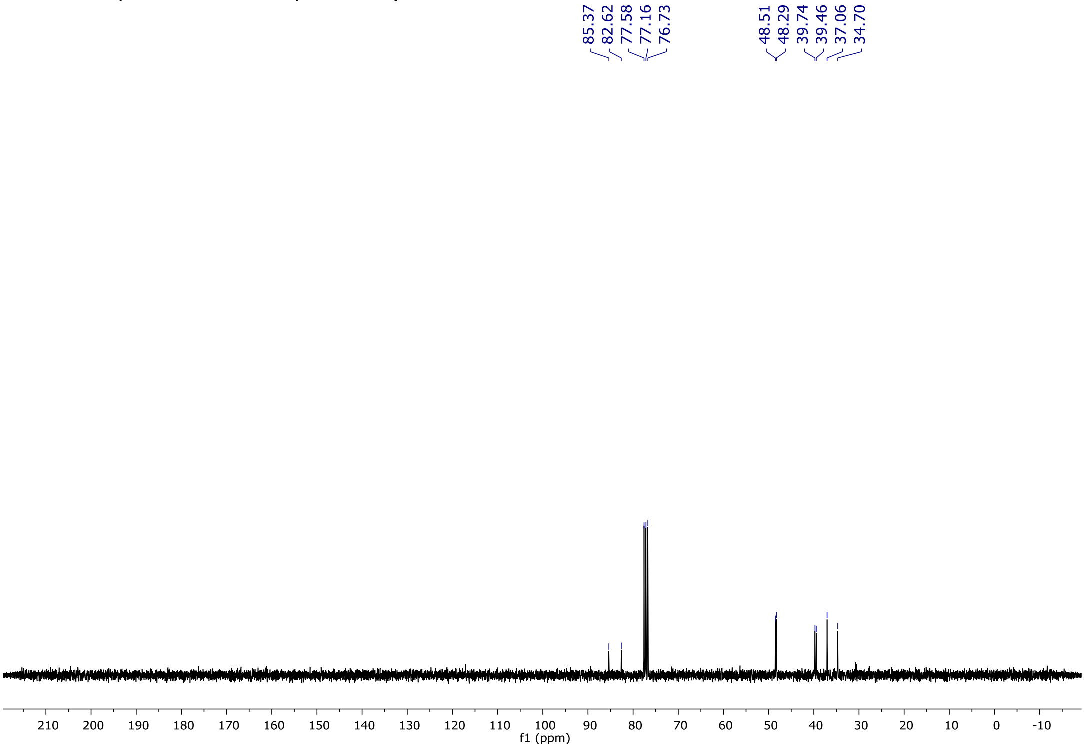

^{19}F-NMR (282 MHz, $CDCl_3$) of compound **11**

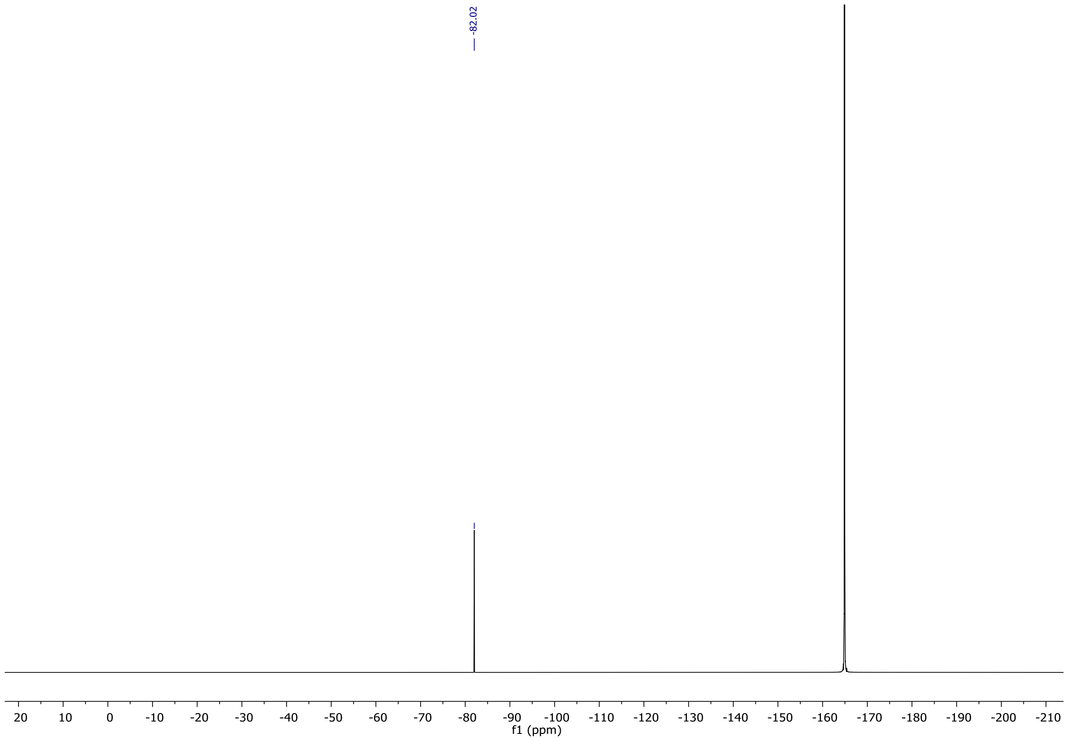

11. X-RAY Data

Crystal Structure Report for compound 2l[13]

Compound **2l** was recrystallized (hexane) in order to obtain appropriate crystals for X-ray analysis.

A single crystal of compound **2l** was covered with a layer of an inert mineral oil, mounted on a MiTeGen micromount with the aid of a microscope, and placed under a low temperature nitrogen stream. The intensity data set was collected at 250 K on a Bruker Kappa Apex II diffractometer equipped with a Mo sealed tube and graphite monocromator. The dataset was integrated with SAINT, the structure was solved with SHELXS-97 and the model refined by a least-squares method against F2 with SHELXL-2014. Data were corrected for absorption effects using the multi-scan method SADABS. All the hydrogen atoms were positioned geometrically and refined using a riding model, while the non-hydrogen atoms were refined anisotropically.[14]

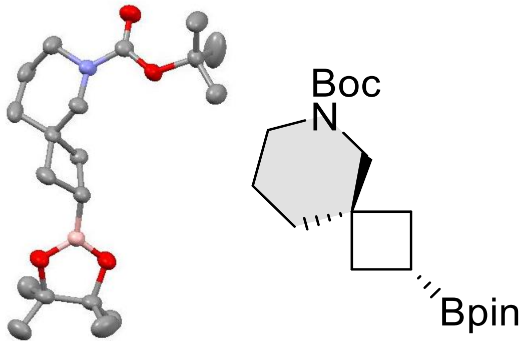

Figure S2: X-ray crystallography of compound **2l**.

13 CCDC 2041652 contains the supplementary crystallographic data. These data can be obtained free of charge at www.ccdc.cam.ac.uk/conts/retrieving.html

[14] González, C.; Schlegel, H. B. *J. Phys. Chem*. **1990**, *94*, 5523.

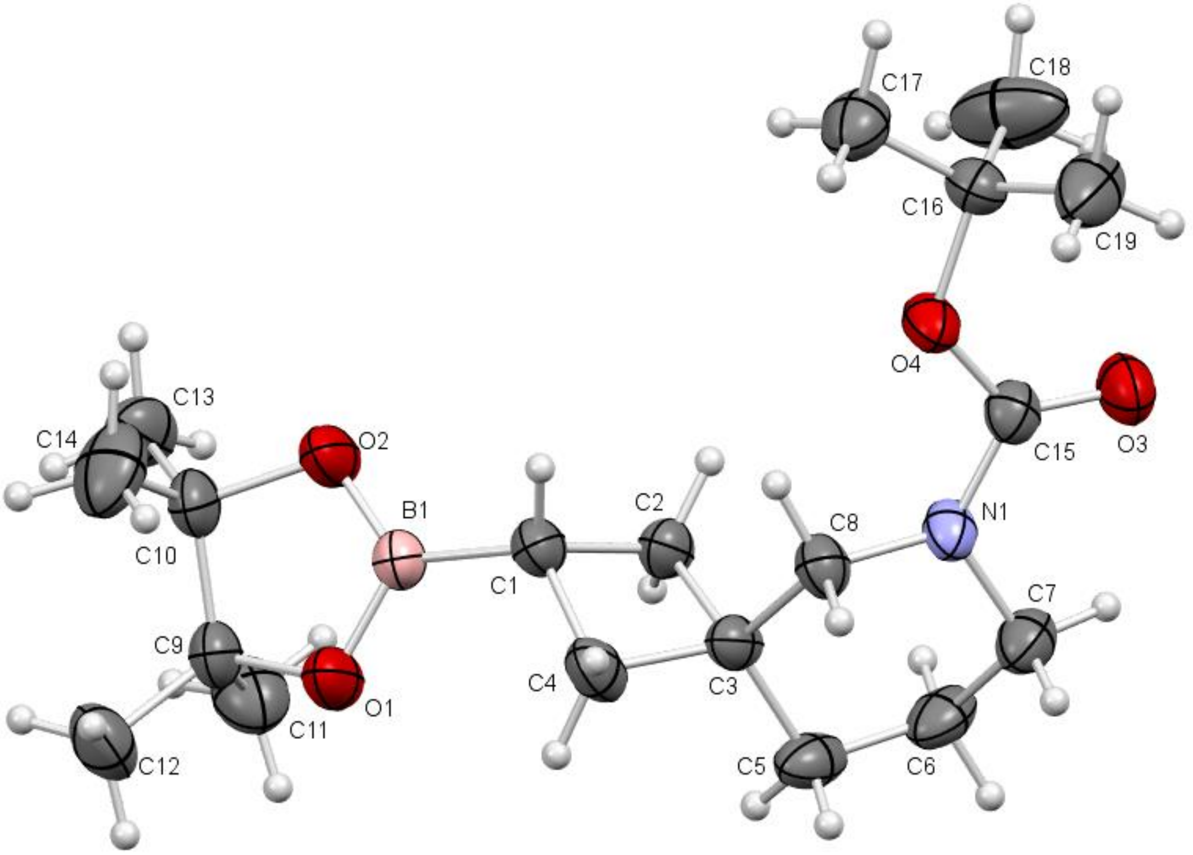

Figure S3. Ellipsoid plot (50% probability level) of compound **2l** with non-hydrogen atoms labeled.

Data collection details for 2l

A clear colourless prismatic-like specimen of $C_{19}H_{34}BNO_4$, approximate dimensions 0.049 mm x 0.056 mm x 0.229 mm, was used for the X-ray crystallographic analysis. The X-ray intensity data were measured. The integration of the data using a triclinic unit cell yielded a total of 30136 reflections to a maximum θ angle of 25.34° (0.83 Å resolution), of which 3765 were independent (average redundancy 8.004, completeness = 99.4%, R_{int} = 5.28%, R_{sig} = 3.33%) and 2546 (67.62%) were greater than 2σ(F^2). The final cell constants of a = 6.1650(4) Å, b = 11.7928(7) Å, c = 14.2810(8) Å, α = 86.510(3)°, β = 88.691(3)°, γ = 86.448(3)°, volume = 1034.16(11) Å^3, are based upon the refinement of the XYZ-centroids of reflections above 20 σ(I). The calculated minimum and maximum transmission coefficients (based on crystal size) are 0.9830 and 0.9960. The final anisotropic full-matrix least-squares refinement on F^2 with 233 variables converged at R1 = 5.65%, for the observed data and wR2 = 18.74% for all data. The goodness-of-fit was 1.007. The largest peak in the final difference electron density synthesis was 0.427 e^-/Å^3 and the largest hole was -0.262 e^-/Å^3 with an RMS deviation of 0.059 e^-/Å^3. On the basis of the final model, the calculated density was 1.128 g/cm^3 and F(000), 384 e^-.

Table 1. Sample and crystal data for 2l

**Chemical formula**	$C_{19}H_{34}BNO_4$	
**Formula weight**	351.28 g/mol	
**Temperature**	250(2) K	
**Wavelength**	0.71073 Å	
**Crystal size**	0.049 x 0.056 x 0.229 mm	
**Crystal habit**	clear colourless prismatic	
**Crystal system**	triclinic	
**Space group**	P -1	
**Unit cell dimensions**	a = 6.1650(4) Å	α = 86.510(3)°
	b = 11.7928(7) Å	β = 88.691(3)°
	c = 14.2810(8) Å	γ = 86.448(3)°
**Volume**	1034.16(11) $Å^3$	
**Z**	2	
**Density (calculated)**	1.128 g/cm^3	
**Absorption coefficient**	0.077 mm^{-1}	
**F(000)**	384	

Table 2. Data collection and structure refinement for 2l

**Theta range for data collection**	2.18 to 25.34°	
**Index ranges**	-7<=h<=7, -14<=k<=14, -17<=l<=17	
**Reflections collected**	30136	
**Independent reflections**	3765 [R(int) = 0.0528]	
**Max. and min. transmission**	0.9960 and 0.9830	
**Refinement method**	Full-matrix least-squares on F^2	
**Refinement program**	SHELXL-2014/7 (Sheldrick, 2014)	
**Function minimized**	Σ w(F_o^2 - F_c^2)^2	
**Data / restraints / parameters**	3765 / 0 / 233	
**Goodness-of-fit on F^2**	1.007	
**Final R indices**	2546 data; I>2σ(I)	R1 = 0.0565, wR2 = 0.1609
	all data	R1 = 0.0885, wR2 = 0.1874
**Weighting scheme**	$w=1/[\sigma^2(F_o^2)+(0.1104P)^2+0.3231P]$ where $P=(F_o^2+2F_c^2)/3$	
**Largest diff. peak and hole**	0.427 and -0.262 $eÅ^{-3}$	
**R.M.S. deviation from mean**	0.059 $eÅ^{-3}$	

Table 3. Atomic coordinates and equivalent isotropic atomic displacement parameters ($Å^2$) for 2l

U(eq) is defined as one third of the trace of the orthogonalized U_{ij} tensor.

	x/a	y/b	z/c	U(eq)
B1	0.9015(4)	0.7080(2)	0.77082(19)	0.0385(6)
C1	0.8496(4)	0.58241(19)	0.75732(16)	0.0404(6)
C2	0.6352(4)	0.53774(18)	0.80159(17)	0.0400(6)
C3	0.7626(4)	0.42610(17)	0.83480(15)	0.0366(5)
C4	0.9748(4)	0.48630(19)	0.81562(18)	0.0460(6)
C5	0.7169(5)	0.3800(2)	0.93413(16)	0.0513(7)
C6	0.4977(5)	0.3284(2)	0.94266(16)	0.0504(7)
C7	0.4823(5)	0.2366(2)	0.87418(16)	0.0472(6)
C8	0.7422(4)	0.33280(19)	0.76691(16)	0.0387(5)
C9	0.0208(4)	0.86250(19)	0.83827(17)	0.0435(6)
C10	0.9476(4)	0.89577(19)	0.73740(17)	0.0466(6)
C11	0.8560(5)	0.8992(3)	0.9124(2)	0.0629(8)
C12	0.2430(4)	0.8992(2)	0.8623(2)	0.0632(8)
C13	0.8015(5)	0.0030(2)	0.7263(2)	0.0643(8)
C14	0.1405(6)	0.9002(3)	0.6680(2)	0.0767(10)
C15	0.4127(4)	0.25080(18)	0.70805(16)	0.0370(5)
C16	0.3817(4)	0.2735(2)	0.53679(16)	0.0416(6)
C17	0.5197(5)	0.3347(2)	0.46367(18)	0.0566(7)
C18	0.1524(5)	0.3237(4)	0.5362(2)	0.0941(13)
C19	0.4006(6)	0.1475(2)	0.5204(2)	0.0728(10)
N1	0.5328(3)	0.28153(15)	0.77916(12)	0.0384(5)
O1	0.0321(3)	0.73863(14)	0.84004(12)	0.0544(5)
O2	0.8265(3)	0.79857(14)	0.71567(13)	0.0563(5)
O3	0.2569(3)	0.19368(15)	0.71808(12)	0.0528(5)
O4	0.4876(3)	0.29398(14)	0.62473(10)	0.0441(4)

Table 4. Bond lengths (Å) for 2l

B1-O2	1.352(3)	B1-O1	1.368(3)
B1-C1	1.559(3)	C1-C4	1.543(3)
C1-C2	1.560(3)	C1-H1	0.99
C2-C3	1.545(3)	C2-H2A	0.98
C2-H2B	0.98	C3-C5	1.515(3)
C3-C8	1.523(3)	C3-C4	1.538(3)
C4-H4A	0.98	C4-H4B	0.98
C5-C6	1.516(4)	C5-H5A	0.98
C5-H5B	0.98	C6-C7	1.511(3)
C6-H6A	0.98	C6-H6B	0.98
C7-N1	1.460(3)	C7-H7A	0.98

C7-H7B	0.98	C8-N1	1.462(3)
C8-H8A	0.98	C8-H8B	0.98
C9-O1	1.457(3)	C9-C11	1.514(4)
C9-C12	1.515(3)	C9-C10	1.540(3)
C10-O2	1.459(3)	C10-C13	1.509(4)
C10-C14	1.532(4)	C11-H11A	0.97
C11-H11B	0.97	C11-H11C	0.97
C12-H12A	0.97	C12-H12B	0.97
C12-H12C	0.97	C13-H13A	0.97
C13-H13B	0.97	C13-H13C	0.97
C14-H14A	0.97	C14-H14B	0.97
C14-H14C	0.97	C15-O3	1.208(3)
C15-O4	1.349(3)	C15-N1	1.350(3)
C16-O4	1.468(3)	C16-C18	1.499(4)
C16-C17	1.510(4)	C16-C19	1.515(4)
C17-H17A	0.97	C17-H17B	0.97
C17-H17C	0.97	C18-H18A	0.97
C18-H18B	0.97	C18-H18C	0.97
C19-H19A	0.97	C19-H19B	0.97
C19-H19C	0.97		

Table 5. Bond angles (°) for 2l

O2-B1-O1	112.2(2)	O2-B1-C1	124.5(2)
O1-B1-C1	123.2(2)	C4-C1-B1	118.61(19)
C4-C1-C2	87.94(17)	B1-C1-C2	118.6(2)
C4-C1-H1	110.0	B1-C1-H1	110.0
C2-C1-H1	110.0	C3-C2-C1	89.25(17)
C3-C2-H2A	113.8	C1-C2-H2A	113.8
C3-C2-H2B	113.8	C1-C2-H2B	113.8
H2A-C2-H2B	111.0	C5-C3-C8	109.73(19)
C5-C3-C4	117.8(2)	C8-C3-C4	110.65(19)
C5-C3-C2	116.9(2)	C8-C3-C2	111.81(18)
C4-C3-C2	88.62(16)	C3-C4-C1	90.13(17)
C3-C4-H4A	113.6	C1-C4-H4A	113.6
C3-C4-H4B	113.6	C1-C4-H4B	113.6
H4A-C4-H4B	110.9	C3-C5-C6	111.4(2)
C3-C5-H5A	109.3	C6-C5-H5A	109.3
C3-C5-H5B	109.3	C6-C5-H5B	109.3
H5A-C5-H5B	108.0	C7-C6-C5	111.0(2)
C7-C6-H6A	109.4	C5-C6-H6A	109.4
C7-C6-H6B	109.4	C5-C6-H6B	109.4
H6A-C6-H6B	108.0	N1-C7-C6	109.98(19)
N1-C7-H7A	109.7	C6-C7-H7A	109.7

N1-C7-H7B	109.7	C6-C7-H7B	109.7
H7A-C7-H7B	108.2	N1-C8-C3	111.13(18)
N1-C8-H8A	109.4	C3-C8-H8A	109.4
N1-C8-H8B	109.4	C3-C8-H8B	109.4
H8A-C8-H8B	108.0	O1-C9-C11	107.8(2)
O1-C9-C12	107.9(2)	C11-C9-C12	109.6(2)
O1-C9-C10	102.16(18)	C11-C9-C10	113.3(2)
C12-C9-C10	115.4(2)	O2-C10-C13	109.3(2)
O2-C10-C14	106.9(2)	C13-C10-C14	110.8(2)
O2-C10-C9	102.05(18)	C13-C10-C9	115.2(2)
C14-C10-C9	112.0(2)	C9-C11-H11A	109.5
C9-C11-H11B	109.5	H11A-C11-H11B	109.5
C9-C11-H11C	109.5	H11A-C11-H11C	109.5
H11B-C11-H11C	109.5	C9-C12-H12A	109.5
C9-C12-H12B	109.5	H12A-C12-H12B	109.5
C9-C12-H12C	109.5	H12A-C12-H12C	109.5
H12B-C12-H12C	109.5	C10-C13-H13A	109.5
C10-C13-H13B	109.5	H13A-C13-H13B	109.5
C10-C13-H13C	109.5	H13A-C13-H13C	109.5
H13B-C13-H13C	109.5	C10-C14-H14A	109.5
C10-C14-H14B	109.5	H14A-C14-H14B	109.5
C10-C14-H14C	109.5	H14A-C14-H14C	109.5
H14B-C14-H14C	109.5	O3-C15-O4	124.6(2)
O3-C15-N1	124.4(2)	O4-C15-N1	111.00(19)
O4-C16-C18	110.9(2)	O4-C16-C17	102.78(19)
C18-C16-C17	110.7(2)	O4-C16-C19	109.8(2)
C18-C16-C19	113.6(3)	C17-C16-C19	108.5(2)
C16-C17-H17A	109.5	C16-C17-H17B	109.5
H17A-C17-H17B	109.5	C16-C17-H17C	109.5
H17A-C17-H17C	109.5	H17B-C17-H17C	109.5
C16-C18-H18A	109.5	C16-C18-H18B	109.5
H18A-C18-H18B	109.5	C16-C18-H18C	109.5
H18A-C18-H18C	109.5	H18B-C18-H18C	109.5
C16-C19-H19A	109.5	C16-C19-H19B	109.5
H19A-C19-H19B	109.5	C16-C19-H19C	109.5
H19A-C19-H19C	109.5	H19B-C19-H19C	109.5
C15-N1-C7	118.38(19)	C15-N1-C8	124.38(18)
C7-N1-C8	115.44(19)	B1-O1-C9	107.61(18)
B1-O2-C10	107.71(18)	C15-O4-C16	121.19(18)

Table 6. Torsion angles (°) for 2l

O2-B1-C1-C4	170.5(2)	O1-B1-C1-C4	-8.6(4)
O2-B1-C1-C2	-85.0(3)	O1-B1-C1-C2	95.9(3)
C4-C1-C2-C3	-15.07(17)	B1-C1-C2-C3	-136.8(2)
C1-C2-C3-C5	135.8(2)	C1-C2-C3-C8	-96.6(2)
C1-C2-C3-C4	15.11(17)	C5-C3-C4-C1	-135.1(2)
C8-C3-C4-C1	97.6(2)	C2-C3-C4-C1	-15.28(17)
B1-C1-C4-C3	136.9(2)	C2-C1-C4-C3	15.14(17)
C8-C3-C5-C6	-55.0(3)	C4-C3-C5-C6	177.26(19)
C2-C3-C5-C6	73.6(3)	C3-C5-C6-C7	56.2(3)
C5-C6-C7-N1	-54.1(3)	C5-C3-C8-N1	53.3(3)
C4-C3-C8-N1	-175.15(18)	C2-C3-C8-N1	-78.1(2)
O1-C9-C10-O2	27.9(2)	C11-C9-C10-O2	-87.8(2)
C12-C9-C10-O2	144.6(2)	O1-C9-C10-C13	146.1(2)
C11-C9-C10-C13	30.4(3)	C12-C9-C10-C13	-97.1(3)
O1-C9-C10-C14	-86.1(2)	C11-C9-C10-C14	158.2(2)
C12-C9-C10-C14	30.7(3)	O3-C15-N1-C7	4.6(3)
O4-C15-N1-C7	-176.46(19)	O3-C15-N1-C8	168.6(2)
O4-C15-N1-C8	-12.5(3)	C6-C7-N1-C15	-139.5(2)
C6-C7-N1-C8	55.1(3)	C3-C8-N1-C15	140.4(2)
C3-C8-N1-C7	-55.2(3)	O2-B1-O1-C9	7.8(3)
C1-B1-O1-C9	-172.9(2)	C11-C9-O1-B1	97.3(2)
C12-C9-O1-B1	-144.4(2)	C10-C9-O1-B1	-22.4(3)
O1-B1-O2-C10	11.6(3)	C1-B1-O2-C10	-167.6(2)
C13-C10-O2-B1	-147.0(2)	C14-C10-O2-B1	93.1(3)
C9-C10-O2-B1	-24.6(3)	O3-C15-O4-C16	-0.3(3)
N1-C15-O4-C16	-179.22(19)	C18-C16-O4-C15	62.5(3)
C17-C16-O4-C15	-179.1(2)	C19-C16-O4-C15	-63.8(3)

Table 7. Anisotropic atomic displacement parameters (Å^2) for 2l

The anisotropic atomic displacement factor exponent takes the form: $-2\pi^2[h^2 a^{*2} U_{11} + ... + 2 h k a^* b^* U_{12}]$

	U_{11}	U_{22}	U_{33}	U_{23}	U_{13}	U_{12}
B1	0.0432(15)	0.0331(14)	0.0400(14)	-0.0008(11)	-0.0056(12)	-0.0086(11)
C1	0.0494(14)	0.0335(12)	0.0397(13)	-0.0029(10)	-0.0094(11)	-0.0085(10)
C2	0.0464(14)	0.0294(12)	0.0446(13)	-0.0003(10)	-0.0082(10)	-0.0036(10)
C3	0.0477(14)	0.0253(11)	0.0374(12)	-0.0040(9)	-0.0108(10)	-0.0003(9)
C4	0.0478(14)	0.0328(12)	0.0590(16)	-0.0094(11)	-0.0168(12)	-0.0033(10)
C5	0.084(2)	0.0348(13)	0.0354(13)	-0.0044(10)	-0.0155(13)	0.0010(13)
C6	0.082(2)	0.0411(14)	0.0283(12)	-0.0003(10)	0.0026(12)	-0.0033(13)
C7	0.0677(17)	0.0383(13)	0.0364(13)	-0.0023(10)	0.0031(12)	-0.0101(12)
C8	0.0429(13)	0.0326(12)	0.0420(13)	-0.0093(10)	-0.0012(10)	-0.0065(10)
C9	0.0504(15)	0.0325(12)	0.0489(14)	-0.0034(10)	-0.0049(11)	-0.0107(10)

	U_{11}	U_{22}	U_{33}	U_{23}	U_{13}	U_{12}
C10	0.0578(16)	0.0333(13)	0.0506(15)	-0.0038(11)	-0.0057(12)	-0.0151(11)
C11	0.0680(19)	0.0714(19)	0.0514(16)	-0.0152(14)	0.0067(14)	-0.0124(15)
C12	0.0514(17)	0.0575(17)	0.083(2)	-0.0096(15)	-0.0187(15)	-0.0131(13)
C13	0.078(2)	0.0383(15)	0.076(2)	0.0017(13)	-0.0264(16)	0.0006(13)
C14	0.103(3)	0.067(2)	0.0603(19)	-0.0042(15)	0.0296(18)	-0.0215(18)
C15	0.0437(14)	0.0302(12)	0.0378(13)	-0.0074(9)	0.0033(10)	-0.0029(10)
C16	0.0469(14)	0.0430(13)	0.0359(12)	-0.0038(10)	-0.0073(10)	-0.0067(11)
C17	0.076(2)	0.0510(16)	0.0433(15)	-0.0035(12)	0.0029(13)	-0.0119(14)
C18	0.059(2)	0.153(4)	0.064(2)	0.016(2)	-0.0024(16)	0.025(2)
C19	0.129(3)	0.0476(16)	0.0454(16)	-0.0071(13)	-0.0071(17)	-0.0263(17)
N1	0.0487(12)	0.0369(10)	0.0315(10)	-0.0085(8)	0.0040(8)	-0.0137(9)
O1	0.0707(12)	0.0339(9)	0.0599(11)	0.0014(8)	-0.0256(9)	-0.0106(8)
O2	0.0752(13)	0.0366(10)	0.0592(11)	0.0007(8)	-0.0285(9)	-0.0144(9)
O3	0.0505(11)	0.0600(11)	0.0504(10)	-0.0033(8)	0.0018(8)	-0.0241(9)
O4	0.0534(10)	0.0505(10)	0.0313(9)	-0.0073(7)	-0.0008(7)	-0.0201(8)

Table 8. Hydrogen atomic coordinates and isotropic atomic displacement parameters (Å^2) for 2l

	x/a	y/b	z/c	U(eq)
H1	0.8594	0.5671	0.6899	0.049
H2A	0.5751	0.5821	0.8530	0.048
H2B	0.5237	0.5274	0.7556	0.048
H4A	1.0831	0.4425	0.7789	0.055
H4B	1.0391	0.5118	0.8722	0.055
H5A	0.7200	0.4417	0.9769	0.062
H5B	0.8310	0.3219	0.9527	0.062
H6A	0.4763	0.2958	1.0068	0.061
H6B	0.3824	0.3881	0.9303	0.061
H7A	0.5846	0.1722	0.8914	0.057
H7B	0.3352	0.2093	0.8767	0.057
H8A	0.7569	0.3650	0.7023	0.046
H8B	0.8598	0.2739	0.7775	0.046
H11A	0.7140	0.8754	0.8970	0.094
H11B	0.8501	0.9814	0.9145	0.094
H11C	0.8983	0.8645	0.9730	0.094
H12A	1.2743	0.8753	0.9270	0.095
H12B	1.2449	0.9813	0.8539	0.095
H12C	1.3522	0.8644	0.8213	0.095
H13A	0.7610	1.0162	0.6612	0.096
H13B	0.8778	1.0670	0.7455	0.096

	x/a	y/b	z/c	U(eq)
H13C	0.6717	0.9947	0.7652	0.096
H14A	1.0875	0.9060	0.6044	0.115
H14B	1.2342	0.8314	0.6773	0.115
H14C	1.2220	0.9659	0.6784	0.115
H17A	0.5105	0.4155	0.4737	0.085
H17B	0.4676	0.3227	0.4017	0.085
H17C	0.6697	0.3052	0.4687	0.085
H18A	0.0631	0.2783	0.5787	0.141
H18B	0.0964	0.3245	0.4732	0.141
H18C	0.1493	0.4010	0.5562	0.141
H19A	0.5498	0.1184	0.5292	0.109
H19B	0.3578	0.1360	0.4569	0.109
H19C	0.3063	0.1075	0.5646	0.109

Table 9. Hydrogen bond distances (Å) and angles (°) for 2l

	Donor-H	Acceptor-H	Donor-Acceptor	Angle
C18-H18A···O3	0.97	2.47	2.998(4)	113.9
C19-H19C···O3	0.97	2.48	3.011(4)	114.5
